# Synthesis and Optical Properties of a Series of Push-Pull Dyes Based on Pyrene as the Electron Donor

**DOI:** 10.3390/molecules28031489

**Published:** 2023-02-03

**Authors:** Thanh-Tuân Bui, Sébastien Péralta, Frédéric Dumur

**Affiliations:** 1 CY Cergy Paris Université, LPPI, F-95000 Cergy, France; 2 Aix Marseille Univ CNRS, ICR UMR7273, F-13397 Marseille, France; 3 CY Cergy Paris Université, CY Advanced Studies (CY AS), F-95000 Cergy, France

**Keywords:** pyrene, polycyclic aromatic hydrocarbon, push-pull dye, solvatochromism, electron donor

## Abstract

Fifteen push-pull dyes comprising the tetracyclic polyaromatic pyrene have been designed and synthesized. The optical properties of the fifteen dyes have been examined in twenty-two solvents of different polarities. Surprisingly, contrarily to what is classically observed for push-pull dyes of D-π-A structures, a negative solvatochromism could be found for numerous dyes. The photoluminescence and thermal properties of the dyes were also examined. Theoretical calculations were carried out to support the experimental results.

## 1. Introduction

**Figure 1:**
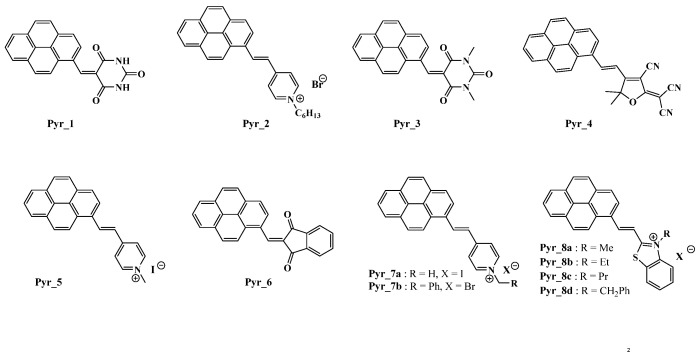
Chemical structures of push-pull dyes **Pyr_1–Pyr_6** previously reported in the literature: **Pyr_1** [92], **Pyr_2** [91], **Pyr_3** [90], **Pyr_4** [89], **Pyr_5** [88], **Pyr_6** [87], **Pyr_7** [69], **Pyr_8** [68].

**Figure 2:**
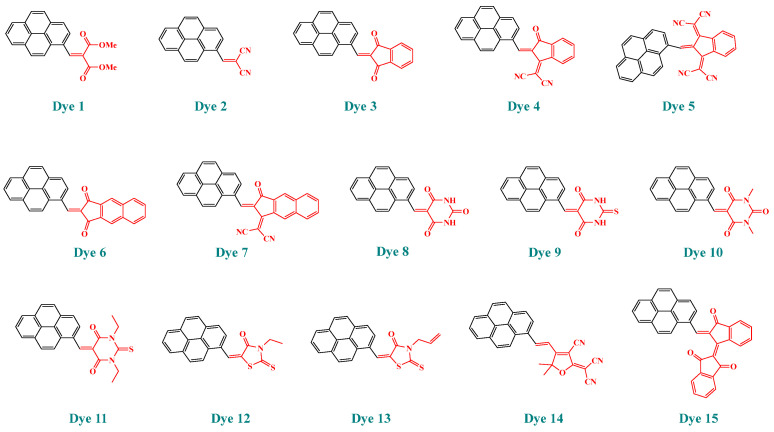
Chemical structures of the pyrene-based dyes **Dye 1–Dye 15** examined in this work.

During the last few decades, push-pull dyes have been extensively studied due to the facile tunability of their optical properties [1,2,3,4,5,6,7,8,9,10,11,12,13,14,15,16]. Among them, dyes with D-π-A structures, where D and A stand for electron donors and electron acceptors, respectively, and π for a π-conjugated spacer, are the most widely studied [8,17,18,19,20,21,22,23,24,25,26]. Indeed, for a given series of electron acceptors, an electron donor can be used as a reference donor to examine the influence of the electron-accepting group on the photophysical properties. However, the opposite situation is also true and the same electron acceptor can be used for the design of a series of dyes, variation occurring this time on the electron donor. Using these two strategies, different series of dyes have been prepared, comprising 2-(3-cyano-4,5,5-trimethylfuran-2(5*H*)-ylidene)malononitrile (TCF) [27,28,29], 2,4,5,7-tetranitrofluorene (TNF) [30,31] or 1*H*-cyclopenta[*b*]naphthalene-1,3(2*H*)-dione [32] as the electron acceptors, and Michler’s aldehyde [33], ferrocene [34], the 4,4-*bis*(4-methoxyphenyl)butadienyl donor [35] or the 4-(9-ethyl-9*H*-carbazol-3-yl)-4-phenylbuta-1,3-dienyl group [36] as the electron donors. By improvement the electron-donating ability of a donor, a red-shift of the intramolecular charge transfer band can be obtained so that dyes absorbing in the near-infrared range could be obtained, notably by using TNF as the electron acceptor [30,31]. Investigation of the optical properties of push-pull dyes is notably justified by the number of applications requiring push-pull dyes. Thus, push-pull dyes have been extensively used in photopolymerization [37,38,39,40,41,42,43], non-linear optics [4,5,44,45,46,47,48,49], light-to-energy conversion [6,50,51,52,53,54,55,56,57], biological labelling [58,59,60,61,62,63,64], light-emitting diodes [65,66,67] or cell nucleus staining [68,69]. Among electron donors that have only been scarcely used for the design of push-pull dyes, pyrene is one example. This polycyclic aromatic hydrocarbon composed of four fused aromatic rings exhibits a strong tendency to form dimers in solution [70,71,72], but also long-living excited states, so that pyrene is extensively used as a building block for the design of visible light photoinitiators of polymerization for multicomponent systems [73,74,75,76,77]. By its long-living excited state, the excited pyrene can efficiently interact with the different additives introduced into the photocurable resin. Recently, pyrene has also been used in an emerging research field, i.e., photoredox catalysis and photosynthetic systems enabling various chemical transformations were obtained [78]. As a drawback, pyrene is also considered as a pollutant whose mineralization is extensively studied [79,80,81,82]. Considering the difficulty of decomposing this polyaromatic structure, the fate of pyrene in the environment is the focus of numerous works [83,84,85,86]. Meanwhile, regarding pyrene-based push-pull dyes, examples of structures reported in the literature remain scarce. As shown in Figure 1, only twelve dyes have been reported in the literature [68,69,87,88,89,90,91,92]. Moreover, comparisons of their optical properties are difficult, the optical properties of these dyes being recorded in different solvents. Additionally, solvatochromism has not been investigated for all these dyes. Thus, their thermal properties have not been examined for all dyes. Therefore, a study in which all dyes have been investigated in similar conditions is missing. It has to be noted that the solvatochromic properties have been examined in detail for one compound, namely 6-pentafluorostyryl-1-dimethylaminopyrene, exhibiting a polarity- and a viscosity-dependent emission [93,94]. In this work, a series of fifteen pyrene-based push-pull dyes have been prepared and their optical properties examined in twenty-two solvents of different polarities (see Figure 2). The thermal properties of the different dyes were also examined. To obtain a deeper insight into the optical properties, theoretical calculations were also carried out.

## 2. Results and Discussion

### 2.1. Synthesis of **Dye 1–Dye 15**

**Scheme 1:**
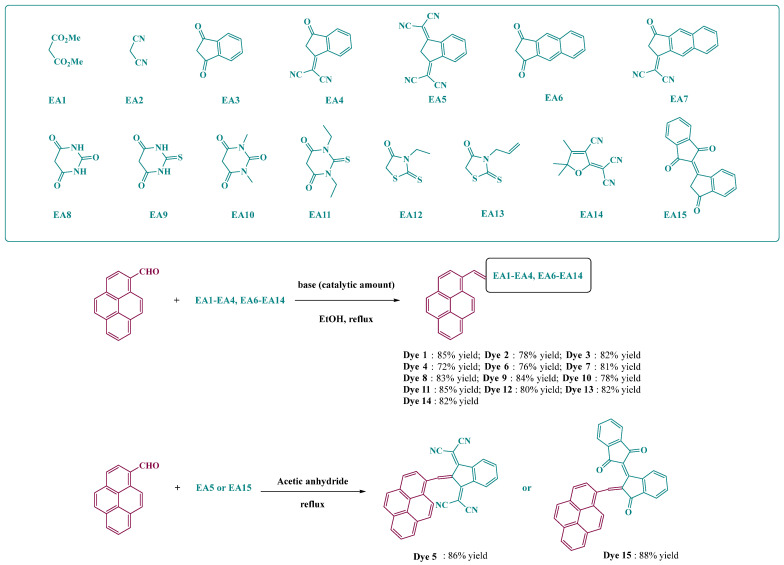
Synthetic routes to obtain **Dye 1–Dye 15**.

Two distinct synthetic strategies were developed to access the different structures, depending on the electron acceptors. Except for **Dye 5** and **Dye 15**, the different compounds **Dye 1–Dye 4**, **Dye 6–Dye 14** were obtained by a Knoevenagel reaction in basic conditions, using piperidine as the base. Conversely, for **EA5** and **EA15**, for which anions are highly stable and unreactive in basic conditions, acidic conditions had to be used and acetic anhydride was selected as the appropriate solvent [7,95,96]. Upon reflux of the solutions for two hours for **Dye 5** or heating at 90 °C overnight for **Dye 15**, **Dye 5** and **Dye 15** could be obtained, with reaction yields ranging from 86% for **Dye 5** to 88% for **Dye 15** (see Scheme 1). In turn, the fifteen dyes could be obtained in reasonable yields, ranging from 72% for **Dye 4** to 88% for **Dye 15.**

### 2.2. Optical Properties

**Figure 3:**
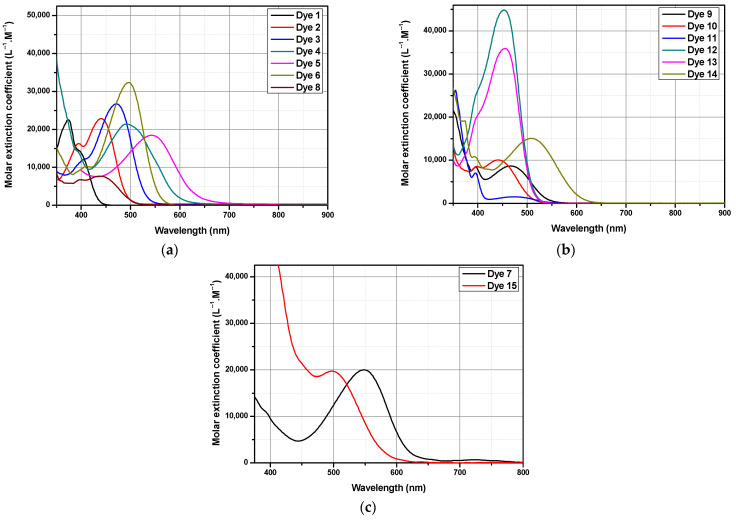
UV–visible absorption spectra of **Dye 1-Dye 4, Dye 6** and **Dye 8-Dye 14** in DMF (**a**,**b**), and **Dye 5**, **Dye 7** and **Dye 15** in dioxane (**c**).

Pyrene-based dyes are highly polyaromatic structures, so that the determination of a common solvent in which all dyes could be soluble was not possible. Interestingly, almost all dyes were soluble in *N,N*-dimethylformamide (DMF), except three dyes, i.e., **Dye 5**, **Dye 7** and **Dye 15**, for which absorption spectra were recorded in dioxane. For these three dyes, dioxane was used as the appropriate solvent for examining their optical properties. In these conditions, the optical properties of almost of the dyes could be compared in DMF. As shown in Figure 3, all dyes showed strong absorption centered in the visible range. Considering that pyrene is a weak electron donor, all dyes showed an intense absorption band in the 350–500 nm region corresponding to the intramolecular charge transfer (ICT) band. The most red-shifted absorption was found for **Dye 7** (λ_max_ = 549 nm), comprising 2-(3-oxo-2,3-dihydro-1*H*-cyclopenta[*b*]naphthalen-1-ylidene) malononitrile **EA7** as the electron acceptor. As shown in Figure 3a, the charge transfer band of **Dye 7** is broad and extends from 450 to 700 nm. Following **Dye 7**, **Dye 5**, comprising 2,2′-(1*H*-Indene-1,3(2*H*)-diylidene)dimalononitrile **EA5** as the acceptor, exhibited the second most red-shifted absorption maximum at 549 nm, outperforming all the other dyes (see Figure 3c).

**Figure 4:**
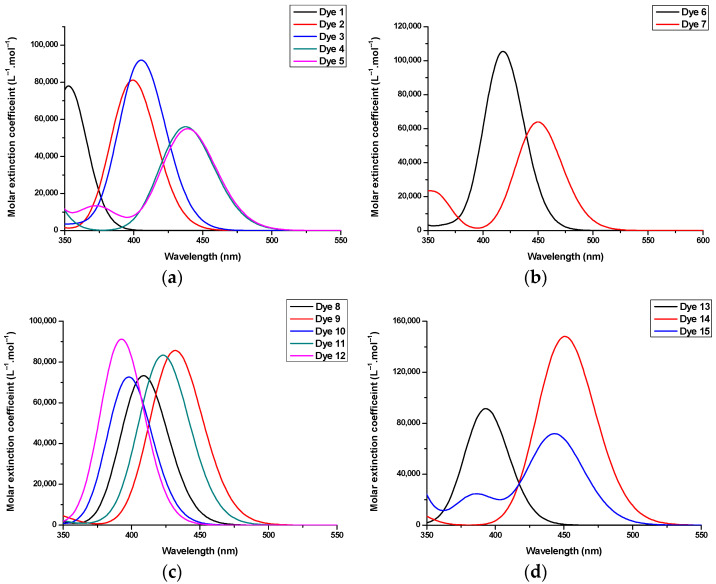
Theoretical UV–visible absorption spectra of **Dye 1–Dye 15** in DMF. (**a**) **Dye 1**–**Dye 5**; (**b**) **Dye 6**, **Dye 7**; (**c**) **Dye 8**–**Dye12**; (**d**) **Dye 13**–**Dye 15**.

These results are consistent with previous works reported in the literature evidencing the remarkable electron-withdrawing ability of this group [32,97]. As anticipated, the bluest-shifted absorption was found for **Dye 1**, comprising dimethyl malonate as the electron acceptor. Indeed, **EA1** is the weakest electron acceptor of the series. In this last case, the maximum absorption located at 374 nm could be determined. Moreover, if the absorption of this dye is strongly UV-centered, absorption could, however, be found in the visible range thanks to the long tail extending to 450 nm. The highest molar extinction coefficient of the series was determined for **Dye 12**, peaking at 44,800 L^−1^.M^−1^. A slightly lower molar extinction coefficient (ε = 35,950 L^−1^.M^−1^) was determined for **Dye 13**, also bearing a rhodanine-based electron acceptor (see Table 1). Theoretical studies were also carried out to investigate the energy levels as well as the molecular orbital (M.O.) compositions of the different dyes. DFT calculations were performed for all dyes at the wb97xd/6-311g(d,p) level of theory using the Gaussian 09 program to determine the transitions involved in the different absorption peaks. DMF was used as the solvent model with a polarizable continuum model (PCM) [98,99,100,101,102,103,104]. Theoretical UV–visible absorption spectra were obtained by TD-DFT calculations and the different spectra are presented in Figure 4. Optical characteristics are summarized in Table 1. The position of the absorption maxima was determined from the theoretical spectra. As shown in Table 1, for all dyes, the positions of the theoretical absorption maxima were determined in DMF as the solvent. Noticeably, all theoretical absorption maxima were blue-shifted compared to the experimental ones. In fact, the PCM model only allows us to create an electrostatic field corresponding to the dielectric nature of the solvent around the molecule. In no case does this model allow us to take into account specific interactions between the molecule and the solvent. Notably, it does not allow us to take into account a protic effect of a solvent, for instance. In fact, no solvate model in DFT allows us to take into account easily the molecule/solvent interactions. As a consequence of this, a mismatch between the theoretical and the experimental positions of the absorption maxima is found.

**Figure 5:**
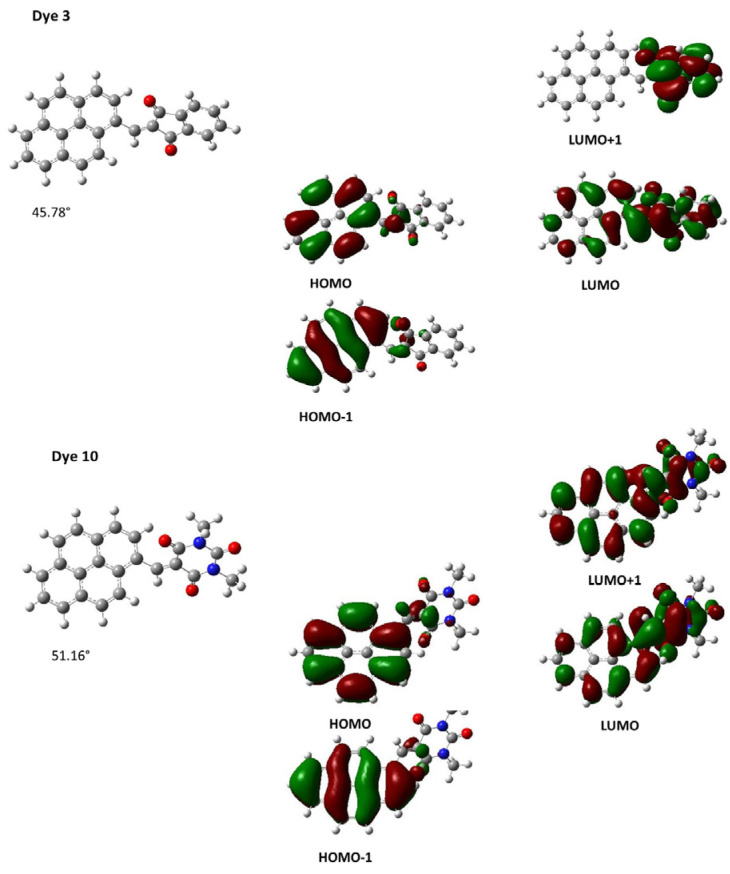
Contour plots of the HOMO and LUMO orbitals of **Dye 3** (top) and **Dye 10** (bottom).

As anticipated, the minor variations of the substitution pattern of **Dye 12** and **Dye 13** did not modify the UV–visible absorption spectra, as experimentally observed. Examination of the contour plots of the Highest Occupied Molecular Orbital (HOMO) and the Lowest Unoccupied Molecular Orbital (LUMO) revealed a classical electronic distribution for the two orbitals. As shown in Figure 5, logically, the HOMO orbital is located on the electron donor, whereas the LUMO level is centered on the electron-accepting one.

**Figure 6:**
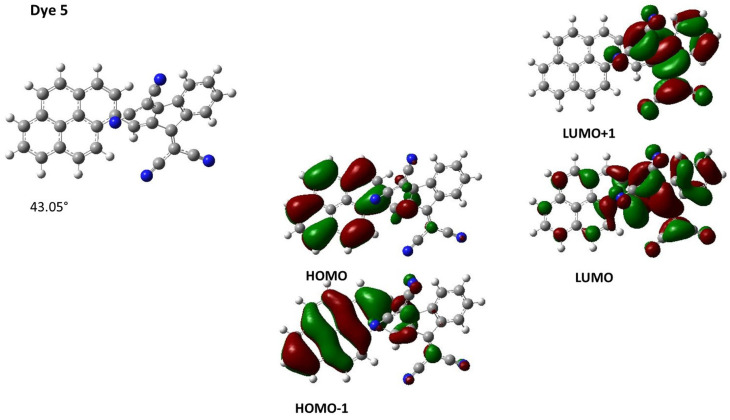
Three-dimensional view of the optimized geometry for **Dye 5** and contour plots of the HOMO and LUMO orbitals.

**Figure 7:**
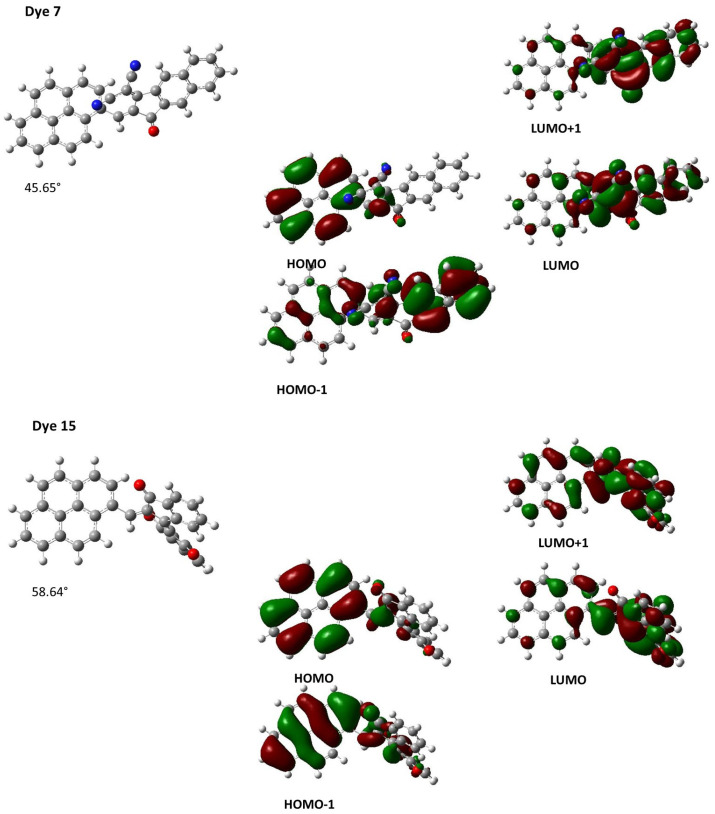
Contour plots of the HOMO and LUMO orbitals of **Dye 7** (top) and **Dye 15** (bottom).

To obtain a deeper insight into the different transitions involved in the intramolecular charge transfer bands of the different dyes, theoretical calculations were carried out and the different data are summarized in Table 2. Notably, for **Dye 5**, which comprises the bulkiest electron acceptor, the intramolecular charge transfer band was determined as being an admixture of HOMO => LUMO, HOMO => LUMO + 1 and HOMO − 1 => LUMO transitions. Optimization of the geometry for **Dye 5** also revealed this dye to exhibit strong internal torsion with a dihedral angle of 43.05° due to the steric hindrance generated by the electron acceptor (see Figure 6). Moreover, despite this internal torsion, a HOMO energy level extending over the pyrene moiety and the dicyanomethylene groups of the acceptor could be determined (see Figure 6). Similarly, the LUMO energy level of **Dye 5** is mainly located on the electron acceptor but also extends over the pyrene moiety, demonstrating an electronic communication that is maintained between the donor and the acceptor. A similar distribution of both the HOMO and LUMO energy levels can be evidenced for **Dye 7** and **Dye 15**, also comprising sterically hindered electron acceptors (see Figure 7). In these cases, torsion angles of 45.65° and 58.64° were determined between the donors and acceptors in **Dye 7** and **Dye 15**, respectively.

**Scheme 2:**
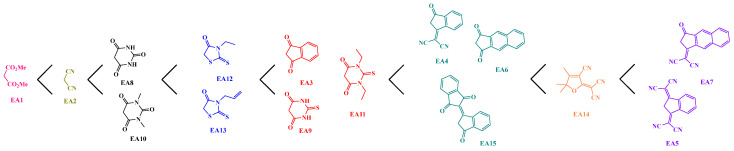
Classification of the electron acceptors based on the position of the ICT bands.

For all dyes, the position of the theoretical absorption maximum was close to the position determined for the HOMO => LUMO transition by TD-DFT, since a difference of a few nanometers was found. Therefore, the main transition involved at the absorption maximum corresponds to a HOMO => LUMO transition. Moreover, the exciton binding energy of the different dyes, which is defined as the difference between the electrochemical and optical bandgaps, could not be determined [105,106], the dyes being not sufficiently soluble to determine with accuracy the positions of the HOMO and LUMO energy levels by electrochemistry. Based on the position of the ICT bands, the different electron acceptors could be classified according to the order presented in Scheme 2. Among all electron acceptors, **EA7** and **EA5** were determined as exhibiting the highest electron-withdrawing abilities of the series of 15 electron acceptors, consistent with the ordering previously reported in the literature [33]. The weakest electron acceptors were determined as being **EA1** and **EA2** [107,108]. Notably, **EA1** is rarely used for the design of push-push dyes due to the weak electronic delocalization that this electron acceptor involves [87,109,110,111].

### 2.3. Solvatochromism

**Figure 8:**
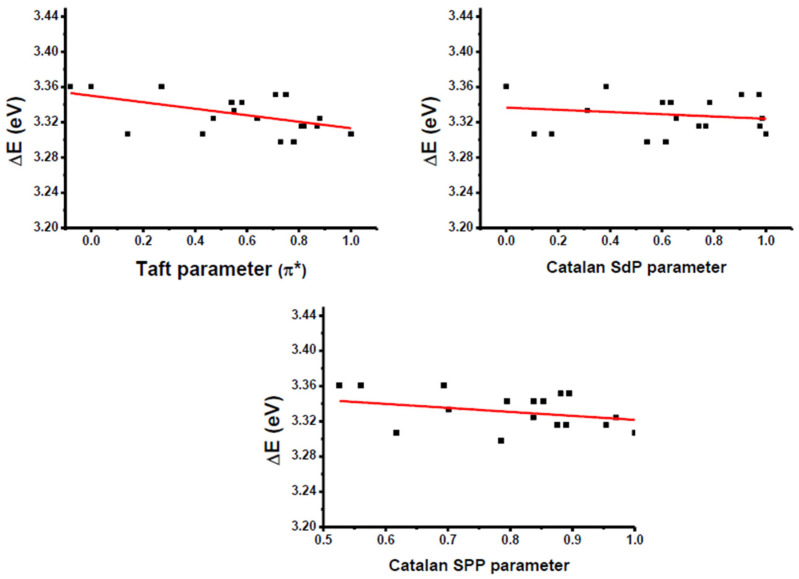
Linear regressions obtained using different polarity scales for **Dye 1**.

**Figure 9:**
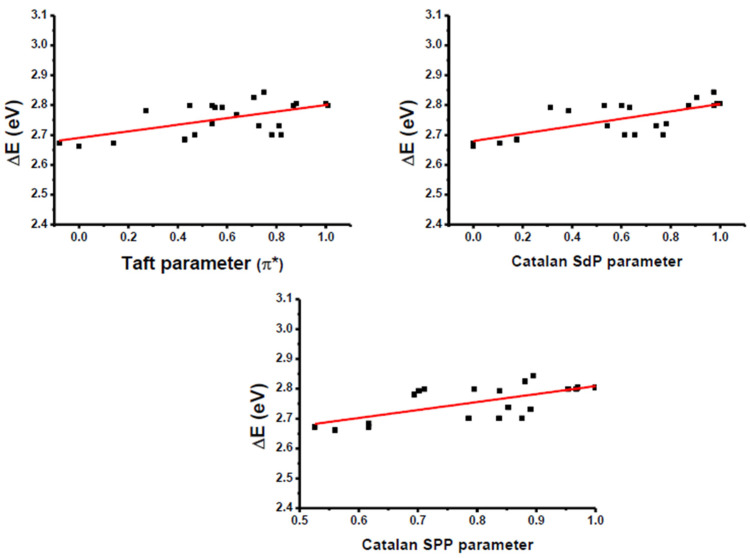
Linear regressions obtained using different polarity scales for **Dye 10**.

Despite the polyaromatic nature of the electron donor and the low solubility of the different push-pull dyes in numerous solvents, the solvatochromism of the fifteen dyes could, however, be examined in a wide range of solvents differing by their polarities. A summary of the optical properties of the fifteen dyes, **Dye 1–Dye 15**, is provided in Table 3. Solvatochromism corresponds to a charge redistribution upon excitation, and, in this field, several empirical solvent polarity scales have been developed over the years. Among these, Kamlet–Taft’s [112], Catalan’s [113], Kawski–Chamma Viallet’s [114], Lippert–Mataga’s [115], McRae–Suppan’s [116], Dimroth–Reichardt’s [117], and Bakhshiev’s [118] scales have been developed by various research groups in order to rationalize the solvatochromism observed experimentally. As the main parameters governing the solvatochromism, the polarity and the polarizability of the solvents can be cited as the main parameters governing the modification of the optical bandgaps. Classically, a reduction in the HOMO–LUMO gap (where HOMO and LUMO denote the Highest Occupied Molecular Orbital and Lowest Unoccupied Molecular Orbital, respectively) is observed upon the increase in the solvent polarity. In the present case, linear correlations could be obtained with only two scales, i.e., the Kamlet–Taft and the Catalan scales. Two distinct behaviors could be clearly identified. Thus, for dyes such as **Dye 1**, **Dye 2**, **Dye 5**, **Dye 6**, **Dye 12** and **Dye 14**, a positive solvatochromism could be determined, notably using the Kamlet–Taft polarity scale (See Figure 8). This behavior is observed for numerous dyes of D-π-A structures and is indicative of an excited state more polar than the ground one. However, the opposite situation could also be found for numerous dyes, as exemplified for **Dye 3**, **Dye 4**, **Dye 8–Dye 11** and **Dye 13**, and a negative solvatochromism could be clearly evidenced using the same polarity scales (see Figure 9). Noticeably, **Dye 8** and **Dye 9**, which are the non-alkylated versions of **Dye 10** and **Dye 11**, exhibited a similar solvatochromism to that observed for **Dye 10** and **Dye 11**, for which electron acceptors are alkylated. Moreover, in the case of **Dye 8** and **Dye 9**, a different behavior of the electron acceptors can be anticipated compared to **Dye 10** and **Dye 11**. Indeed, in the case of **Dye 8** and **Dye 9**, electron-withdrawing groups **EA8** and **EA9** can switch between the enol and the keto forms depending on the environment [119]. As a result of this equilibrium, the electron-withdrawing abilities of **EA8** and **EA9** differ from one solvent to another, complicating the solvatochromism of **Dye 8** and **Dye 9**. Based on the literature, the negative solvatochromism detected for **Dye 8** and **Dye 9** is not unusual for (thio)barbituric dyes. Indeed, negative solvatochromisms for (thio)barbituric dyes comprising the enolizable electron acceptors **EA8** or **EA9** have already been reported in the literature [119,120]. If non-enolizable, **EA10** and **EA11** are nonetheless able to generate hydrogen bonds between molecules, generating complex supramolecular structures in solution [121]. In particular, these supramolecular structures can be strongly impacted by the hydrogen-bonding ability of the solvent so that a complex solvatochromism can also be evidenced for these structures. In turn, a negative solvatochromism was determined for **Dye 8–Dye 11**.

This unexpected behavior was thus observed mostly for the dyes elaborated with the strongest electron-withdrawing groups. Typically, negative solvatochromism is often observed for betaine dyes and assigned to a modification of the relative electrophilicities of both the electron-pair donor and acceptor moieties with the solvent, so that irregular behaviors can be found for this family of dyes [122,123,124]. To the best of our knowledge, such a behavior has never been reported for polyaromatic electron donors. This unexpected behavior can also be tentatively assigned to the formation of pyrene-based dimers with different orientations (cross, g-like, slip or stack), the formation of aggregates of various sizes in solution, affecting the analysis of their solvatochromism. In fact, π–π stacking interactions between polyacene structures have been extensively studied in the literature [125,126,127,128,129,130,131,132,133,134,135,136]. Upon π–π stacking interactions, various orientations can be found between stacked molecules: cross, g-like, slip or stack [137]. Overall, these noncovalent interactions between molecules govern the optical properties of the resulting solutions, which comprise isolated molecules, and the concomitant presence of stacked dyes that adversely impact the rationalization of the solvatochromism [138]. In particular, the negative solvatochromism is observed for the less soluble dyes, supporting this hypothesis of the concomitant presence of isolated molecules and aggregates. Indeed, for highly soluble dyes such as **Dye 1** and **Dye 2**, bearing small electron acceptors, a classical behavior is evidenced for these dyes. In the case of **Dye 8** and **Dye 9**, negative solvatochromism can also be assigned to the hydrogen bond ability of the two electron acceptors (presence of NH groups), affecting the solvatochromism of these dyes. Moreover, in the case of **Dye 10** and **Dye 11**, a negative solvatochromism is also evidenced for these two dyes, despite the alkylation of the electron acceptors.

### 2.4. Photoluminescence Properties

**Figure 10:**
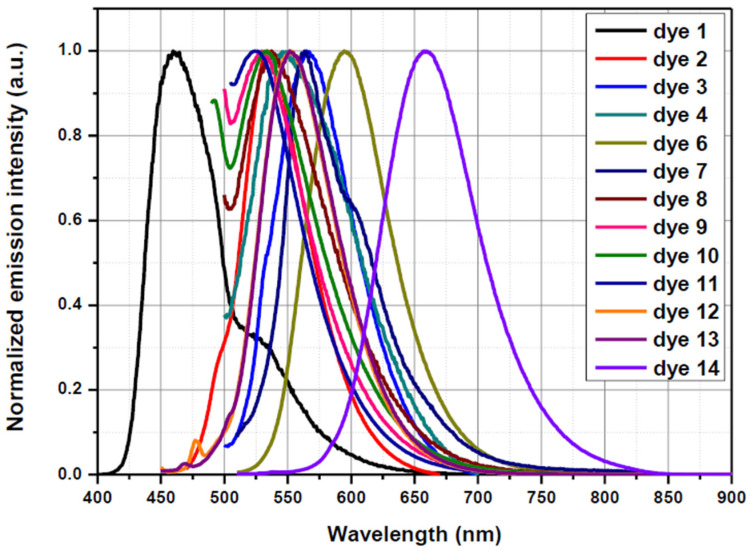
Fluorescence spectra of **Dye 1–Dye 14** recorded in DMF as the solvent.

The photoluminescence of all dyes was examined in dichloromethane and in DMF and the different results are summarized in Table 1. As shown in Figure 10, major differences could be determined for the different dyes. Noticeably, the most blue-shifted emission was determined for **Dye 1**, bearing the weakest electron acceptor (λ_max_ = 461 nm), whereas the most-redshifted absorption was found for **Dye 14**, bearing **EA14** as the electron acceptor. As shown in Figure 10, an emission extending until the near-infrared range was found for **Dye 14**. Following **Dye 14**, the second most red-shifted absorption was determined for **Dye 6**, comprising **EA6**. Interestingly, the largest Stokes shift was determined for **Dye 14**, being 152 nm. Indeed, for this dye, an absorption located at 507 nm and an emission at 659 nm was found in DMF. If this Stokes shift is important, it remains, however, lower than that reported for 6-pentafluorostyryl-1-dimethylaminopyrene, recently reported in the literature and exhibiting a Stokes shift of 247 nm [93]. This exceptional value can be assigned to the presence of the dimethylamino group on pyrene, improving the electronic delocalization between pyrene and the electron acceptor. For the rest of the molecules, Stokes shifts ranging between 80 and 100 nm could be found, except for **Dye 7**, for which a Stokes shift of only 14 nm was calculated. **Dye 14** thus constitutes an excellent candidate as a fluorescent probe for various applications, in light of the large Stokes shift detected for this dye. It has to be noted that comparison of the Stokes shift obtained in dichloromethane and DMF revealed the Stokes shift to be more important in the more polar solvent [94]. This trend is consistent with the previous works reported in the literature mentioning the higher sensitivity of the emission of pyrene-based dyes to the solvent polarity than the absorption and reporting a red shift of the emission maximum with the solvent polarity, which is also observed in this study.

### 2.5. Thermal Properties

**Figure 11:**
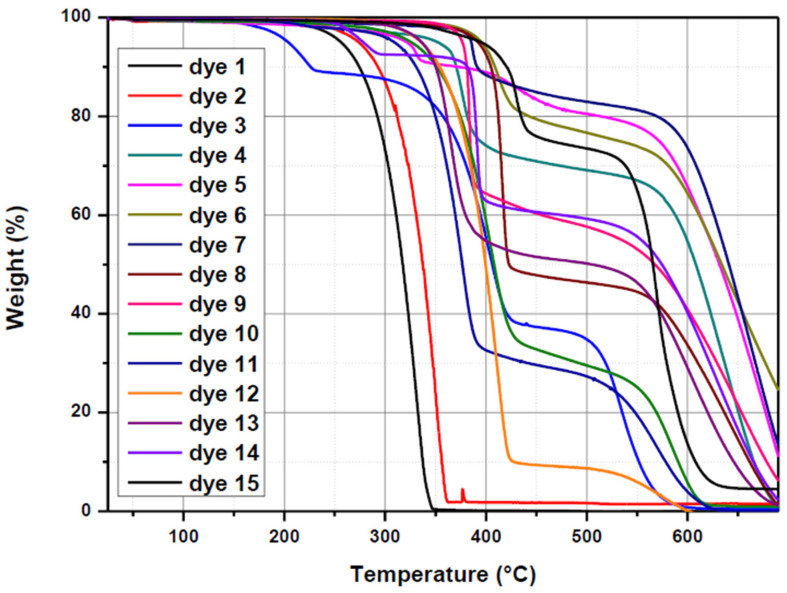
Thermogravimetric analysis (TGA) thermograms of **Dye 1–Dye 15** recorded under nitrogen at a scan rate of 20 °C/min.

For numerous applications, the thermal stability of dyes is an important parameter, notably for applications such as solar cells [139,140,141,142]. The thermal properties of the different dyes were examined by thermal gravimetry analyses, and a summary of the decomposition temperatures is given in Table 4 and in Figure 11. Noticeably, despite the presence of the same electron-donating group, major differences could be determined concerning the decomposition temperatures of **Dye 1–Dye 15**. The lowest one was determined for **Dye 3**, at 176 °C. Conversely, the highest one was found for **Dye 15**, determined at 400 °C. Only three dyes showed decomposition temperatures lower than 300 °C, evidencing their remarkable thermal stability.

## 3. Materials and Methods

### 3.1. General Information

All reagents and solvents were purchased from Aldrich, Alfa Aesar or TCI Europe and used as received, without further purification. Mass spectroscopy was performed by the Spectropole of Aix Marseille University. ESI mass spectral analyses were recorded with a 3200 QTRAP (Applied Biosystems SCIEX) mass spectrometer. The HRMS mass spectral analysis was performed with a QStar Elite (Applied Biosystems SCIEX) mass spectrometer. Elemental analyses were recorded with a Thermo Finnigan EA 1112 elemental analysis apparatus driven by the Eager 300 software.

^1^H and ^13^C NMR spectra (More details could be found in Supplementary Materials) were determined at room temperature in 5 mm o.d. tubes on a Bruker Avance 400 spectrometer of the Spectropole: ^1^H (400 MHz) and ^13^C (100 MHz). The ^1^H chemical shifts were referenced to the solvent peak CDCl_3_ (7.26 ppm) and the ^13^C chemical shifts were referenced to the solvent peak CDCl_3_ (77 ppm).

2-(3-oxo-2,3-dihydro-1*H*-inden-1-ylidene)malononitrile **EA4** [143], 2,2′-(1*H*-indene-1,3(2*H*)-diylidene)dimalononitrile **EA5** [144], 1*H*-cyclo-penta[*b*]naphthalene-1,3(2*H*)-dione **EA6** [32,145], 2-(3-oxo-2,3-dihydro-1*H*-cyclopenta[*b*]naphthalen-1-ylidene)malononitrile **EA7 [145,146]**, 2-(3-cyano-4,5,5-trimethylfuran-2(5*H*)-ylidene) malononitrile **EA6** [27,29,147,148] and [1,2′]biindenylidene-3,1′,3′-trione **EA15** [149] were prepared as previously reported in the literature, without modification and in similar yields.

Thermal properties of the different dyes were investigated by using a TA thermal analyzer (TA Instrument Q50) at a heating rate of 20 °C/min under argon flow. The temperature of thermal degradation (T_d_) was measured at the point of 5% weight loss. UV–visible absorption spectra were recorded on a Varian Cary 60 UV–vis spectrophotometer with a concentration of 5 × 10^−3^ M, corresponding to diluted solutions. Fluorescence spectra were recorded on a Jasco spectrofluorometer FP-8350. Melting points were determined with a Buchi Melting Point M-560 at a scan rate of 10 °C/min by varying the temperature between 80 and 350 °C.

### 3.2. Synthesis of the Dyes

General procedure for the synthesis of all dyes (except **Dye 5** and **Dye 15**): 1-pyrenecarbaldehyde (2 g, 8.68 mmol) and the appropriate electron acceptor (8.68 mmol, 1 eq.) were dissolved in absolute ethanol (50 mL). A few drops of piperidine were added. Immediately, the solution’s color changed. The solution was refluxed and monitoring of the reaction progress was carried out by thin layer chromatography (TLC). After cooling, the solution was concentrated under reduced pressure. Addition of pentane precipitated a solid, which was filtered off and dried under vacuum.

Dimethyl 2-(pyren-1-ylmethylene)malonate **(Dye 1)**

85% yield. ^1^H NMR (300 MHz, CDCl_3_) δ 8.81 (s, 1H), 8.30–7.92 (m, 9H), 3.97 (s, 3H), 3.72 (s, 3H); ^13^C NMR (75 MHz, CDCl_3_) δ 167.05 (C=O), 164.57 (C=O), 142.05 (CH=C(CO_2_Me)_2_), 132.75, 131.14, 130.63, 129.81, 128.79, 128.71, 127.75, 127.20, 127.18, 126.33, 126.14, 126.05, 125.53, 124.68, 124.59, 124.36, 122.89, 52.79 (OMe), 52.56 (OMe); HRMS (ESI MS) m/z: theor: 344.1049 found: 344.1042 ([M]^+.^ detected); Mp = 146 °C.

2-(Pyren-1-ylmethylene)malononitrile **(Dye 2)**

78% yield. ^1^H NMR (300 MHz, DMSO) δ 9.62 (s, 1H), 8.71 (t, *J* = 9.2 Hz, 2H), 8.54–8.37 (m, 5H), 8.37–8.18 (m, 2H); HRMS (ESI MS) m/z: theor: 278.0844 found: 278.0844 ([M]^+.^ detected); Anal. Calc. for C_20_H_10_N_2_: C, 86.3; H, 3.6; O, 10.1; Found: C, 86.4; H, 3.7; N, 9.7%; Td > 240 °C.

2-(Pyren-1-ylmethylene)-1*H*-indene-1,3(2*H*)-dione **(Dye 3)**

82% yield. ^1^H NMR (400 MHz, CDCl_3_) δ 9.34 (d, *J* = 8.2 Hz, 1H), 9.11 (s, 1H), 8.63 (d, *J* = 9.3 Hz, 1H), 8.31–8.28 (m, 2H), 8.28–8.23 (m, 2H), 8.21 (d, *J* = 8.9 Hz, 1H), 8.12 (d, *J* = 8.9 Hz, 1H), 8.10–8.03 (m, 3H), 7.87–7.81 (m, 2H); HRMS (ESI MS) m/z: theor: 358.0994 found: 358.0996 ([M]^+.^ detected); Anal. Calc. for C_26_H_14_O_2_: C, 87.1; H, 3.9; O, 8.9; Found: C, 87.4; H, 3.7; N, 8.7%; Td > 140 °C.

2-(3-Oxo-2-(pyren-1-ylmethylene)-2,3-dihydro-1*H*-inden-1-ylidene)malononitrile **(Dye 4)**

72% yield. HRMS (ESI MS) m/z: theor: 406.1106 found: 406.1103 ([M]^+^ detected); Anal. Calc. for C_29_H_14_N_2_ O: C, 85.7; H, 3.5; O, 3.9; Found: C, 85.4; H, 3.7; N, 3.7%; Td > 200 °C.

2-(Pyren-1-ylmethylene)-1*H*-cyclopenta[*b*]naphthalene-1,3(2*H*)-dione **(Dye 6)**

76% yield. HRMS (ESI MS) m/z: theor: 408.1150 found: 408.1157 ([M]^+.^ detected); Anal. Calc. for C_30_H_16_O_2_: C, 88.2; H, 3.9; O, 7.8; Found: C, 88.4; H, 3.9; N, 8.1%; Mp = 314 °C.

2-(3-Oxo-2-(pyren-1-ylmethylene)-2,3-dihydro-1*H*-cyclo-penta[*b*]naphthalen-1-ylidene)malononitrile **(Dye 7)**

81% yield. HRMS (ESI MS) m/z: theor: 456.1263 found: 456.1256 ([M]^+.^ detected); Anal. Calc. for C_33_H_16_N_2_O: C, 86.8; H, 3.5; O, 3.5; Found: C, 86.8; H, 3.6; N, 3.6 %; Td > 200 °C.

5-(Pyren-1-ylmethylene)pyrimidine-2,4,6(1*H*,3*H*,5*H*)-trione **(Dye 8)**

83% yield. ^1^H NMR (400 MHz, DMSO) δ 11.50 (s, 1H), 11.21 (s, 1H), 9.11 (s, 1H), 8.42–8.11 (m, 9H); HRMS (ESI MS) m/z: theor: 340.0848 found: 340.0842 ([M]^+.^ detected); Anal. Calc. for C_21_H_12_N_2_O_3_: C, 74.1; H, 3.5; O, 14.1; Found: C, 74.4; H, 3.7; N, 14.2%; Td > 200 °C.

5-(Pyren-1-ylmethylene)-2-thioxodihydropyrimidine-4,6(1*H*, 5*H*)-dione **(Dye 9)**

84% yield. ^1^H NMR (400 MHz, DMSO) δ 12.57 (s, 1H), 12.33 (s, 1H), 9.14 (s, 1H), 8.42 (dd, *J* = 12.8, 7.9 Hz, 3H), 8.31 (dd, *J* = 15.2, 8.6 Hz, 3H), 8.23 (dd, *J* = 9.0, 3.4 Hz, 2H), 8.14 (t, *J* = 7.6 Hz, 1H); HRMS (ESI MS) m/z: theor: 356.0619 found: 356.0619 ([M]^+.^ detected); Anal. Calc. for C_21_H_12_N_2_O_2_S: C, 70.8; H, 3.4; O, 9.0; Found: C, 70.6; H, 3.3; N, 9.2%; Td > 200 °C.

1,3-Dimethyl-5-(pyren-1-ylmethylene)pyrimidine-2,4,6(1*H*,3*H*, 5*H*)-trione **(Dye 10)**

78% yield. ^1^H NMR (400 MHz, CDCl_3_) δ 9.56 (s, 1H), 8.47 (d, *J* = 8.2 Hz, 1H), 8.26 (d, *J* = 7.6 Hz, 2H), 8.21–8.14 (m, 4H), 8.11–8.01 (m, 2H), 3.51 (s, 3H), 3.35 (s, 3H); ^13^C NMR (101 MHz, CDCl_3_) δ 162.44 (C=O), 160.05 (C=O), 157.60 (C=O), 151.47 (=CH vinyl), 134.27, 131.08, 131.04, 130.56, 129.65, 129.32, 128.75, 127.38, 127.22, 126.75, 126.66, 126.36, 124.37, 123.75, 123.34, 118.56, 29.03 (N-Me), 28.40 (N-Me); HRMS (ESI MS) m/z: theor: 368.1161 found: 368.1165 ([M]^+.^ detected); Td > 200 °C.

1,3-Diethyl-5-(pyren-1-ylmethylene)-2-thioxodihydro-pyrimi-dine-4,6(1*H*,5*H*)-dione **(Dye 11)**

85% yield. ^1^H NMR (400 MHz, CDCl_3_) δ 9.57 (s, 1H), 8.55 (d, *J* = 8.2 Hz, 1H), 8.27 (dd, *J* = 15.9, 6.5 Hz, 4H), 8.20 (t, *J* = 7.7 Hz, 2H), 8.13–8.04 (m, 2H), 4.66 (q, *J* = 6.9 Hz, 2H), 4.53 (q, *J* = 6.9 Hz, 2H), 1.41 (t, *J* = 7.0 Hz, 3H), 1.29 (t, *J* = 7.0 Hz, 3H); ^13^C NMR (75 MHz, CDCl_3_) δ 179.12 (C=S), 160.73 (C=O), 158.29 (C= O), 158.14 (=CH vinyl), 134.65, 131.44, 131.04, 130.52, 129.93, 129.47, 129.03, 127.39, 127.32, 126.93, 126.84, 126.41, 124.32, 124.30, 123.81, 123.36, 118.88, 44.15 (OCH_2_CH_3_), 43.62 (OCH_2_CH_3_), 12.53 (OCH_2_CH_3_), 12.47 (OCH_2_CH_3_); HRMS (ESI MS) m/z: theor: 412.1245 found: 412.1242 ([M]^+.^ detected); Td > 200 °C.

3-Ethyl-5-(pyren-1-ylmethylene)-2-thioxothiazolidin-4-one **(Dye 12)**

80% yield. ^1^H NMR (300 MHz, CDCl_3_) δ 8.76 (s, 1H), 8.42 (d, *J* = 9.3 Hz, 1H), 8.28–8.11 (m, 5H), 8.05 (t, *J* = 6.9 Hz, 3H), 4.27 (q, *J* = 7.1 Hz, 2H), 1.36 (t, *J* = 7.1 Hz, 3H); ^13^C NMR (75 MHz, CDCl_3_) δ 193.66 (C=S), 167.40 (C=O), 133.03 (=CH vinyl), 131.27, 130.97, 130.66, 130.01, 129.53, 129.24, 127.25, 126.91, 126.60, 126.56, 126.43, 125.66, 125.09, 125.01, 124.91, 124.34, 122.35, 39.84 (NCH_2_CH_3_), 12.32 (NCH_2_CH_3_); HRMS (ESI MS) m/z: theor: 373.05955 found: 373.0590 ([M]^+.^ detected); Mp = 215 °C

3-Allyl-5-(pyren-1-ylmethylene)-2-thioxothiazolidin-4-one **(Dye 13)**

82% yield. ^1^H NMR (400 MHz, CDCl_3_) δ 8.81 (s, 1H), 8.46 (d, *J* = 9.3 Hz, 1H), 8.29–8.04 (m, 8H), 5.99–5.88 (m, 1H), 5.34 (ddd, *J* = 13.7, 11.4, 1.2 Hz, 2H), 4.83 (dt, *J* = 5.9, 1.3 Hz, 2H); HRMS (ESI MS) m/z: theor: 385.0595 found: 385.0598 ([M]^+.^ detected); Anal. Calc. for C_23_H_15_NOS_2_: C, 71.7; H, 3.9; O, 4.1; Found: C, 71.4; H, 3.7; N, 4.4%; Mp = 195 °C.

2-(3-Cyano-5,5-dimethyl-4-(2-(pyren-1-yl)vinyl)furan-2(5*H*)-ylidene)malononitrile **(Dye 14)**

82% yield. Due to its insolubility, no ^1^H NMR spectrum could be acquired. HRMS (ESI MS) m/z: theor: 411.1372 found: 411.1373 ([M]^+.^ detected); Anal. Calc. for C_28_H_17_N_3_O: C, 81.7; H, 4.2; O, 3.9; Found: C, 81.4; H, 3.9; N, 4.1%; Td > 200 °C.

Synthesis of 2,2′-(2-(pyren-1-ylmethylene)-1*H*-indene-1,3(2*H*)-diylidene) dimalononitrile **(Dye 5)**

2,2′-(1*H*-Indene-1,3(2*H*)-diylidene)dimalononitrile (1.22 g, 5.02 mmol, M = 242.24 g/mol) and pyrene-1-carbaldehyde (1.15 g, 5.02 mmol, M = 230.27 g/mol) were dissolved in acetic anhydride (20 mL) and the solution was refluxed for two hours. After cooling, the solvent was removed under reduced pressure. Addition of ether followed by pentane precipitated a blue solid that was filtered off, washed several times with pentane and dried under vacuum (1.96 g, 86% yield).

^1^H NMR (400 MHz, CDCl_3_) δ 9.56 (s, 1H), 8.74 (dd, *J* = 5.9, 3.0 Hz, 1H), 8.65 (dd, *J* = 5.9, 2.9 Hz, 1H), 8.37 (s, 2H), 8.33 (t, *J* = 7.9 Hz, 2H), 8.25 (d, *J* = 8.9 Hz, 1H), 8.17 (d, *J* = 8.1 Hz, 1H), 8.11 (dd, *J* = 12.7, 5.1 Hz, 2H), 7.94 (d, *J* = 8.1 Hz, 1H), 7.91 (dd, *J* = 6.6, 2.9 Hz, 2H); HRMS (ESI MS) m/z: theor: 454.1218 found: 454.1218 ([M]^+.^ detected); Anal. Calc. for C_32_H_14_N_4_: C, 84.6; H, 3.1; N, 12.3; Found: C, 84.4; H, 2.7; N, 12.4%; Td > 200 °C

Synthesis of 2-(pyren-1-ylmethylene)-[1,2′-biindenylidene]-1′,3,3′(2*H*)-trione **(Dye 15)**

To a mixture of [1,2′]-biindenylidene-3,1′,3′-trione (0.5 g, 1.28 mmol, M = 274.28 g/mol) and 2-pyrenecarbaldehyde (0.42 g, 1.28 mmol, 230.27 g/mol) was added acetic anhydride so that the powder was covered by the liquid. The reaction mixture was heated at 90 °C overnight. After cooling, ether was added. A red precipitate formed. It was filtered off, washed several times with ether and dried under vacuum to remove the traces of acetic anhydride (0.55 g, 88% yield).

^1^H NMR (400 MHz, CDCl_3_) δ 9.39 (d, *J* = 7.5 Hz, 1H), 9.30 (dd, *J* = 17.0, 7.9 Hz, 2H), 8.05 (d, *J* = 7.4 Hz, 1H), 7.89–7.79 (m, 3H), 7.75 (dt, *J* = 19.1, 6.1 Hz, 3H), 7.68 (d, *J* = 7.7 Hz, 1H), 7.61 (t, *J* = 7.1 Hz, 1H), 7.58–7.49 (m, 2H), 7.39 (t, *J* = 7.4 Hz, 1H), 7.28 (s, 3H); HRMS (ESI MS) m/z: theor: 486.1256 found: 486.1251 ([M]^+.^ detected); Anal. Calc. for C_35_H_18_O_3_: C, 86.4; H, 3.7; O, 9.9; Found: C, 86.4; H, 3.9; N, 10.1%; Td > 200 °C.

## 4. Conclusions

To conclude, a series of fifteen dyes based on pyrene as the electron donor have been designed and synthesized. Noticeably, all dyes could be prepared using standard synthetic procedures, enabling us to obtain the different compounds in reasonable yields. Examination of their solvatochromic properties in 22 solvents of different polarities revealed the most soluble dyes to exhibit a positive solvatochromism. On the contrary, for the less soluble dyes, the opposite trend was found, and a negative solvatochromism was detected for these dyes. This unexpected behavior was assigned to the formation of aggregates of different sizes and shapes in solution, the formation of hydrogen bonds between molecules for all dyes possessing electron-withdrawing groups comprising NH or C=O groups affecting in turn the examination of their solvatochromic properties, as the isolated molecules were concomitantly present with aggregates and hydrogen-bonded molecules. All dyes also showed high thermal stability, and a decomposition temperature higher than 300 °C was determined for most of the dyes. Considering that all dyes strongly absorb in the visible range and that pyrene derivatives are excellent photoinitiators of polymerization, future works will consist of examining their photoinitiating abilities during the free radical polymerization of acrylates upon visible light irradiation.

## Figures and Tables

**Table 1:** Optical characteristics of the different compounds in DMF, dioxane and dichloromethane and theoretically determined.

**Experimental Data**															
	**Dye 1**	**Dye 2**	**Dye 3**	**Dye 4**	**Dye 5**	**Dye 6**	**Dye 7**	**Dye 8**	**Dye 9**	**Dye 10**	**Dye 11**	**Dye 12**	**Dye 13**	**Dye 14**	**Dye 15**
λ_abs_ (nm) ^a^	374	442	471	496	543	496	549	442	467	442	477	454	452	507	497
ϵ (M^−1^·cm^−1^)	22,300	22,900	26,500	21,600	18,200	32,200	19,925	7500	8500	9800	1600	44,800	35,950	14,800	19,500
λ_em_ (nm) ^a^	462	535	567	546	713	594	563	539	-	533	529	550	552	659	-
λ_exc_ (nm) ^a^	360	440	460	420	640	480	520	400	-	380	400	380	380	440	-
Δλ (nm) ^a^	88	93	96	50	170	83	14	97	-	91	52	96	100	152	-
λ_abs_ (nm) ^b^	374	453	480	533	554	504	-	478	489	459	492	460	461	525	500
λ_em_ (nm) ^b^	446	529	554	542	642	579	-	-	-	544	575	541	539	635	545
λ_exc_ (nm) ^b^	380	460	460	400	500	480	-	-	-	400	400	380	440	460	420
Δλ (nm) ^b^	72	76	74	9	138	75	-	-	-	85	83	81	78	110	45
**Theoretical Data**															
	**Dye 1**	**Dye 2**	**Dye 3**	**Dye 4**	**Dye 5**	**Dye 6**	**Dye 7**	**Dye 8**	**Dye 9**	**Dye 10**	**Dye 11**	**Dye 12**	**Dye 13**	**Dye 14**	**Dye 15**
λ_abs_ (nm)_th_	353	399	405	438	439	418	451	408	432	398	422	392	392	451	442
ϵ (M^−1^·cm^−1^)	77,500	80,950	92,300	55,850	55,200	105,800	62,950	73,350	85,600	72,200	83,100	91,300	91,300	147,900	71,650
λ_abs_ (nm)_exp_	374	442	471	496	543	496	549	442	467	442	477	454	452	507	497

^a^ values determined in DMF, except for **Dye 5**, **Dye 7** and **Dye 15**, determined in dioxane. ^b^ values determined in dichloromethane.

**Table 2:** Summary of the simulated absorption characteristics in dilute DMF of synthetized compounds. Data were obtained in DMF solution.

Compounds	E_HOMO_ (th) (eV)	E_LUMO_ (th) (eV)	λ_max_ (nm)	λ (nm)	Transitions
**Dye 1**	−7.563	−1.069	353	352	HOMO − 1 => LUMO (94%)
**Dye 2**	−7.712	−1.705	399	399	HOMO => LUMO (94%)
**Dye 3**	−7.605	−1.445	405	405	HOMO => LUMO (87%)
**Dye 4**	−7.623	−1.784	438	437	HOMO => LUMO (83%)
**Dye 5**	−7.647	−1.829	439	439	HOMO => LUMO (84%)
**Dye 6**	−7.582	−1.823	418	418	HOMO => LUMO (84%)
**Dye 7**	−7.619	−1.905	451	450	HOMO => LUMO (84%)
**Dye 8**	−7.650	−1.670	408	408	HOMO => LUMO (91%)
**Dye 9**	−7.676	−1.885	432	422	HOMO => LUMO (88%)
**Dye 10**	−7.616	−1.318	398	397	HOMO => LUMO (90%)
**Dye 11**	−7.626	−1.783	422	423	HOMO => LUMO (88%)
**Dye 12**	−7.480	−1.460	392	392	HOMO => LUMO (82%)
**Dye 13**	−6.197	−1.260	392	392	HOMO => LUMO (82%)
**Dye 14**	−7.519	−2.089	451	450	HOMO => LUMO (88%)
**Dye 15**	−7.572	−1.759	442	443	HOMO => LUMO (61%)

**Table 3:** Summary of the optical properties of the fifteen dyes in 23 different solvents recorded at room temperature.

	Dye 1	Dye 2	Dye 3	Dye 4	Dye 5	Dye 6	Dye 7	Dye 8	Dye 9	Dye 10	Dye 11	Dye 12	Dye 13	Dye 14	Dye 15
acetone	370	437	465	-	-	491	-	439	461	439	471	448	450	506	491
acetonitrile	370	437	464	-	530	489	-	442	453	436	471	445	451	507	498
AcOEt	371	437	471	516	527	493	-	446	468	443	474	449	452	502	493
anisole	376	453	482	537	554	505	565	463	498	454	495	463	465	526	507
butanol	373	446	482	-	589	504	-	-	-	459	493	458	460	524	-
chloroform	376	459	488	544	571	512	580	486	-	459	504	463	464	529	-
cyclohexane	369	446	479	-	-	498	-	-	-	466	493	435	459	-	-
1,2-dichloroethane	374	450	477	531	550	501	562	471	503	454	489	460	461	523	499
dichloromethane	374	453	480	533	554	504	-	478	489	459	492	460	461	525	500
diethyl carbonate	-	437	472	520	535	495	-	454	473	443	476	444	453	498	
diethyl ether	369	443	472	-	-	493	-	-	-	446	485	454	456	-	488
diglyme	373	440	474	525	542	497	551	445	477	448	485	455	457	515	497
1,4-dioxane	372	435	474	523	543	495	549	443	474	444	476	444	446	505	497
dimethylacetamide	373	441	472	-	578	498	-	439	465	442	476	458	456	518	495
DMF	374	441	472	496	553	497	-	438	458	443	475	456	455	514	494
DMSO	375	441	473	-	534	498	-	432	470	442	480	458	458	517	-
ethanol	371	442	477	-	-	500	-	-	-	453	485	455	454	518	-
heptane	369	444	477	-	-	495	-	-	-	464	491	456	460	-	-
nitrobenzene	-	456	487	-	567	511	565	-	504	443	499	458	469	538	506
THF	371	439	472	520	534	494	-	439	468	444	475	452	454	512	496
toluene	375	451	483	542	562	505	566	472	500	464	497	465	465	-	506
triethylamine	370	445	-	-	-	-	-	462	513	497	-	459	460	-	-
*p*-xylene	375	451	483	540	562	505	565	471	501	462	498	465	467	518	508

**Table 4:** Decomposition temperatures of the different dyes.

Dye	Dye 1	Dye 2	Dye 3	Dye 4	Dye 5	Dye 6	Dye 7	Dye 8	Dye 9	Dye 10	Dye 11	Dye 12	Dye 13	Dye 14	Dye 15
T (°C)	243	245	176	358	318	374	376	370	356	298	307	317	319	259	400

## Data Availability

Not applicable.

## Supplementary Materials

The following supporting information can be downloaded at: https://www.mdpi.com/article/10.3390/molecules28031489/s1, Figure S1. chemical structures of the dyes examined in this work; Figures S2–S20. ^1^H/^13^C NMR spectra of the dyes; Figures S21–S35. UV-visible absorption spectra of **Dye 1–Dye 15**; Figures S36–S49. Position of the absorption maxima of **PP1–PP15** in twenty-three solvents of different polarities vs. the Kamlet–Taft parameters π*; Figures S50–S62. Position of the absorption maxima of **PP1–PP15** in twenty-three solvents of different polarities vs. the Catalan solvent polarity/polarizability (SPP) scale and the Catalan solvent dipolarity (SdP) scale; Figures S63–S77.Optimized geometries and HOMO LUMO electronic distribution of all compounds. Figures S78–S92. TGA thermograms of the different dyes; Table S1. Summary of the optical properties of **Dye 1–Dye 8** in twenty-three solvents, values of the Kamlet and Taft parameters π* and values of the Catalan solvent polarizability (SP), solvent dipolarity (SdP) and solvent polarity/polarizability (SPP) parameters; Table S2. Summary of the optical properties of **Dye 9–Dye 15** in twenty-three solvents, values of the Kamlet and Taft parameters π* and values of the Catalan solvent polarizability (SP), solvent dipolarity (SdP) and solvent polarity/polarizability (SPP) parameters; Table S3. Solvent-independent correlation coefficient s of the Kamlet-Taft parameters π*; Table S4. Solvent-independent correlation coefficients a and b of the Catalan parameters SdP and SPP; Table S5. Energy levels of the main orbitals for dyes **Dye 1–Dye 15**; Table S6. Main transitions observed for dyes **Dye 1–Dye 15**.

## Sample Availability

Samples of the compounds are available from the authors.
