# Supplementary material for: Synthesis and Optical Properties of a Series of Push-Pull Dyes Based on Pyrene as the Electron Donor
Source: Molecules. 2023 Feb 3;28(3):1489. doi: 10.3390/molecules28031489 (PMC9920555; doi:10.3390/molecules28031489)

Synthesis of the different dyes 2

UV-visible absorption spectra in solvents of different polarities 21

Variation of the absorption maxima vs. the Kamlet–Taft parameters 29

Variation of the absorption maxima vs. the Catalan (SPP/SdP) scales 36

Summary of the optical properties in 23 solvents 48

Contour plots of the HOMO and LUMO energy levels of dyes 53

Main transitions observed for dyes Dye 1-Dye 15 61

TGA measurements 66

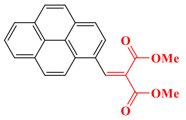
Dye 1

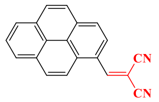
Dye 2

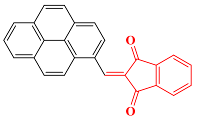
Dye 3

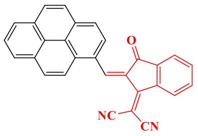
Dye 4

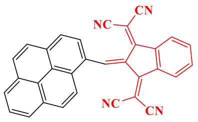
Dye 5

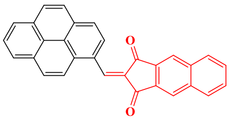
Dye 6

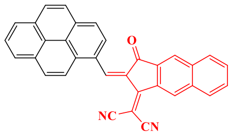
Dye 7

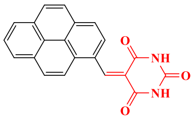
Dye 8

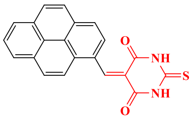
Dye 9

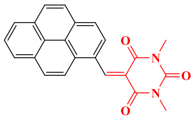
Dye 10

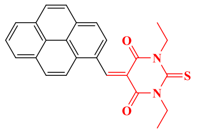
Dye 11

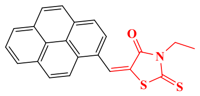
Dye 12

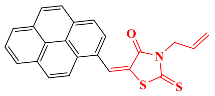
Dye 13

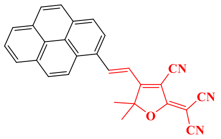
Dye 14

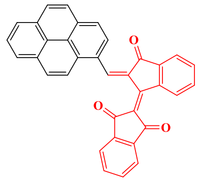
Dye 15

Figure S1. chemical structures of the dyes examined in this work.

Experimental part

All reagents and solvents were purchased from Aldrich, Alfa Aesar or TCI Europe and used as received without further purification. Mass spectroscopy was performed by the Spectropole of Aix-Marseille University. ESI mass spectral analyses were recorded with a 3200 QTRAP (Applied Biosystems SCIEX) mass spectrometer. The HRMS mass spectral analysis was performed with a QStar Elite (Applied Biosystems SCIEX) mass spectrometer. Elemental analyses were recorded with a Thermo Finnigan EA 1112 elemental analysis apparatus driven by the Eager 300 software. ^{1}H and ^{13}C NMR spectra were determined at room temperature in 5 mm o.d. tubes on a Bruker Avance 400 spectrometer and on a Bruker Avance 300 spectrometer of the Spectropole: The ^{1}H chemical shifts were referenced to the solvent peak $CDCl_3$ (7.26 ppm) and the ^{13}C chemical shifts were referenced to the solvent peak $CDCl_3$ (77 ppm).

Synthesis of dimethyl 2-(pyren-1-ylmethylene)malonate **Dye 1**

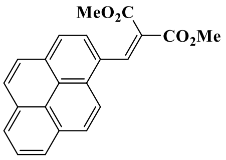

Chemical Formula: $C_{22}H_{16}O_4$
Molecular Weight: 344.3660

1-Pyrenecarbaldehyde (2 g, 8.68 mmol, M = 230.26 g/mol), dimethyl malonate (1.14 g, 8.68 mmol, M = 132.11 g/mol) were dissolved in absolute ethanol (50 mL). A few drops of piperidine were added. Immediately, the solution turned orange. The solution was refluxed for 4 hours. The solution was concentrated under reduced pressure. Addition of pentane precipitated a light-yellow solid which was filtered off and dried under vacuum (2.54 g, 85% yield). ^{1}H NMR (300 MHz, $CDCl_3$) δ 8.81 (s, 1H), 8.30 – 7.92 (m, 9H), 3.97 (s, 3H), 3.72 (s, 3H); ^{13}C NMR (75 MHz, $CDCl_3$) δ 167.05, 164.57, 142.05, 132.75, 131.14, 130.63, 129.81, 128.79, 128.71, 127.75, 127.20, 127.18, 126.33, 126.14, 126.05, 125.53, 124.68, 124.59, 124.36, 122.89, 52.79, 52.56; HRMS (ESI MS) m/z: theor: 344.1049 found: 344.1042 ($[M]^{+\cdot}$ detected).

Figure S2. ^{1}H NMR spectrum of **Dye 1**

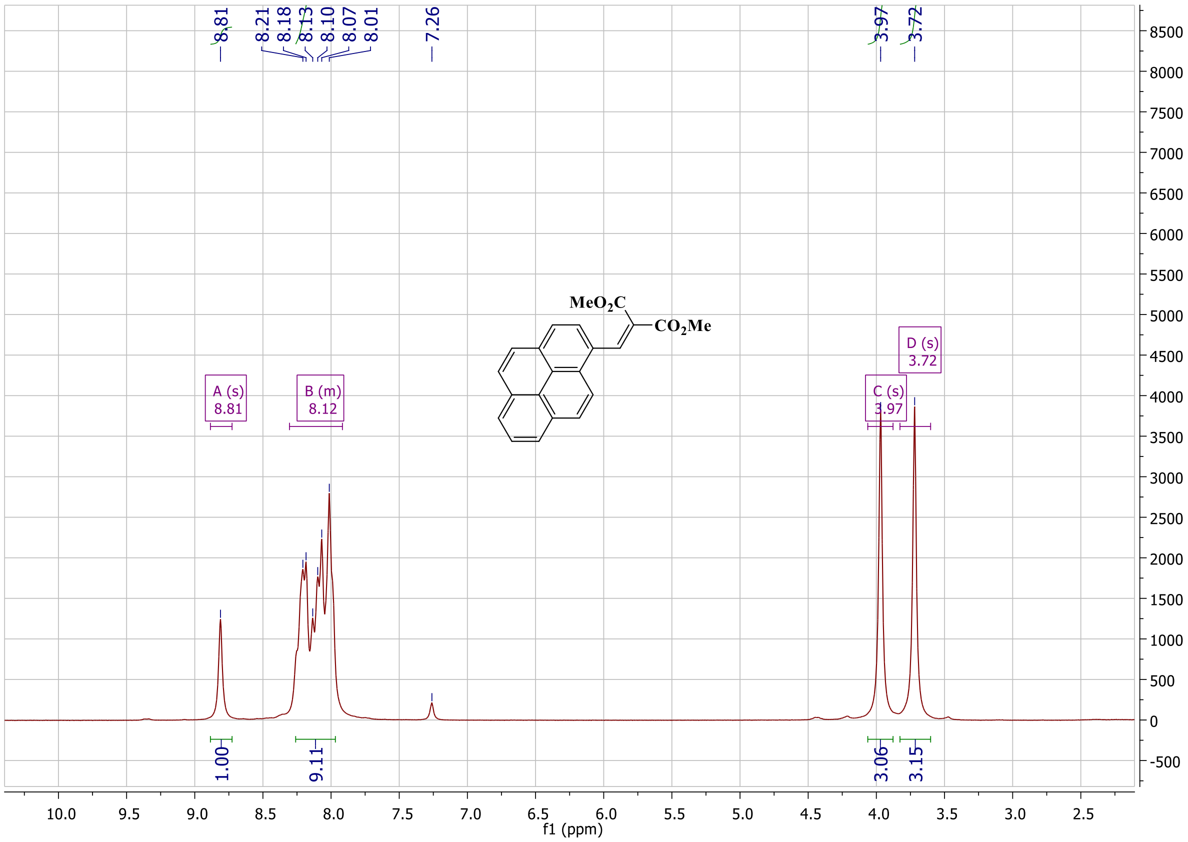

Figure S3. ^{13}C NMR spectrum of **Dye 1**

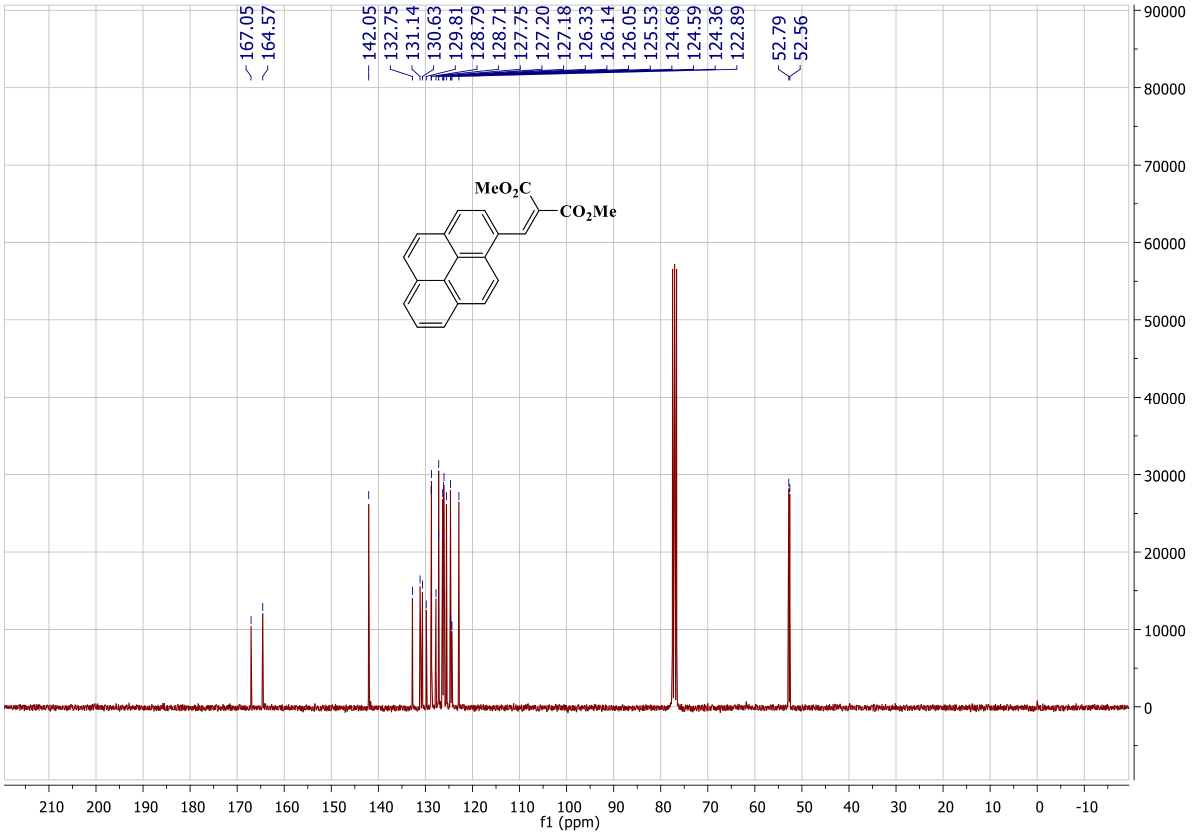

Synthesis of 2-(pyren-1-ylmethylene)malononitrile **Dye 2**

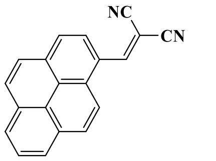

Chemical Formula: $C_{20}H_{10}N_2$
Molecular Weight: 278.3140

1-Pyrenecarbaldehyde (2 g, 8.68 mmol, M = 230.26 g/mol), malononitrile (0.57 g, 8.68 mmol, M = 66.06 g/mol) were dissolved in absolute ethanol (50 mL). A few drops of piperidine were added. Immediately, the solution turned orange. The solution was refluxed for 4 hours. After cooling, the precipitate was filtered off, washed several times with ether and dried under vacuum (1.88 g, 78% yield). ^{1}H NMR (300 MHz, DMSO) δ 9.62 (s, 1H), 8.71 (t, J = 9.2 Hz, 2H), 8.54 – 8.37 (m, 5H), 8.37 – 8.18 (m, 2H); HRMS (ESI MS) m/z: theor: 278.0844 found: 278.0844 ($[M]^{+\cdot}$ detected); Anal. Calc. for $C_{20}H_{10}N_2$: C, 86.3; H, 3.6; O, 10.1; Found: C, 86.4; H, 3.7; N, 9.7 %

Figure S4. ^{1}H NMR spectrum of **Dye 2**

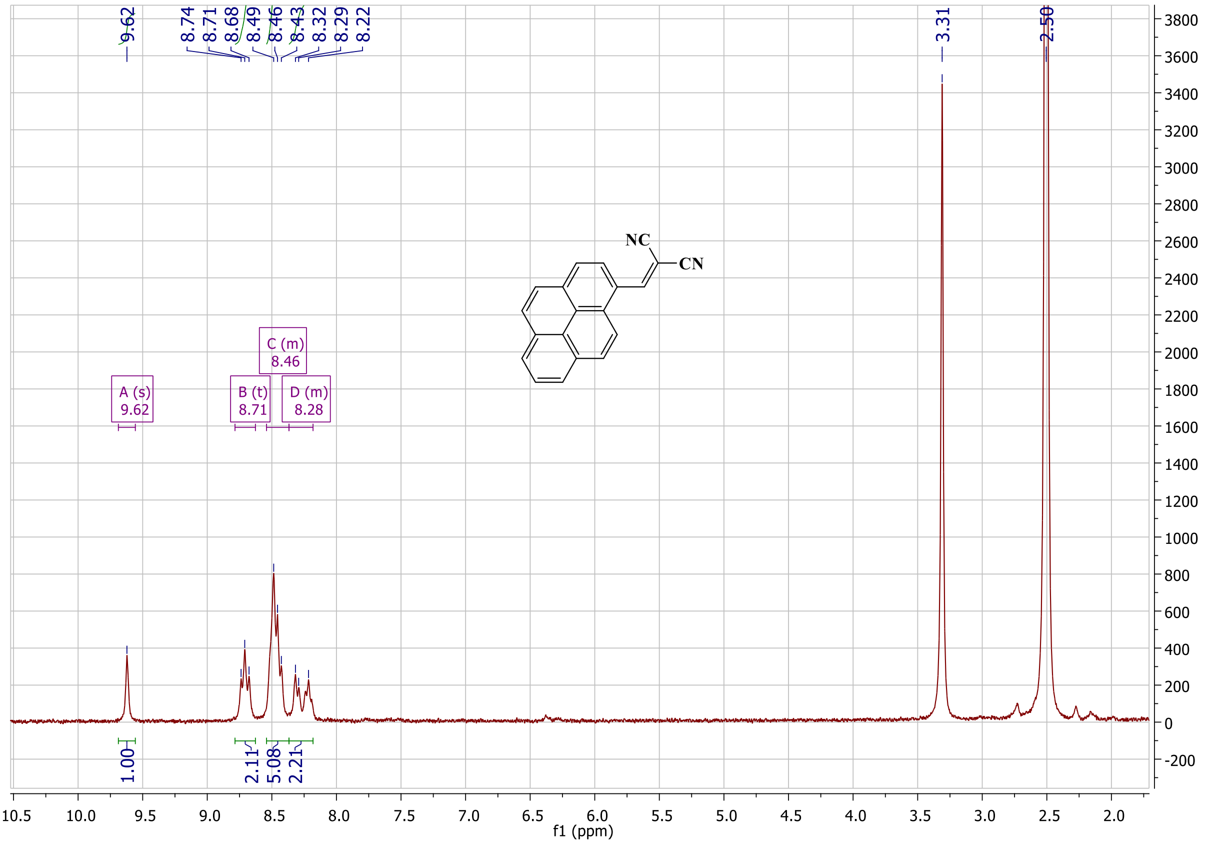

Synthesis of 2-(pyren-1-ylmethylene)-1*H*-indene-1,3(2*H*)-dione **Dye 3**

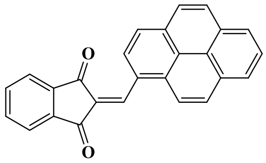

Chemical Formula: $C_{26}H_{14}O_2$
Molecular Weight: 358.3960

Pyrene 1-carbaldehyde (1.57 g, 6.84 mmol, M = 230.26 g/mol) and indane-1,3-dione (1 g, 6.84 mmol, M = 146.14 g/mol) were suspended in 20 mL absolute ethanol and a few drops of piperidine were added. Immediately, the solution turned deep red. The solution was refluxed for 3 hours. During that time, an extremely insoluble precipitate formed. After cooling, the precipitate was filtered off, washed several times with ethanol and ether, and dried under vacuum (2.01 g, 82% yield). ^{1}H NMR (400 MHz, $CDCl_3$) δ 9.34 (d, *J* = 8.2 Hz, 1H), 9.11 (s, 1H), 8.63 (d, *J* = 9.3 Hz, 1H), 8.31 – 8.28 (m, 2H), 8.28 – 8.23 (m, 2H), 8.21 (d, *J* = 8.9 Hz, 1H), 8.12 (d, *J* = 8.9 Hz, 1H), 8.10 – 8.03 (m, 3H), 7.87 – 7.81 (m, 2H); HRMS (ESI MS) m/z: theor: 358.0994 found: 358.0996 ($[M]^{+\cdot}$ detected); Anal. Calc. for $C_{26}H_{14}O_2$: C, 87.1; H, 3.9; O, 8.9; Found: C, 87.4; H, 3.7; N, 8.7 %

Figure S5. ^{1}H NMR spectrum of **Dye 3**

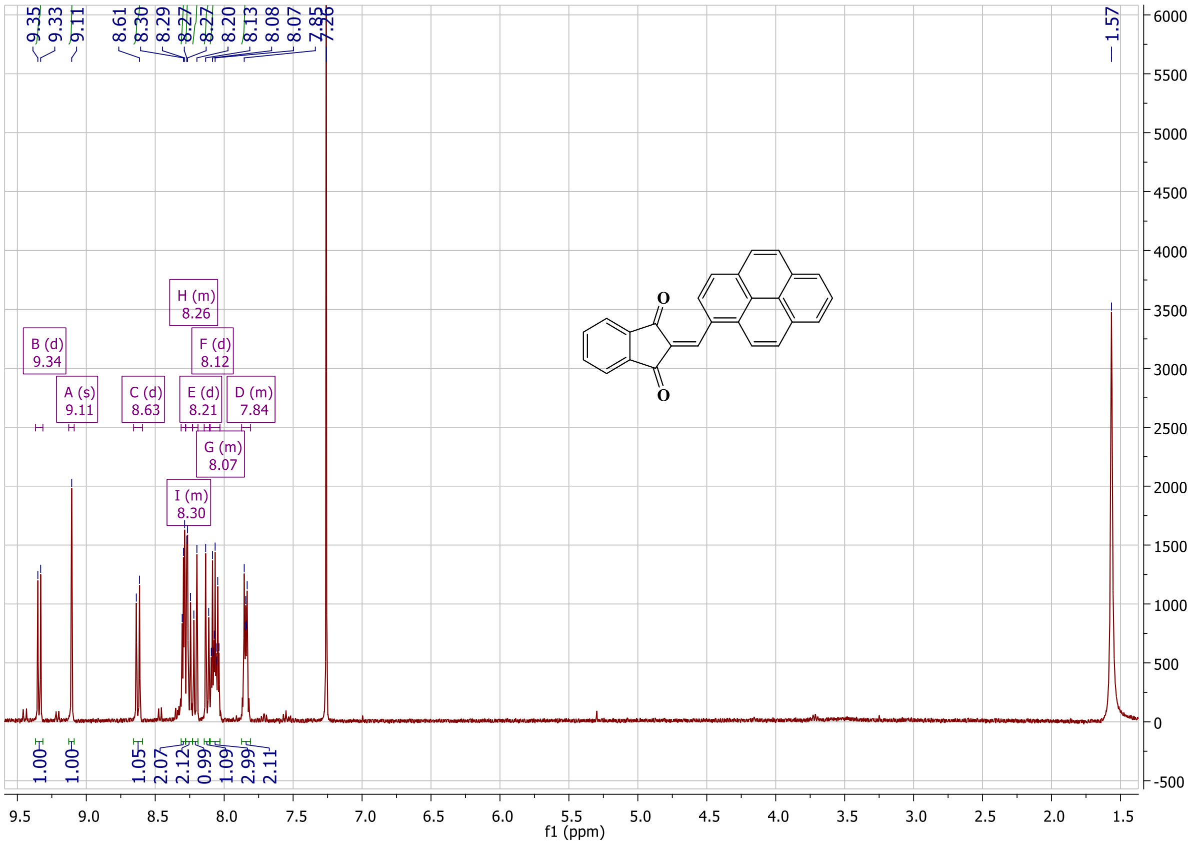

Synthesis of 2-(3-oxo-2-(pyren-1-ylmethylene)-2,3-dihydro-1*H*-inden-1-ylidene)malononitrile **Dye 4**

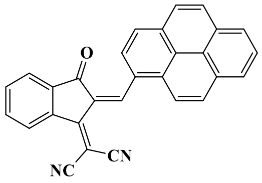

Chemical Formula: $C_{29}H_{14}N_2O$
Molecular Weight: 406.4440

1-Pyrenecarboxaldehyde (1.18 g, 5.15 mmol, M = 230.27 g/mol) and 2-(3-oxo-2,3-dihydro-1*H*-inden-1-ylidene)malononitrile (1 g, 5.15 mmol, M = 194.19 g/mol) were suspended in 20 mL absolute ethanol and a few drops of piperidine were added. Immediately, the solution turned deep red. The flask was introduced in an oil bath preheated at 90°C. After 15 min, the reaction was ended (TLC control). During that time, an extremely insoluble precipitate formed. After cooling, the precipitate was filtered off, washed several times with ethanol and ether. Due to its high insolubility, no ^{1}H NMR spectrum could be acquired (1.51 g, 72% yield). HRMS (ESI MS) m/z: theor: 406.1106 found: 406.1103 ($[M]^{+\cdot}$ detected); Anal. Calc. for $C_{29}H_{14}N_2O$: C, 85.7; H, 3.5; O, 3.9; Found: C, 85.4; H, 3.7; N, 3.7 %

Synthesis of 2,2'-(2-(pyren-1-ylmethylene)-1*H*-indene-1,3(2*H*)-diylidene)dimalononitrile **Dye 5**

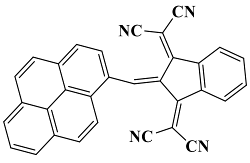

Chemical Formula: $C_{32}H_{14}N_4$
Molecular Weight: 454.4920

2,2'-(1*H*-Indene-1,3(2*H*)-diylidene)dimalononitrile (1.22 g, 5.02 mmol, M = 242.24 g/mol) and pyrene-1-carbaldehyde (1.15 g, 5.02 mmol, M = 230.27 g/mol) were dissolved in acetic anhydride (20 mL) and the solution was refluxed for two hours. After cooling, the solvent was removed under reduced pressure. Addition of ether followed by pentane precipitated a blue solid that was filtered off, washed several times with pentane and dried under vacuum (1.96 g, 86% yield). ^{1}H NMR (400 MHz, $CDCl_3$) δ 9.56 (s, 1H), 8.74 (dd, *J* = 5.9, 3.0 Hz, 1H), 8.65 (dd, *J* = 5.9, 2.9 Hz, 1H), 8.37 (s, 2H), 8.33 (t, *J* = 7.9 Hz, 2H), 8.25 (d, *J* = 8.9 Hz, 1H), 8.17 (d, *J* = 8.1 Hz, 1H), 8.11 (dd, *J* = 12.7, 5.1 Hz, 2H), 7.94 (d, *J* = 8.1 Hz, 1H), 7.91 (dd, *J* = 6.6, 2.9 Hz, 2H); HRMS (ESI MS) m/z: theor: 454.1218 found: 454.1218 ($[M]^{+\cdot}$ detected); Anal. Calc. for $C_{32}H_{14}N_4$: C, 84.6; H, 3.1; N, 12.3; Found: C, 84.4; H, 2.7; N, 12.4 %

Figure S6. ^{1}H NMR spectrum of **Dye 5**

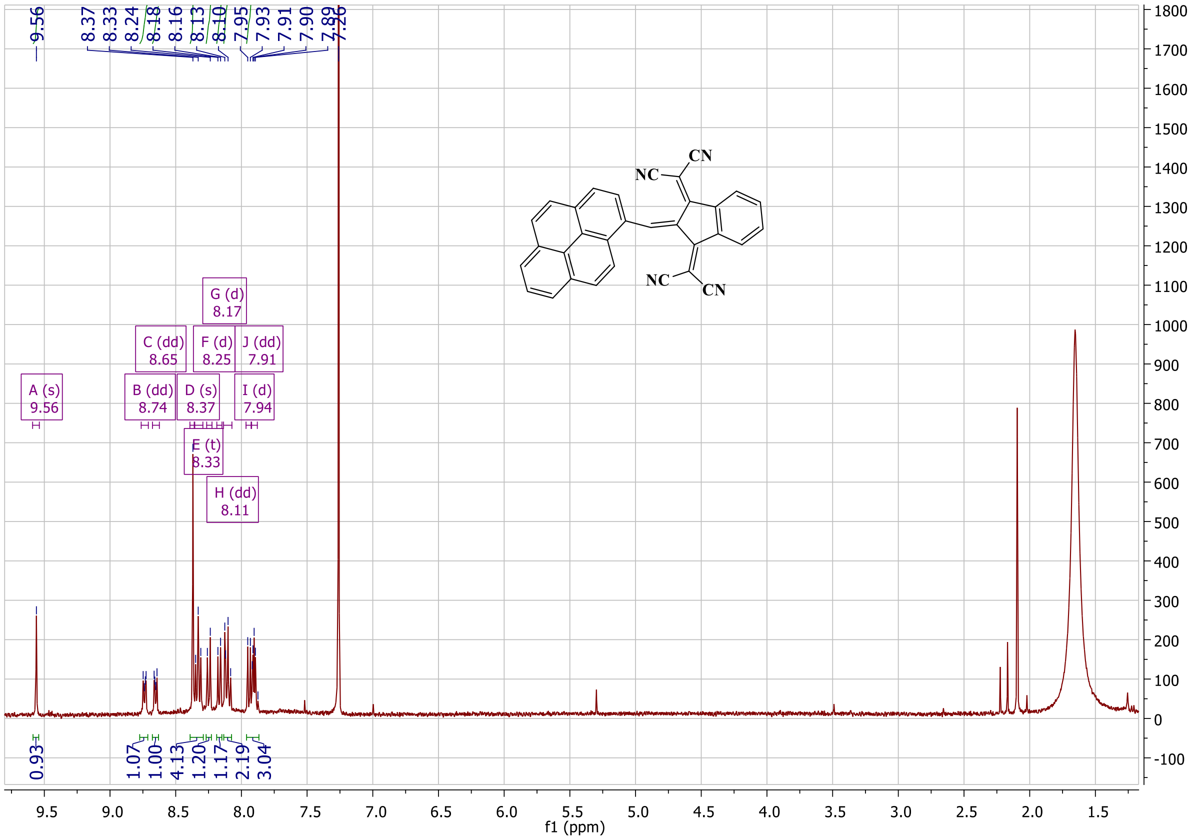

Synthesis of 2-(pyren-1-ylmethylene)-1*H*-cyclopenta[*b*]naphthalene-1,3(2*H*)-dione **Dye 6**

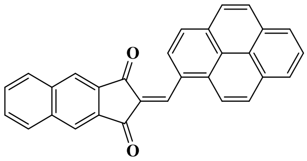

Chemical Formula: $C_{30}H_{16}O_2$
Molecular Weight: 408.4560

1-Pyrenecarboxaldehyde (1.18 g, 5.15 mmol, M = 230.27 g/mol) and 1*H*-cyclopenta[*b*]naphthalene-1,3(2*H*)-dione (1.01 g, 5.15 mmol, M = 196.20 g/mol) were suspended in 20 mL absolute ethanol and a few drops of piperidine were added. Immediately, the solution turned deep red. The flask was introduced in an oil bath preheated at 90°C. After 15 min, the reaction was ended (TLC control). During that time, an extremely insoluble precipitate formed. After cooling, the precipitate was filtered off, washed several times with ethanol and ether. Due to its insolubility, no ^{1}H NMR spectrum could be acquired (1.60 g, 76% yield). HRMS (ESI MS) m/z: theor: 408.1150 found: 408.1157 ($[M]^{+\cdot}$ detected); Anal. Calc. for $C_{30}H_{16}O_2$: C, 88.2; H, 3.9; O, 7.8; Found: C, 88.4; H, 3.9; N, 8.1 %

Synthesis of 2-(3-oxo-2-(pyren-1-ylmethylene)-2,3-dihydro-1*H*-cyclopenta[*b*]naphthalen-1-ylidene)malononitrile **Dye 7**

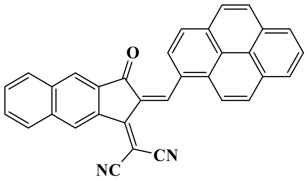

Chemical Formula: $C_{33}H_{16}N_2O$
Molecular Weight: 456.5040

1-Pyrenecarboxaldehyde (1.18 g, 5.15 mmol, M = 230.27 g/mol) and 2-(3-oxo-2,3-dihydro-1*H*-cyclopenta[*b*]naphthalen-1-ylidene)malononitrile (1.26 g, 5.15 mmol, M = 244.25 g/mol) were suspended in 20 mL absolute ethanol and a few drops of piperidine were added. Immediately, the solution turned deep red. The flask was introduced in an oil bath preheated at 90°C. After 15 min, the reaction was ended (TLC control). During that time, an extremely insoluble precipitate formed. After cooling, the precipitate was filtered off, washed several times with ethanol and ether (1.90 g, 81% yield). Due to its insolubility, no ^{1}H NMR spectrum could be acquired. HRMS (ESI MS) m/z: theor: 456.1263 found: 456.1256 ($[M]^{+\cdot}$ detected); Anal. Calc. for $C_{33}H_{16}N_2O$: C, 86.8; H, 3.5; O, 3.5; Found: C, 86.8; H, 3.6; N, 3.6 %

Synthesis of 5-(pyren-1-ylmethylene)pyrimidine-2,4,6(1*H*,3*H*,5*H*)-trione **Dye 8**

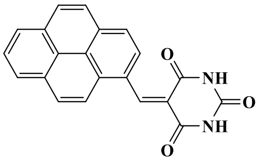

Chemical Formula: $C_{21}H_{12}N_2O_3$
Molecular Weight: 340.3380

1-Pyrenecarbaldehyde (2 g, 8.68 mmol, M = 230.26 g/mol), barbituric acid (1.11 g, 8.68 mmol, M = 128.09 g/mol) were dissolved in absolute ethanol (100 mL). Immediately, the solution turned orange. The solution was refluxed for 4 hours. After cooling, the precipitate was filtered off, washed several times with ether and dried under vacuum (2.45 g, 83% yield). ^{1}H NMR (400 MHz, DMSO) δ 11.50 (s, 1H), 11.21 (s, 1H), 9.11 (s, 1H), 8.42 – 8.11 (m, 9H); HRMS (ESI MS) m/z: theor: 340.0848 found: 340.0842 ($[M]^{+\cdot}$ detected); Anal. Calc. for $C_{21}H_{12}N_2O_3$: C, 74.1; H, 3.5; O, 14.1; Found: C, 74.4; H, 3.7; N, 14.2 %

Figure S7. ^{1}H NMR spectrum of **Dye 8**

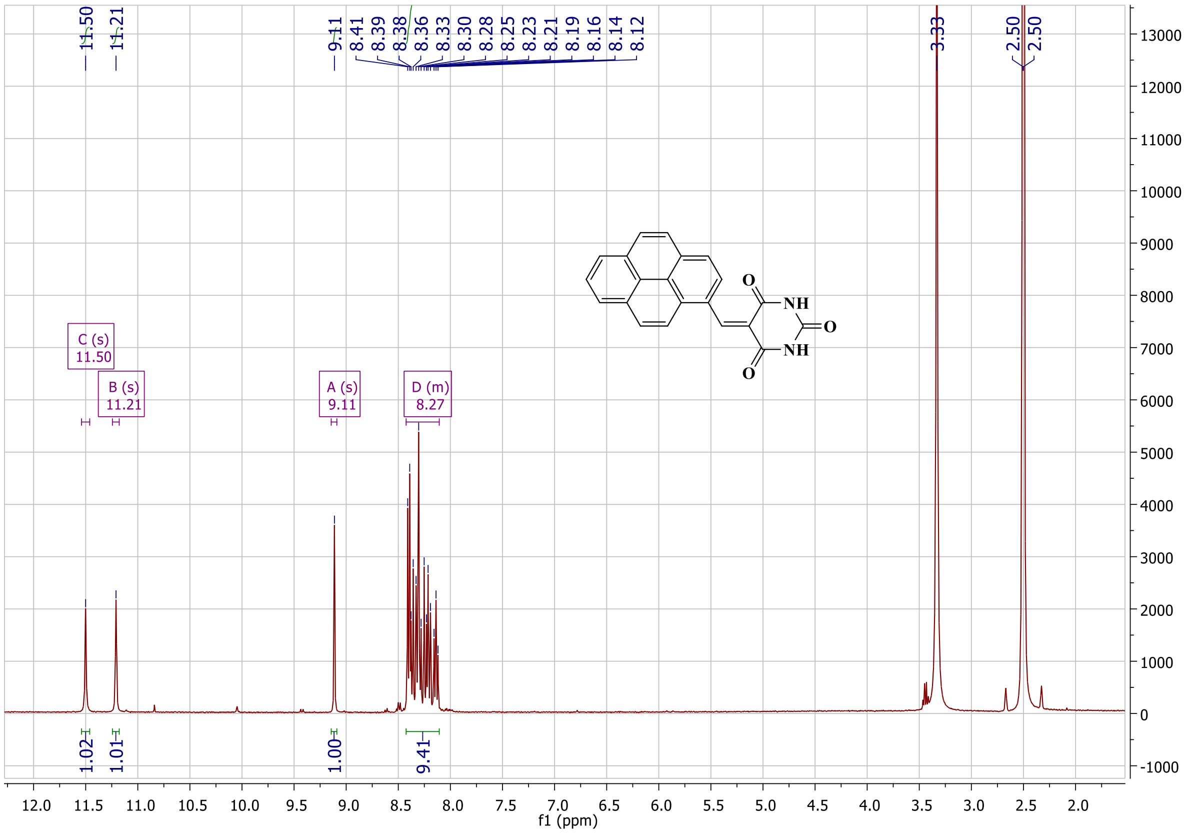

Synthesis of 5-(pyren-1-ylmethylene)-2-thioxodihydropyrimidine-4,6(1*H*,5*H*)-dione **Dye 9**

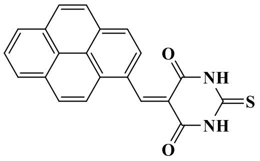

Chemical Formula: $C_{21}H_{12}N_2O_2S$
Molecular Weight: 356.3990

1-Pyrenecarbaldehyde (2 g, 8.68 mmol, M = 230.26 g/mol), thiobarbituric acid (1.25 g, 8.68 mmol, M = 144.15 g/mol) were dissolved in absolute ethanol (200 mL). Immediately, the solution turned purple. The solution was refluxed for 4 hours. After cooling, the precipitate was filtered off, washed several times with ether and dried under vacuum (2.60, 84% yield). ^{1}H NMR (400 MHz, DMSO) δ 12.57 (s, 1H), 12.33 (s, 1H), 9.14 (s, 1H), 8.42 (dd, J = 12.8, 7.9 Hz, 3H), 8.31 (dd, J = 15.2, 8.6 Hz, 3H), 8.23 (dd, J = 9.0, 3.4 Hz, 2H), 8.14 (t, J = 7.6 Hz, 1H);

HRMS (ESI MS) m/z: theor: 356.0619 found: 356.0619 ([M]$^{+\cdot}$ detected); Anal. Calc. for $C_{21}H_{12}N_2O_2S$: C, 70.8; H, 3.4; O, 9.0; Found: C, 70.6; H, 3.3; N, 9.2 %

Figure S8. ^{1}H NMR spectrum of **Dye 9**

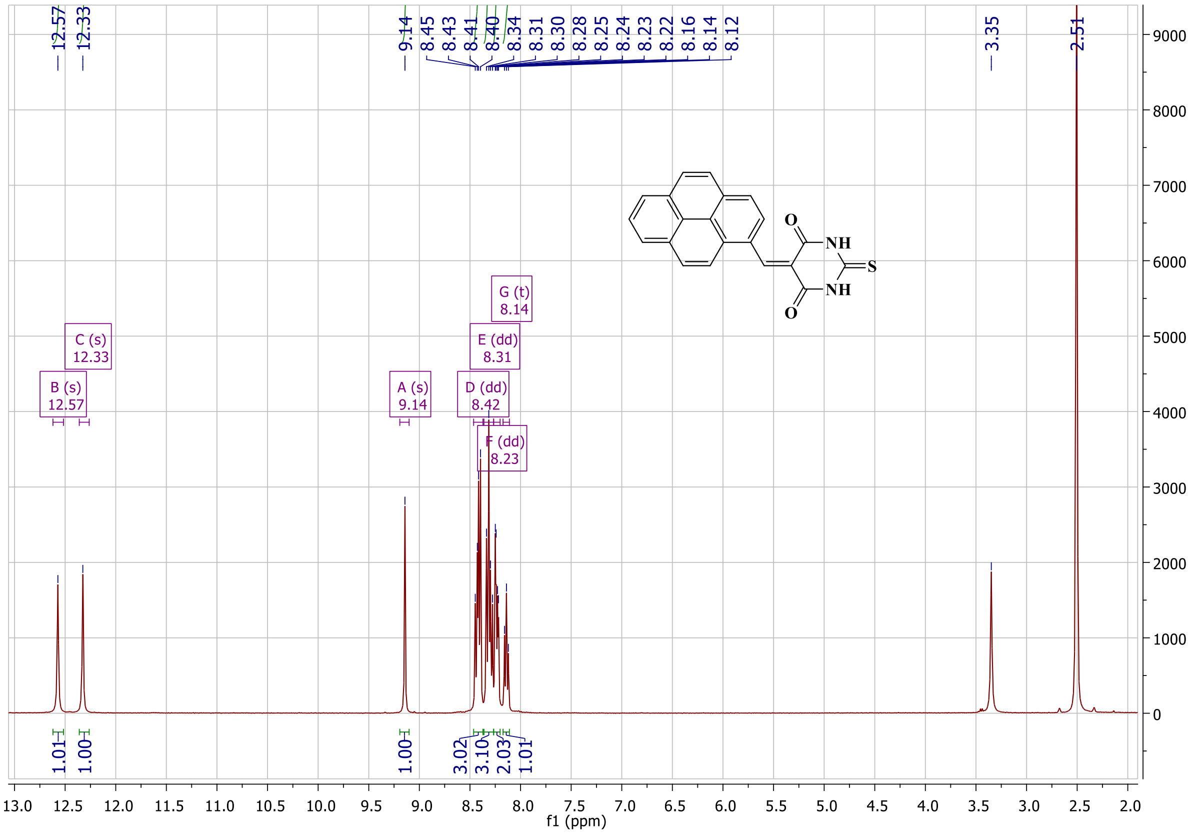

Synthesis of 1,3-dimethyl-5-(pyren-1-ylmethylene)pyrimidine-2,4,6(1*H*,3*H*,5*H*)-trione **Dye 10**

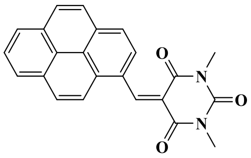

Chemical Formula: $C_{23}H_{16}N_2O_3$
Molecular Weight: 368.3920

Pyrene-1-carbaldehyde (1 g, 4.34 mmol, M = 230.27 g/mol) and 1,3-dimethylpyrimidine-2,4,6(1*H*,3*H*,5*H*)-trione (0.68 g, 4.34 mmol, M = 156.14 g/mol) were suspended in ethanol (50 mL) and a few drops of piperidine were added. The solution was refluxed overnight. Upon cooling, a precipitate formed. it was filtered off, washed several times with ether and pentane, and dried under vacuum (1.25 g, 78% yield). ^{1}H NMR (400 MHz, $CDCl_3$) δ 9.56 (s, 1H), 8.47 (d, *J* = 8.2 Hz, 1H), 8.26 (d, *J* = 7.6 Hz, 2H), 8.21 – 8.14 (m, 4H), 8.11 – 8.01 (m, 2H), 3.51 (s, 3H), 3.35 (s, 3H); ^{13}C NMR (101 MHz, $CDCl_3$) δ 162.44, 160.05, 157.60, 151.47, 134.27, 131.08, 131.04, 130.56, 129.65, 129.32, 128.75, 127.38, 127.22, 126.75, 126.66, 126.36, 124.37, 123.75, 123.34, 118.56, 29.03, 28.40; HRMS (ESI MS) m/z: theor: 368.1161 found: 368.1165 ([M]$^{+\cdot}$ detected)

Figure S9. ¹H NMR spectrum of **Dye 10**

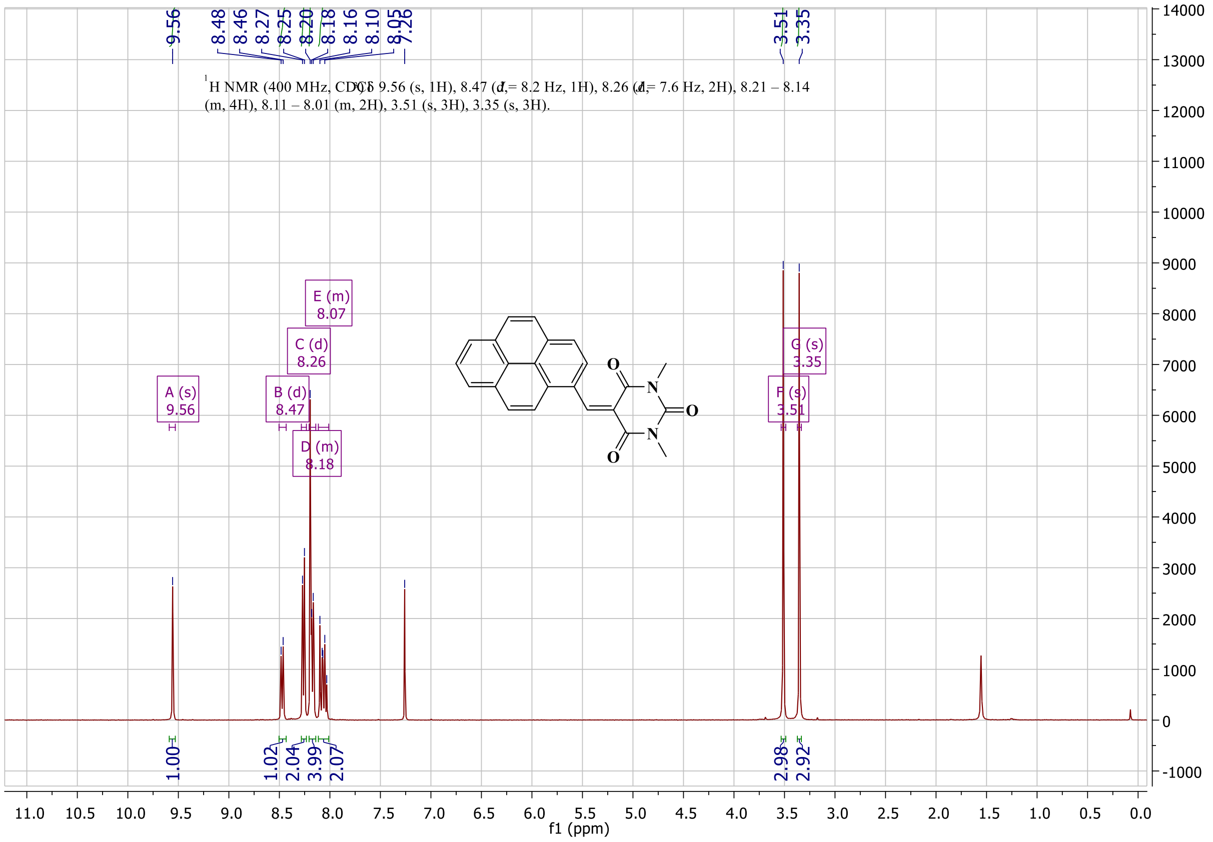

Figure S10. ¹³C NMR spectrum of **Dye 10**

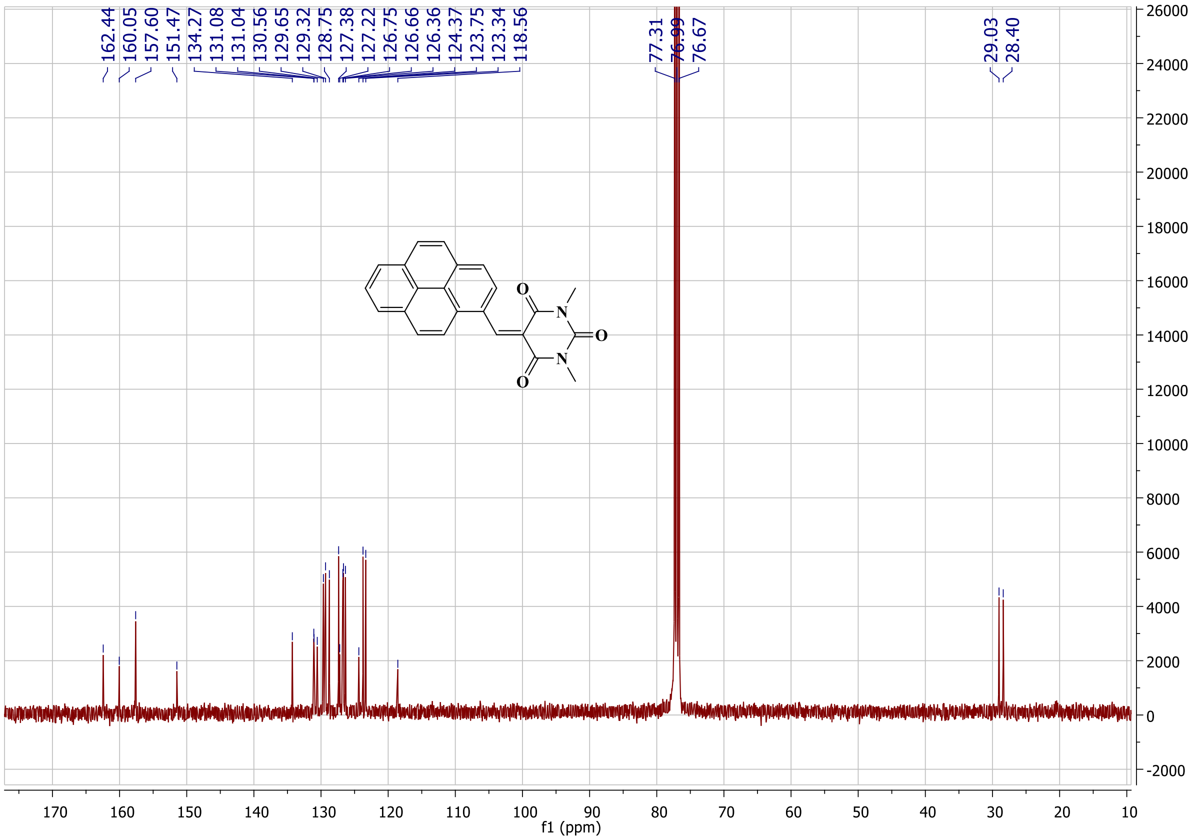

Synthesis of 1,3-diethyl-5-(pyren-1-ylmethylene)-2-thioxodihydropyrimidine-4,6(1*H*,5*H*)-dione **Dye 11**

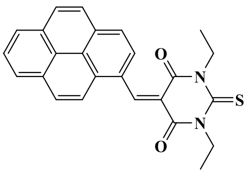

Chemical Formula: $C_{25}H_{20}N_2O_2S$
Molecular Weight: 412.5070

Pyrene-1-carbaldehyde (1 g, 4.34 mmol, M = 230.27 g/mol) and 1,3-diethyl-2-thioxodihydropyrimidine-4,6(1*H*,5*H*)-dione (0.87 g, 4.34 mmol, M = 200.26 g/mol) were suspended in ethanol (50 mL) and a few drops of piperidine were added. The solution was refluxed overnight. Upon cooling, a precipitate formed. it was filtered off, washed several times with ether and pentane, and dried under vacuum (1.52 g, 85% yield). ^{1}H NMR (400 MHz, $CDCl_3$) δ 9.57 (s, 1H), 8.55 (d, *J* = 8.2 Hz, 1H), 8.27 (dd, *J* = 15.9, 6.5 Hz, 4H), 8.20 (t, *J* = 7.7 Hz, 2H), 8.13 – 8.04 (m, 2H), 4.66 (q, *J* = 6.9 Hz, 2H), 4.53 (q, *J* = 6.9 Hz, 2H), 1.41 (t, *J* = 7.0 Hz, 3H), 1.29 (t, *J* = 7.0 Hz, 3H); ^{13}C NMR (75 MHz, $CDCl_3$) δ 179.12, 160.73, 158.29, 158.14, 134.65, 131.44, 131.04, 130.52, 129.93, 129.47, 129.03, 127.39, 127.32, 126.93, 126.84, 126.41, 124.32, 124.30, 123.81, 123.36, 118.88, 44.15, 43.62, 12.53, 12.47; HRMS (ESI MS) m/z: theor: 412.1245 found: 412.1242 ($[M]^{+\cdot}$ detected)

Figure S11. ^{1}H NMR spectrum of **Dye 11**

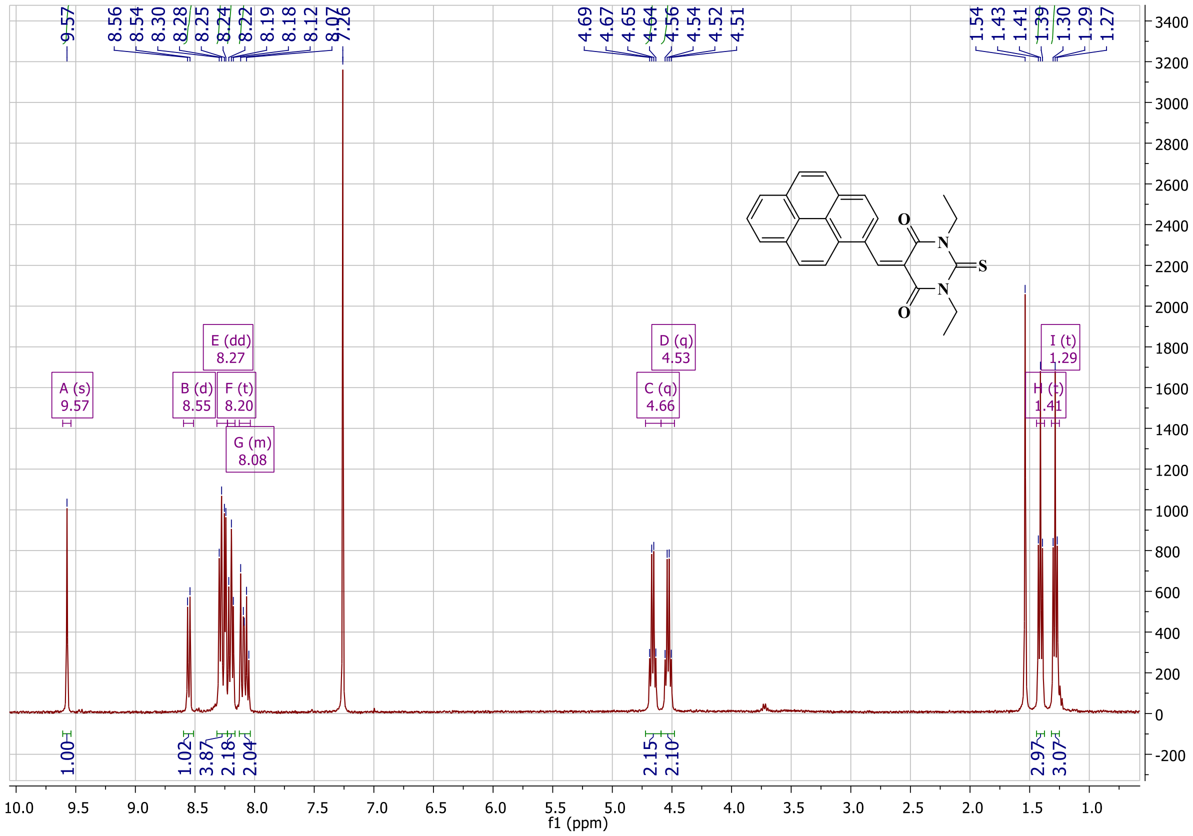

Figure S12. ^{13}C NMR spectrum of **Dye 11**

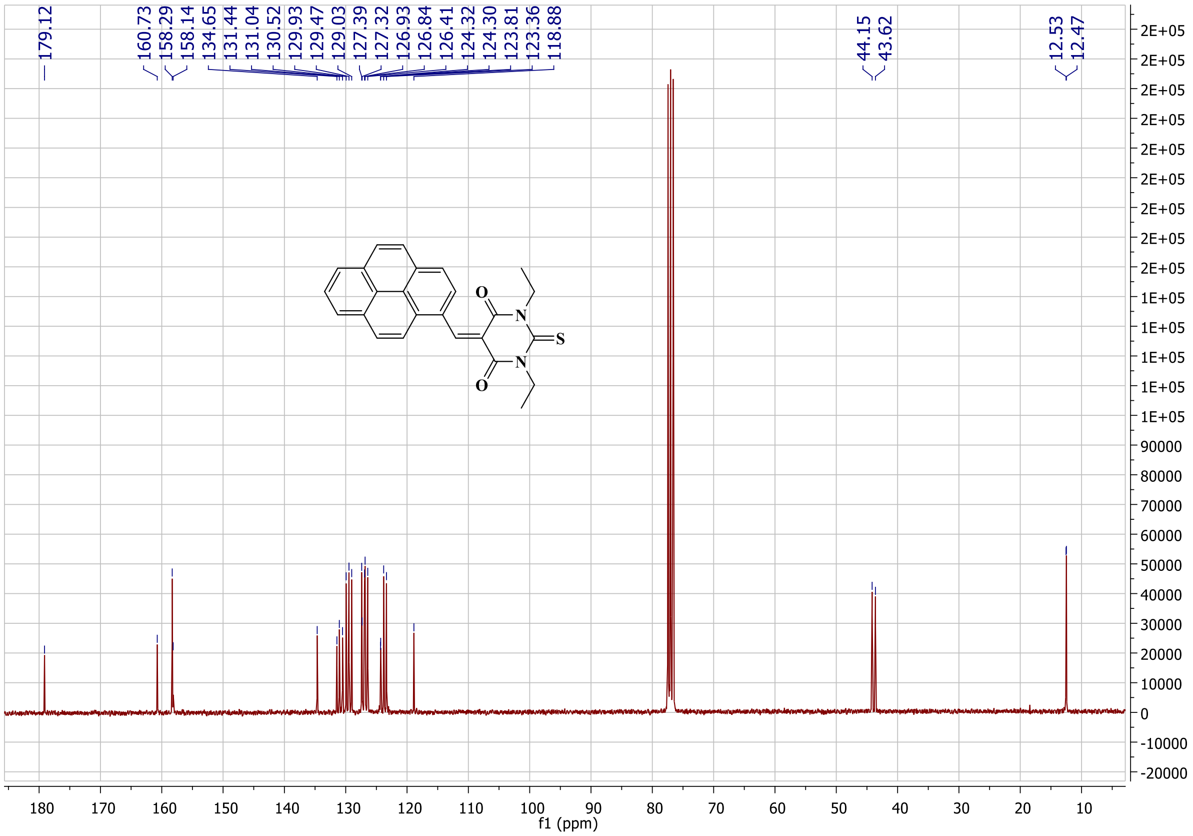

Synthesis of 3-ethyl-5-(pyren-1-ylmethylene)-2-thioxothiazolidin-4-one **Dye 12**

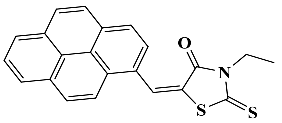

Chemical Formula: $C_{22}H_{15}NOS_2$
Molecular Weight: 373.4880

Pyrene-1-carbaldehyde (1 g, 4.34 mmol, M = 230.27 g/mol) and 3-ethylrhodanine (0.70 g, 4.34 mmol, M = 161.25 g/mol) were suspended in ethanol (50 mL) and a few drops of piperidine were added. The solution was refluxed overnight. Upon cooling, a precipitate formed. it was filtered off, washed several times with ether and pentane, and dried under vacuum (1.30 g, 80% yield). ^{1}H NMR (300 MHz, $CDCl_3$) δ 8.76 (s, 1H), 8.42 (d, J = 9.3 Hz, 1H), 8.28 – 8.11 (m, 5H), 8.05 (t, J = 6.9 Hz, 3H), 4.27 (q, J = 7.1 Hz, 2H), 1.36 (t, J = 7.1 Hz, 3H); ^{13}C NMR (75 MHz, $CDCl_3$) δ 193.66, 167.40, 133.03, 131.27, 130.97, 130.66, 130.01, 129.53, 129.24, 127.25, 126.91, 126.60, 126.56, 126.43, 125.66, 125.09, 125.01, 124.91, 124.34, 122.35, 39.84, 12.32; HRMS (ESI MS) m/z: theor: 373.05955 found: 373.0590 ($[M]^{+\cdot}$ detected)

Figure S13. ^{1}H NMR spectrum of **Dye 12**

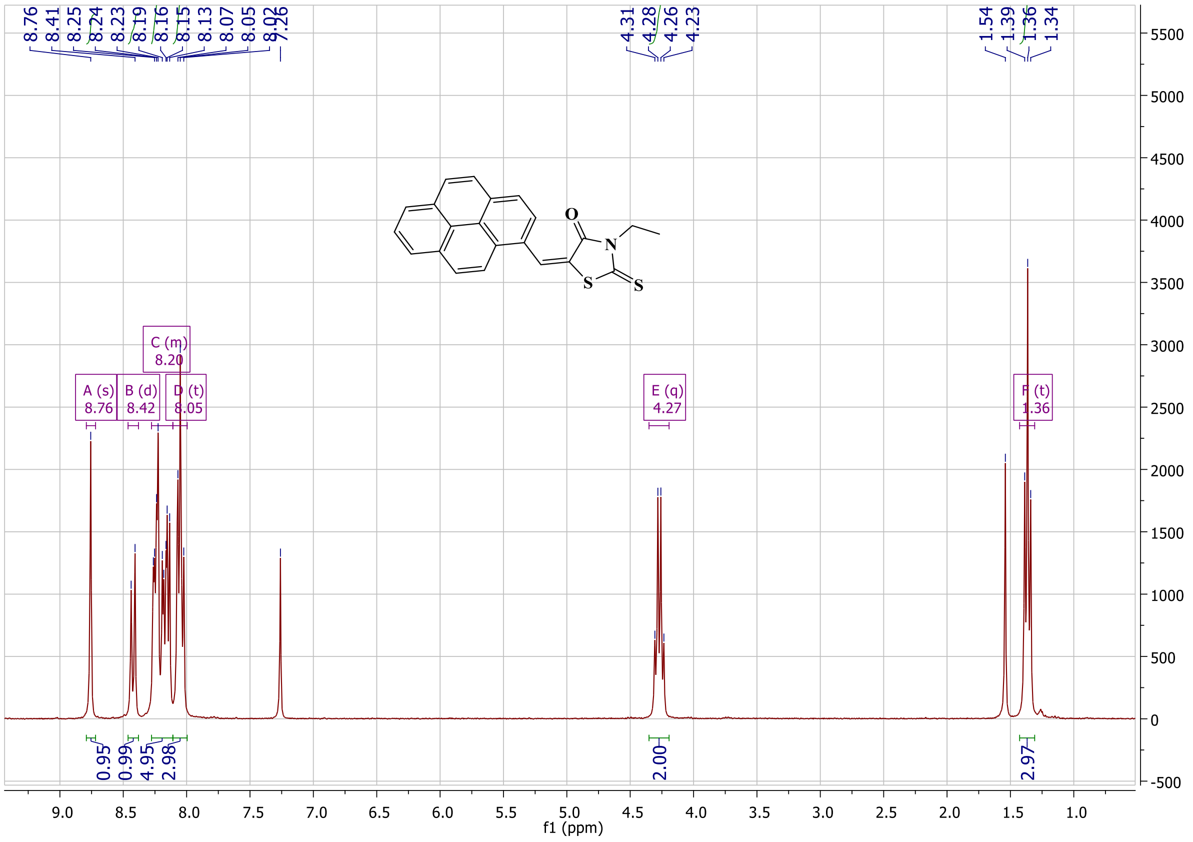

Figure S14. ^{13}C NMR spectrum of **Dye 12**

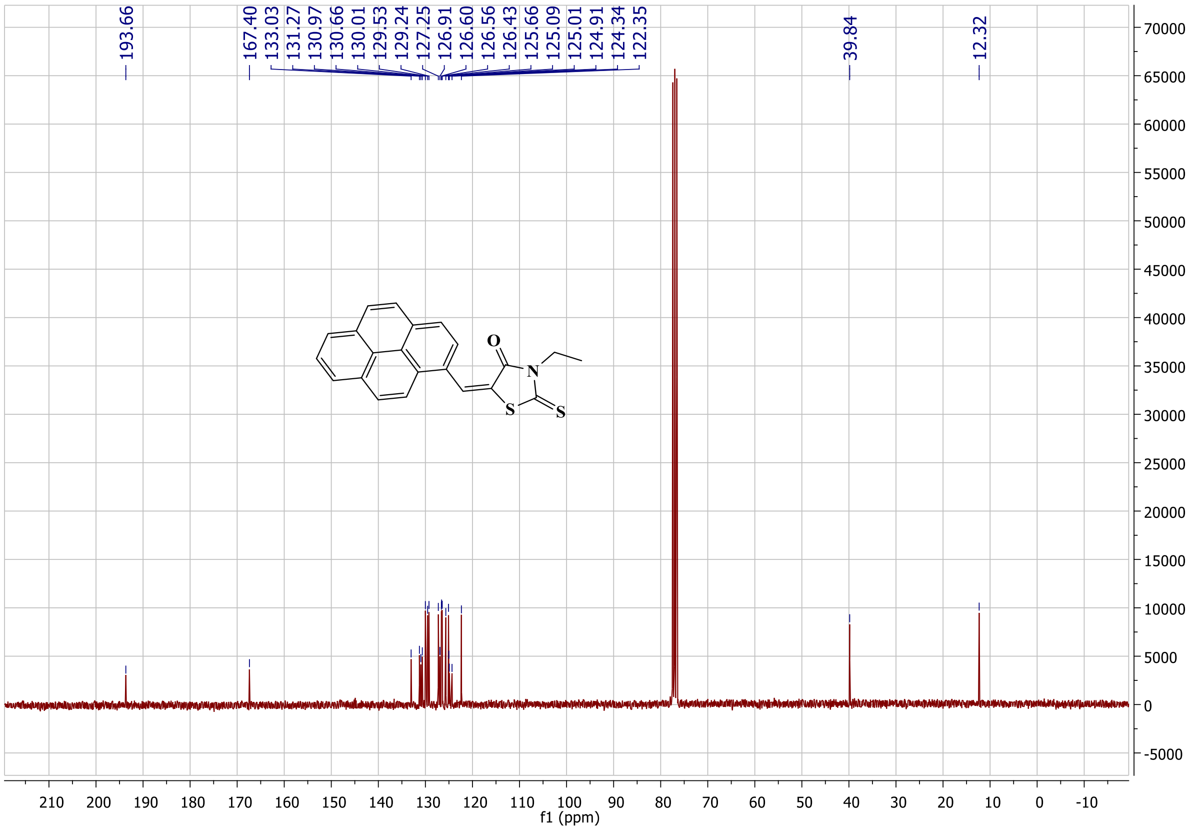

Synthesis of 3-allyl-5-(pyren-1-ylmethylene)-2-thioxothiazolidin-4-one **Dye 13**

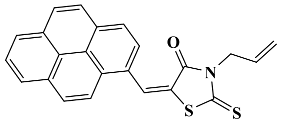

Chemical Formula: $C_{23}H_{15}NOS_2$
Molecular Weight: 385.4990

Pyrene-1-carbaldehyde (1 g, 4.34 mmol, M = 230.27 g/mol) and 3-allylrhodanine (0.75 g, 4.34 mmol, M = 173.26 g/mol) were suspended in ethanol (50 mL) and a few drops of piperidine were added. The solution was refluxed overnight. Upon cooling, a precipitate formed. it was filtered off, washed several times with ether and pentane, and dried under vacuum (1.37 g, 82% yield). ^{1}H NMR (400 MHz, $CDCl_3$) δ 8.81 (s, 1H), 8.46 (d, *J* = 9.3 Hz, 1H), 8.29 – 8.04 (m, 8H), 5.99 – 5.88 (m, 1H), 5.34 (ddd, *J* = 13.7, 11.4, 1.2 Hz, 2H), 4.83 (dt, *J* = 5.9, 1.3 Hz, 2H); HRMS (ESI MS) m/z: theor: 385.0595 found: 385.0598 ($[M]^{+\cdot}$ detected); Anal. Calc. for $C_{23}H_{15}NOS_2$: C, 71.7; H, 3.9; O, 4.1; Found: C, 71.4; H, 3.7; N, 4.4 %

Figure S15. ^{1}H NMR spectrum of **Dye 13**

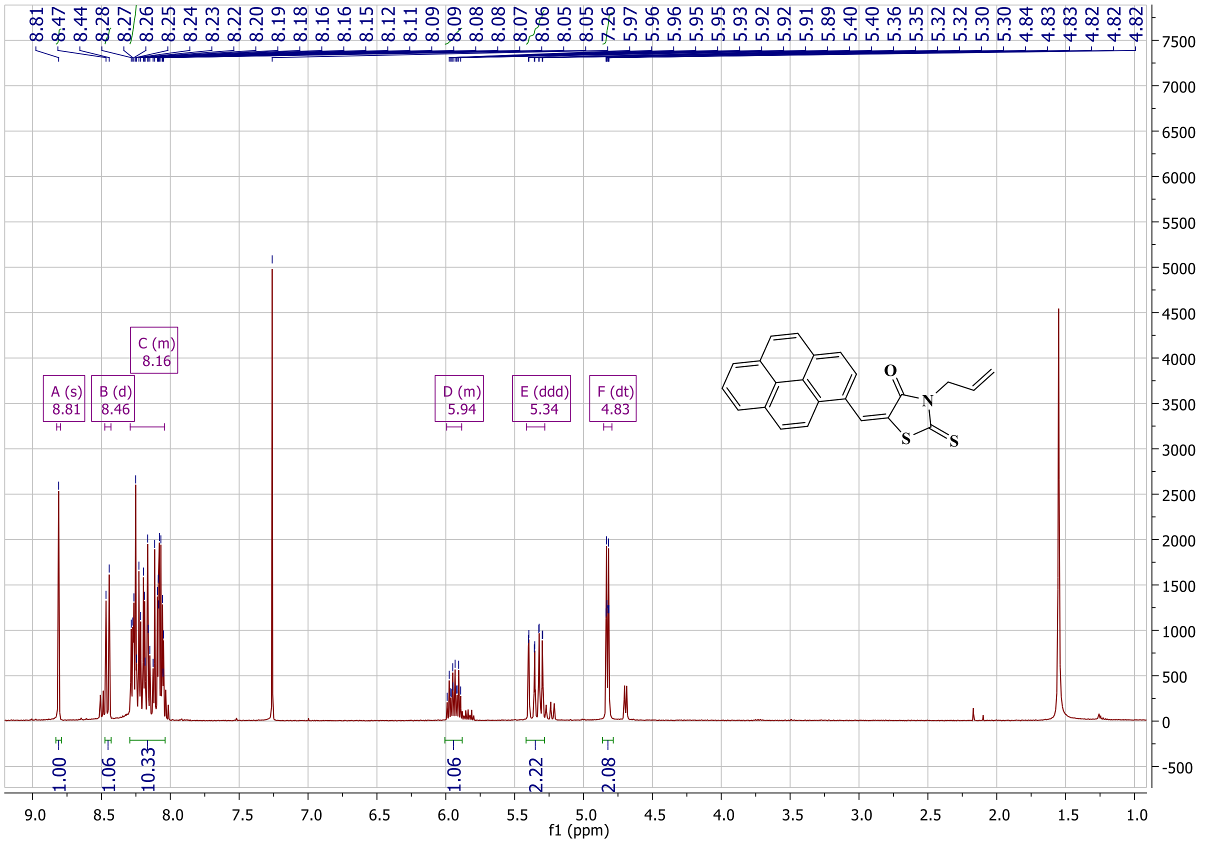

Synthesis of 2-(3-cyano-5,5-dimethyl-4-(2-(pyren-1-yl)vinyl)furan-2(5*H*)-ylidene) malononitrile **Dye 14**

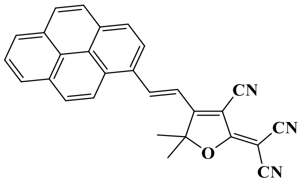

Chemical Formula: $C_{28}H_{17}N_3O$
Molecular Weight: 411.4640

2-(3-Cyano-4,5,5-trimethylfuran-2(5H)-ylidene)malononitrile (1 g, 5.02 mmol, M = 199.21 g/mol) and pyrene-1-carbaldehyde (1.16 g, 5.02 mmol, M = 230.27 g/mol)were dissolved in absolute ethanol (20 mL) and a few drops of piperidine were added. The solution was refluxed overnight. After cooling, the solvent was removed under reduced pressure. Addition of ether followed by pentane precipitated a blue solid that was filtered off, washed several times with pentane and dried under vacuum (1.69 g, 82% yield). Due to its insolubility, no ^{1}H NMR spectrum could be acquired. HRMS (ESI MS) m/z: theor: 411.1372 found: 411.1373 ($[M]^{+\cdot}$ detected); Anal. Calc. for $C_{28}H_{17}N_3O$: C, 81.7; H, 4.2; O, 3.9; Found: C, 81.4; H, 3.9; N, 4.1 %

Synthesis of 2-(pyren-1-ylmethylene)-[1,2'-biindenylidene]-1',3,3'(2*H*)-trione **Dye 15**

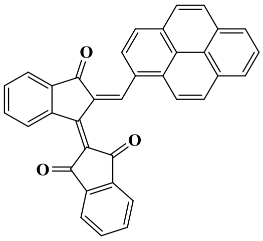

Chemical Formula: $C_{35}H_{18}O_3$
Molecular Weight: 486.5260

To a mixture of [1,2']-biindenylidene-3,1',3'-trione (0.5 g, 1.28 mmol, M = 274.28 g/mol) and 2-pyrenecarbaldehyde (0.42 g, 1.28 mmol, 230.27 g/mol) was added acetic anhydride so that the powder was covered by the liquid. The reaction mixture was heated at 90°C overnight. After cooling, ether was added. A red precipitate formed. It was filtered off, washed several times with ether and dried under vacuum to remove the traces of acetic anhydride (0.55 g, 88% yield). ^{1}H NMR (400 MHz, $CDCl_3$) δ 9.39 (d, *J* = 7.5 Hz, 1H), 9.30 (dd, *J* = 17.0, 7.9 Hz, 2H), 8.05 (d, *J* = 7.4 Hz, 1H), 7.89 – 7.79 (m, 3H), 7.75 (dt, *J* = 19.1, 6.1 Hz, 3H), 7.68 (d, *J* = 7.7 Hz, 1H), 7.61 (t, *J* = 7.1 Hz, 1H), 7.58 – 7.49 (m, 2H), 7.39 (t, *J* = 7.4 Hz, 1H), 7.28 (s, 3H); HRMS (ESI MS) m/z: theor: 486.1256 found: 486.1251 ($[M]^{+\cdot}$ detected); Anal. Calc. for $C_{35}H_{18}O_3$: C, 86.4; H, 3.7; O, 9.9; Found: C, 86.4; H, 3.9; N, 10.1 %

Figure S16. ^{1}H NMR spectrum of **Dye 15**

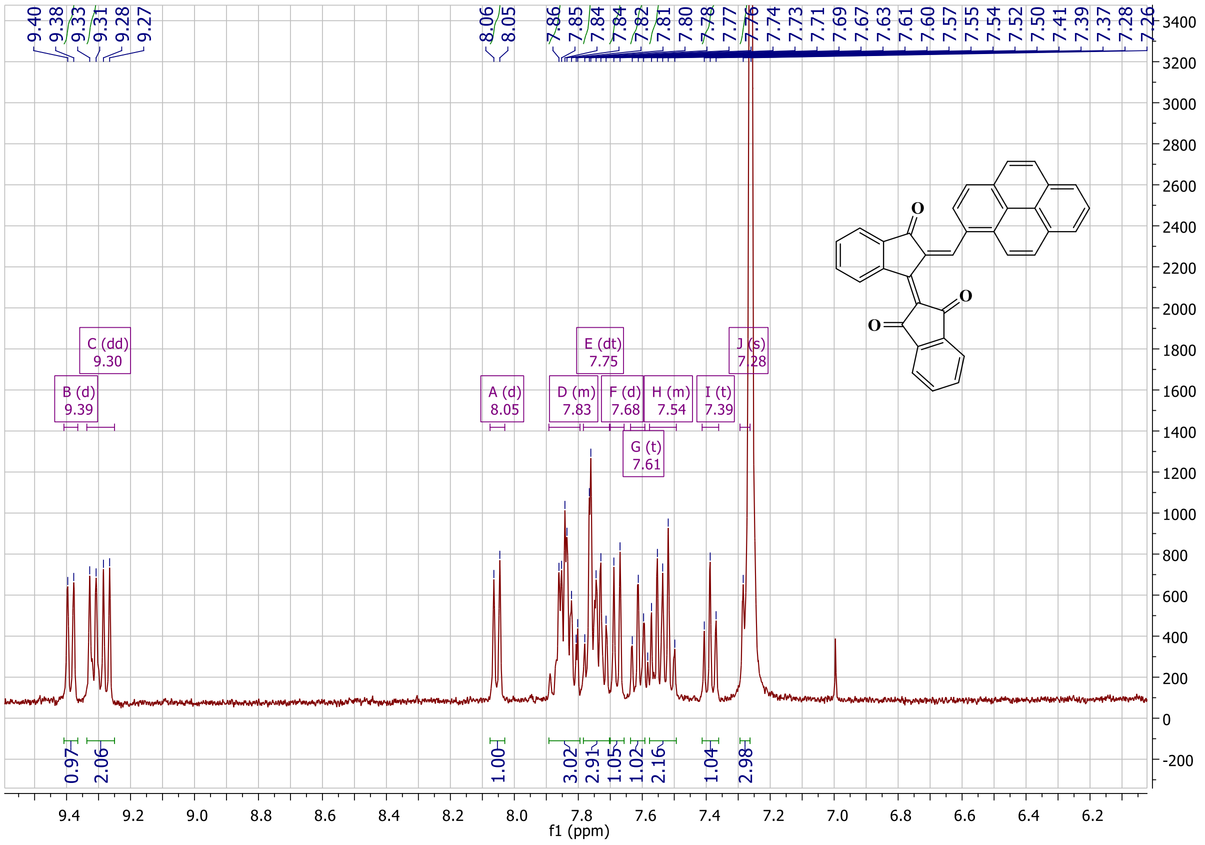

Synthesis of 2-(3-Oxo-2,3-dihydro-1*H*-cyclopenta[*b*]naphthalen-1-ylidene)malononitrile **EA7**

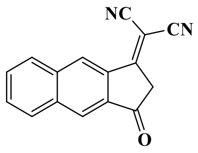

Chemical Formula: $C_{16}H_8N_2O$
Molecular Weight: 244.2530

In a dried two-necked 100 mL flask, 1H-cyclopenta[*b*]naphthalene-1,3(2*H*)-dione (1.1 g, 5.6 mmol) and malononitrile (2.2 g, 33.3 mmol) were dissolved in ethanol (25 mL), and then anhydrous sodium acetate (1.84 g) was slowly added while stirring. After stirring for 2 h, the reaction mixture was poured into ice-water, and acidified to pH 1–2 by the addition of concentred hydrochloric acid. The resulting precipitate was collected by filtration and washed with water giving the crude product. It was finally purified with a flash chromatography (eluent : DCM). Yield = 67%. ^{1}H NMR (400 MHz, $CDCl_3$) δ: 3.85 (s, 2H), 7.79 (dd, 2H, J = 6.2 Hz, J = 3.2 Hz), 8.07-8.19 (m, 2H), 8.49 (s, 1H), 9.19 (s, 1H); ^{13}C NMR (100 MHz, $CDCl_3$) δ: 44.6, 112.3, 112.6, 125.8, 128.1, 130.5, 130.6, 130.7, 130.9, 135.8, 136.3, 136.4, 166.5, 195.3; HRMS (ESI MS) m/z: theor: 244.0637 found: 244.0640 ($[M]^{+\cdot}$ detected)

Figure S17. ^{1}H NMR spectrum in $CDCl_3$

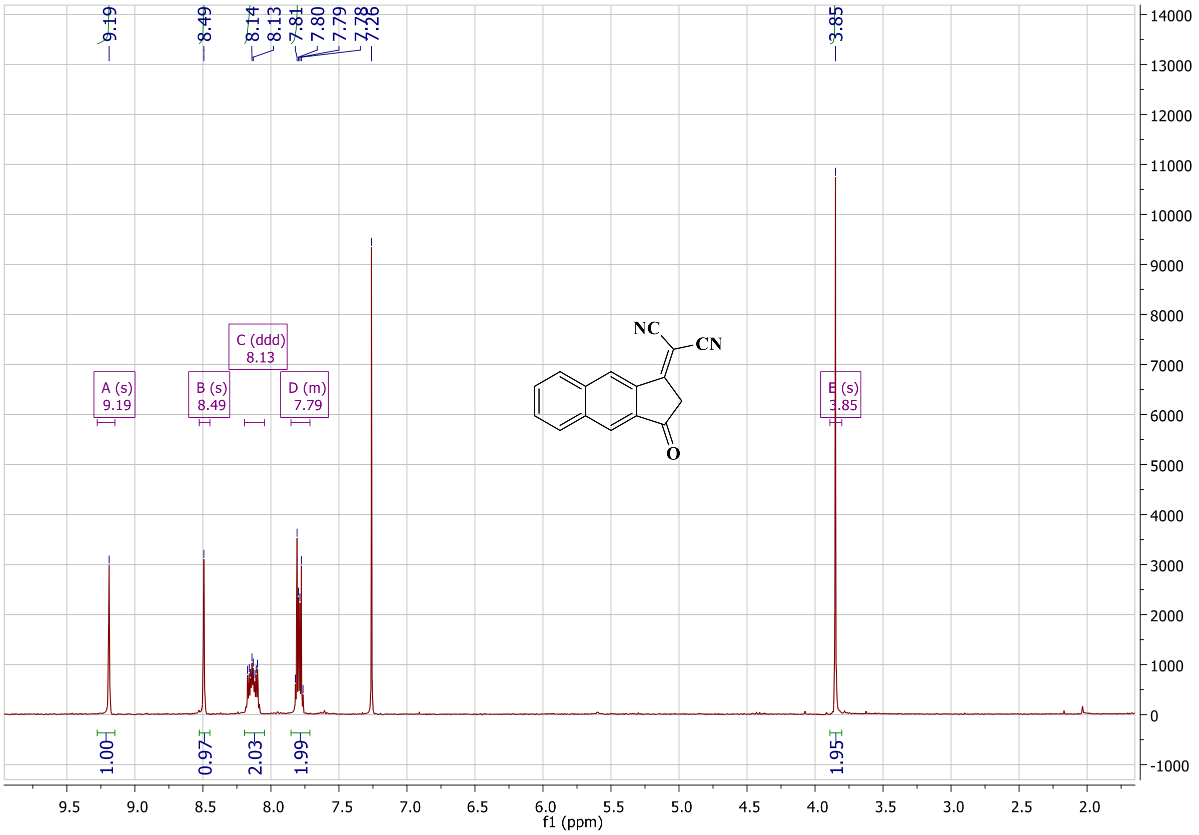

Figure S18. ^{13}C NMR spectrum in $CDCl_3$

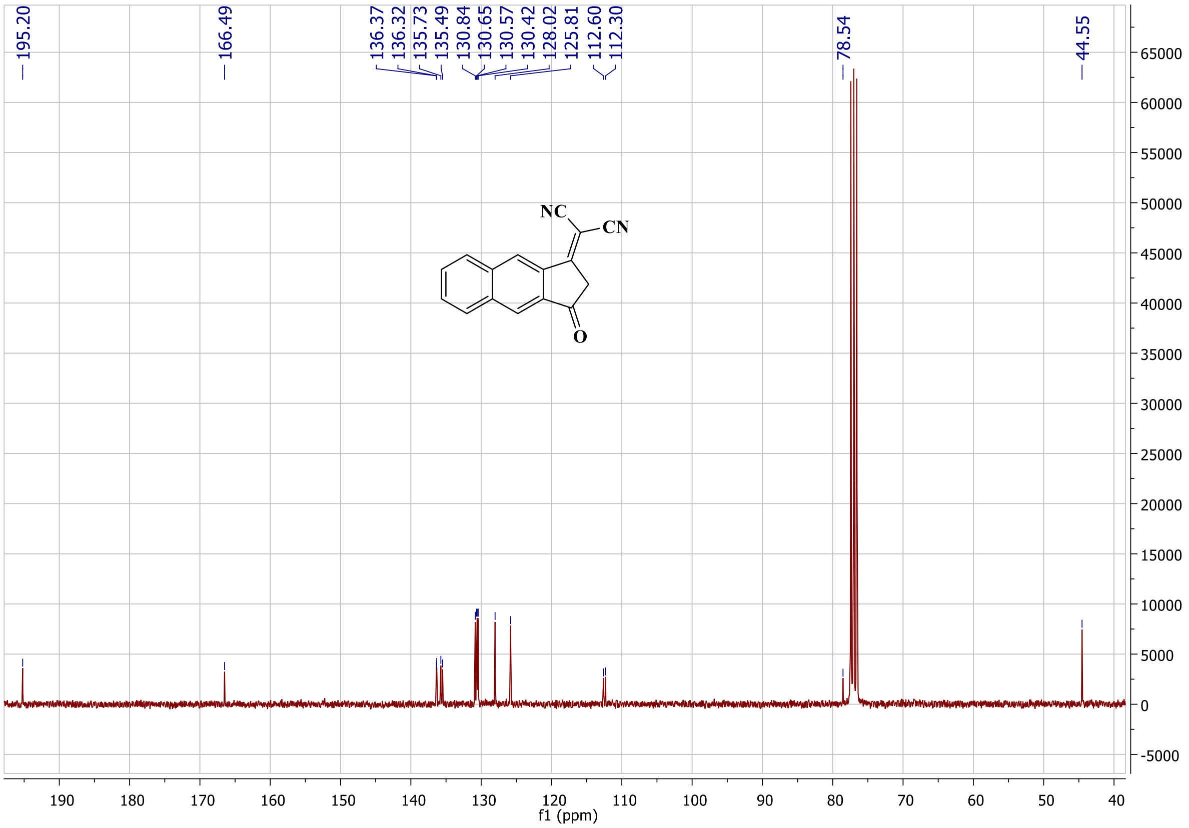

Synthesis of [1,2']biindenylidene-3,1',3'-trione **EA15**

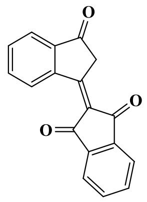

Chemical Formula: $C_{18}H_{10}O_3$
Molecular Weight: 274.2750

A solution of 1,3-indanedione (2.04 g, 14.0 mmol) in absolute ethanol (25 mL) was stirred for 5 min, before NaOAc (1.53 g, 18.6 mmol) was added. After 1 h of stirring at room temperature, the reaction mixture was diluted with ~50 mL of water. The solution was acidified with conc. HCl to a pH of ~1, the green precipitate was filtered off and dried under vacuum (1.55 g, 81% yield). ^{1}H NMR ($CDCl_3$) δ 4.17 (s, 2H), 7.73-7.77 (m, 1H), 7.81-7.88 (m, 3H), 7.94-7.98 (m, 2H), 8.01-8.03 (m, 1H), 9.68 (m, 1H, J = 8.1 Hz); ^{13}C NMR ($CDCl_3$) δ 43.4, 123.1, 123.4, 123.5, 125.9, 131.7, 134.2, 135.3, 135.3, 135.4, 140.5, 141.3, 141.7, 145.9, 155.4, 189.4, 191.1, 200.9; HRMS (ESI MS) m/z: theor: 274.0630 found: 274.0633 (M^+ detected).

Figure S19. ^{1}H NMR of [1,2']Biindenylidene-3,1',3'-trione **EA15**

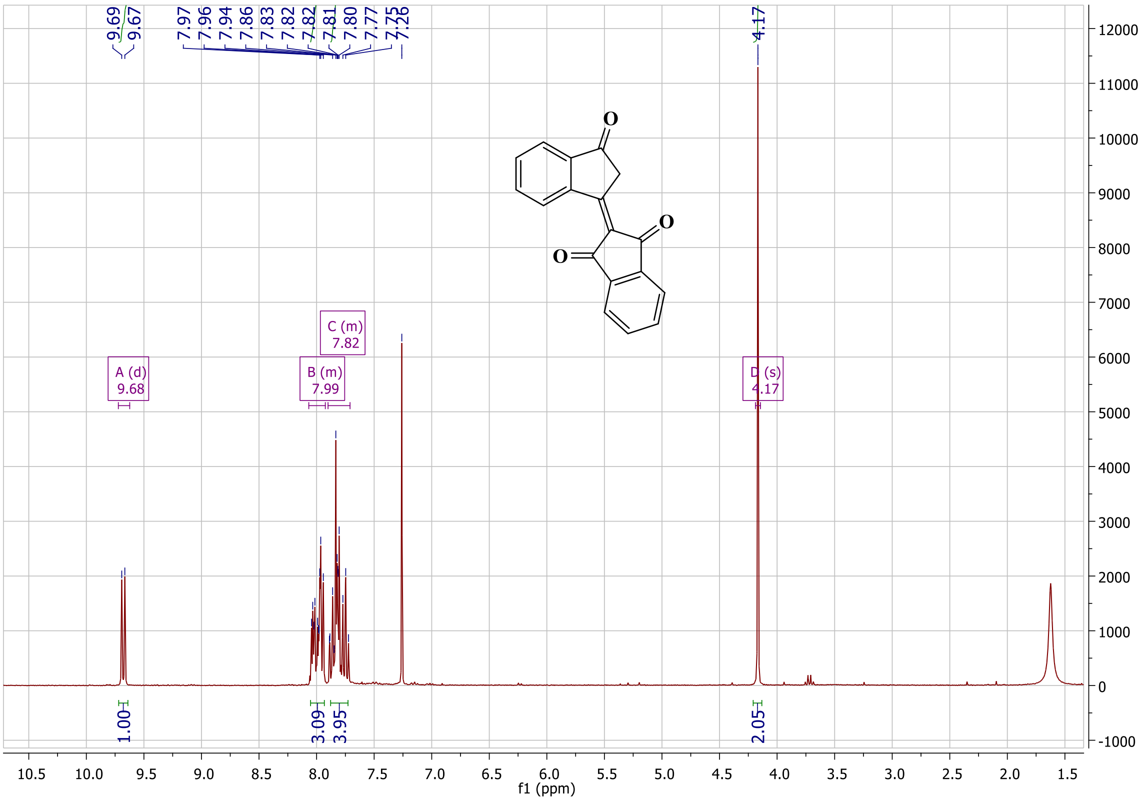

Figure S20. ^{13}C NMR of [1,2']Biindenylidene-3,1',3'-trione **EA15**

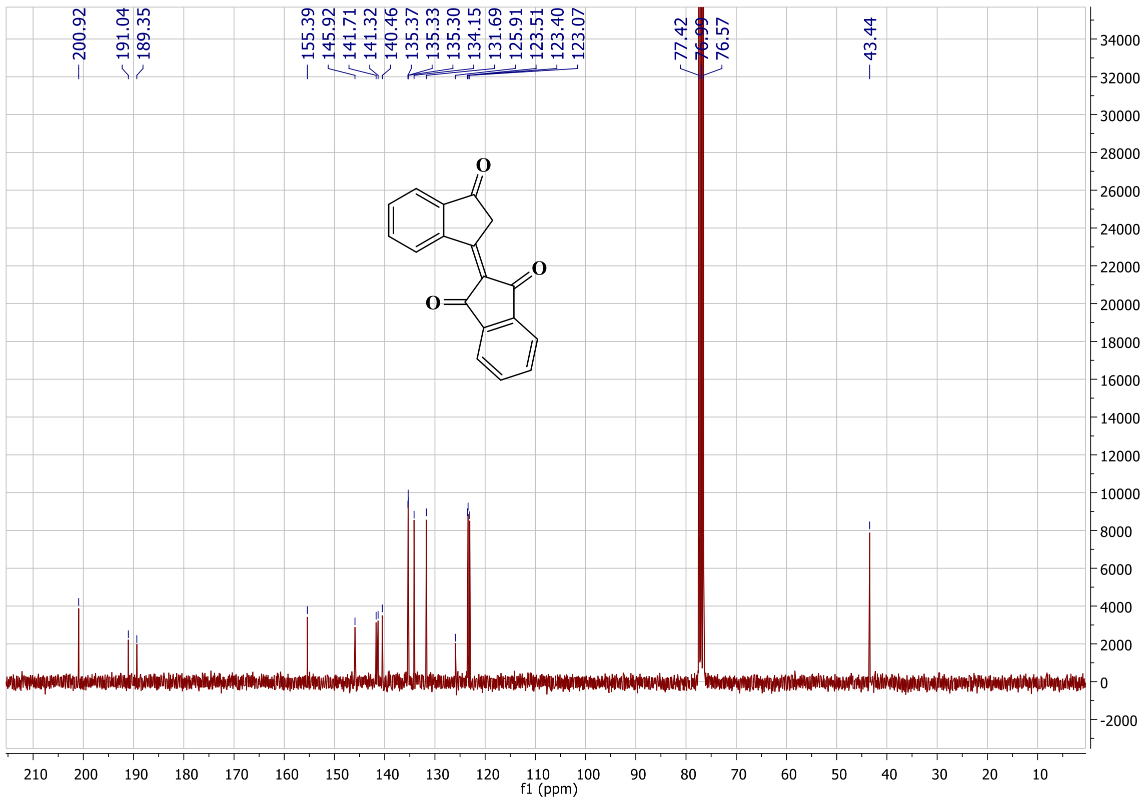

UV-visible absorption spectra in twenty-three solvents of different polarities

Figure S21. UV-visible absorption spectra of **Dye 1**.

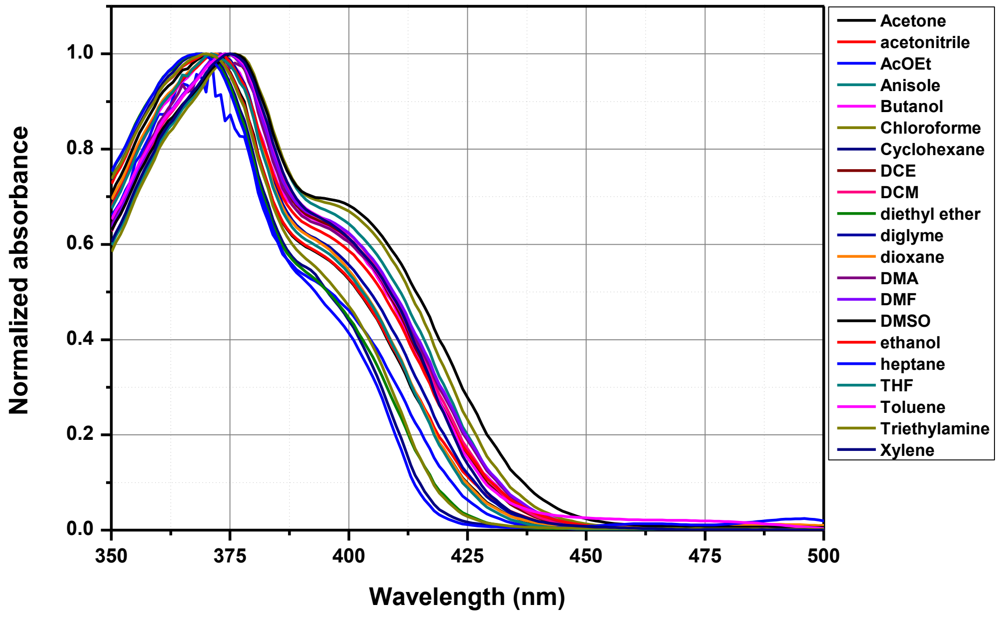

Figure S22. UV-visible absorption spectra of **Dye 2**.

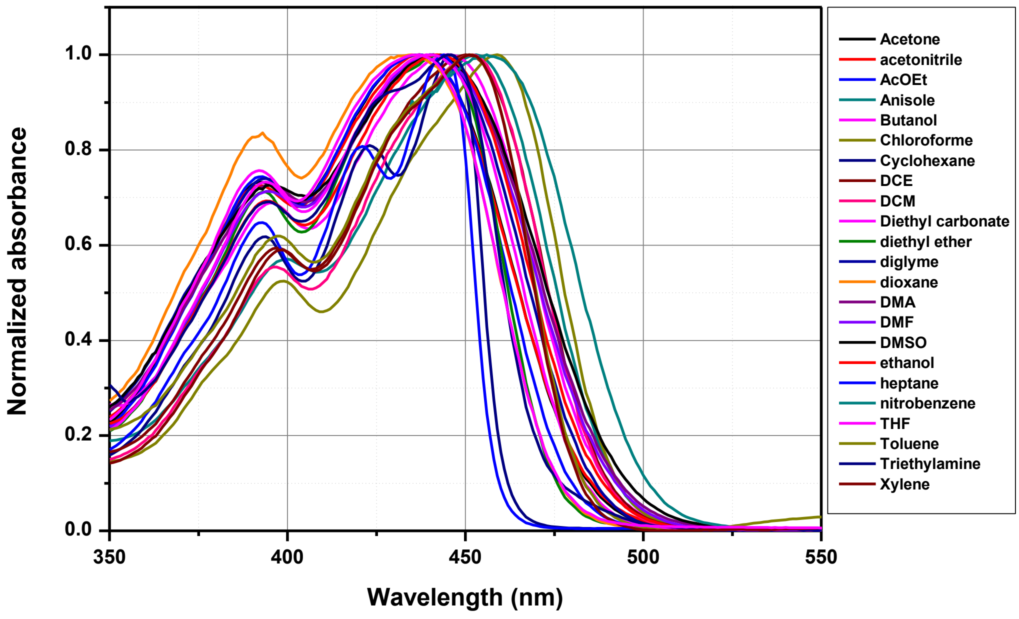

Figure S23. UV-visible absorption spectra of **Dye 3**.

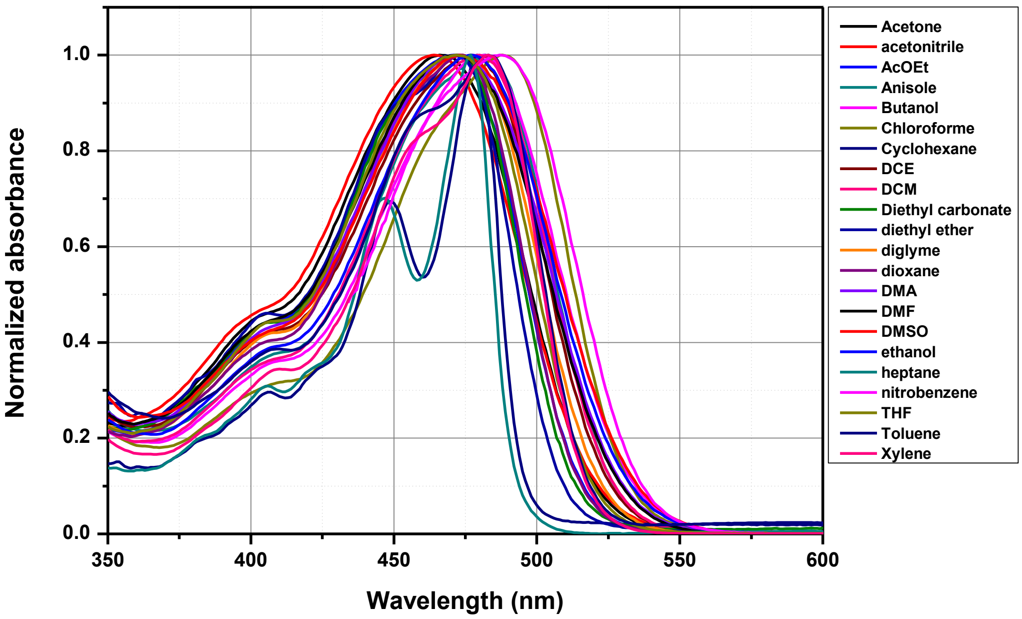

Figure S24. UV-visible absorption spectra of **Dye 4**.

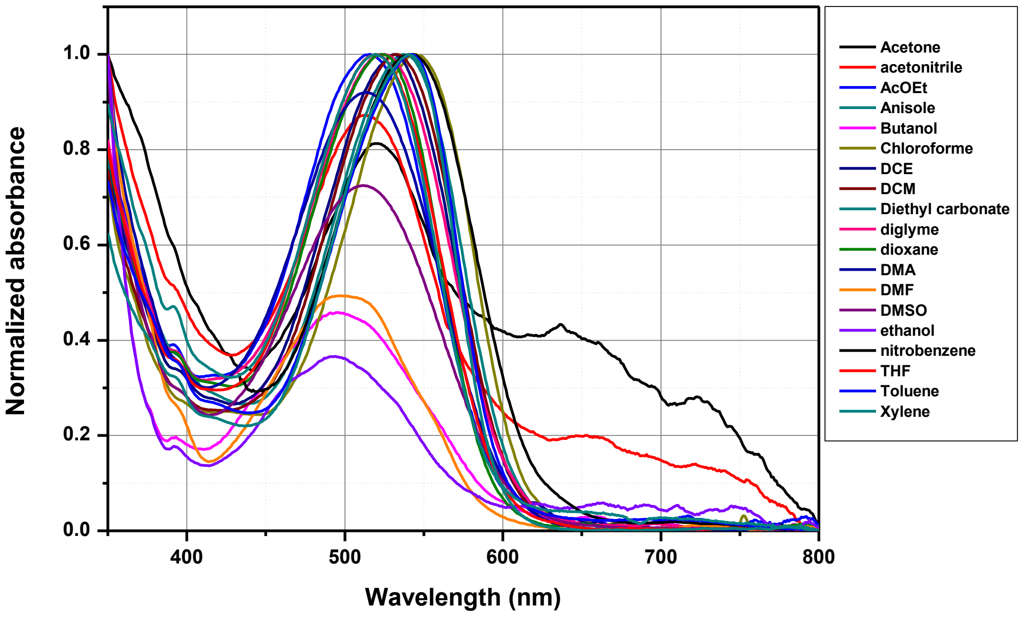

Figure S25. UV-visible absorption spectra of **Dye 5**.

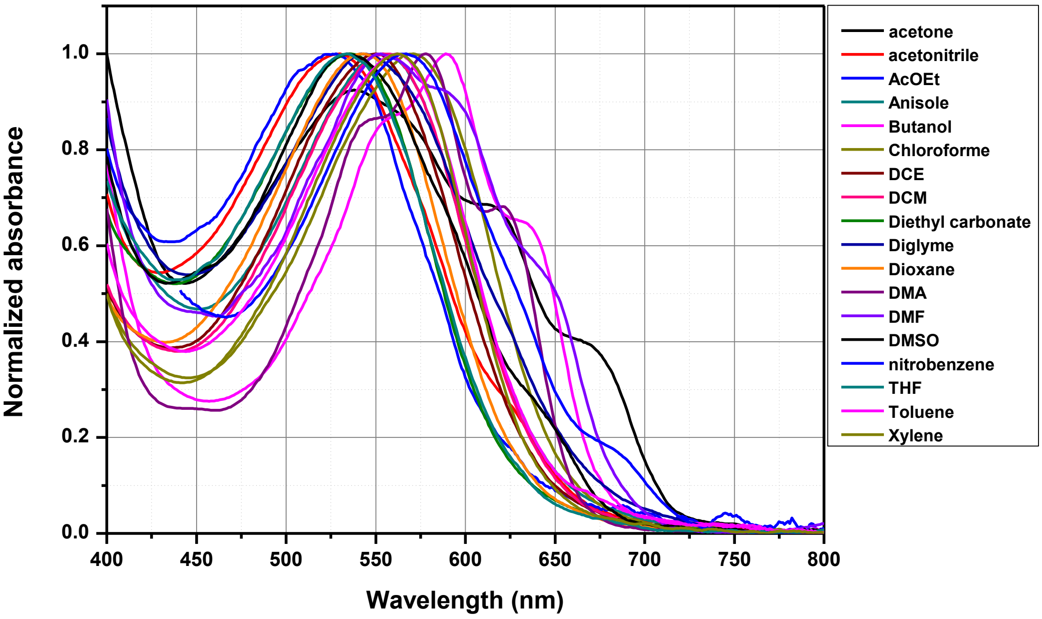

Figure S26. UV-visible absorption spectra of **Dye 6**.

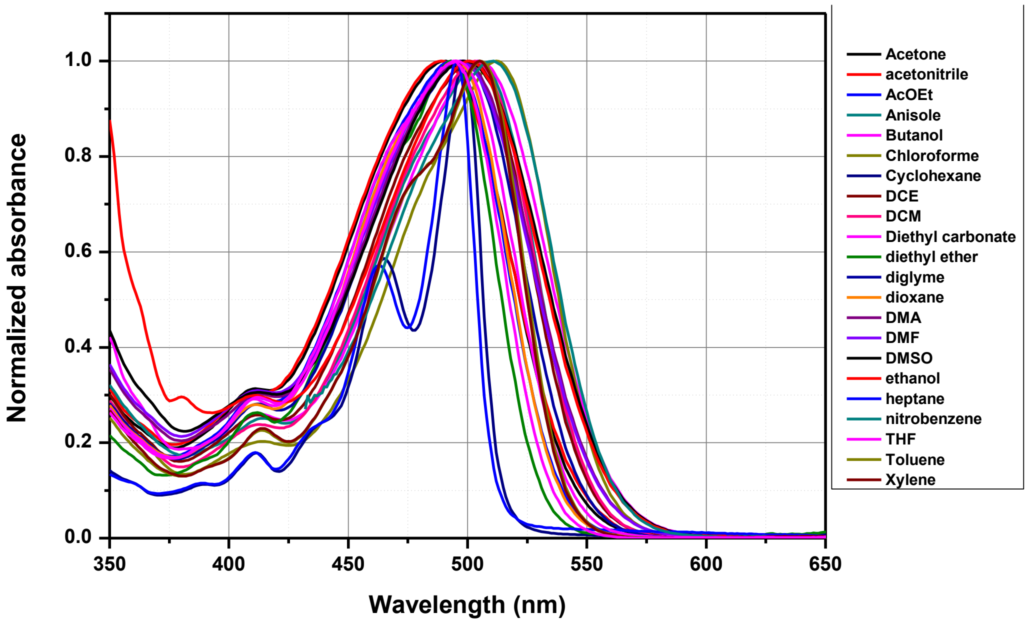

Figure S27. UV-visible absorption spectra of **Dye 7**.

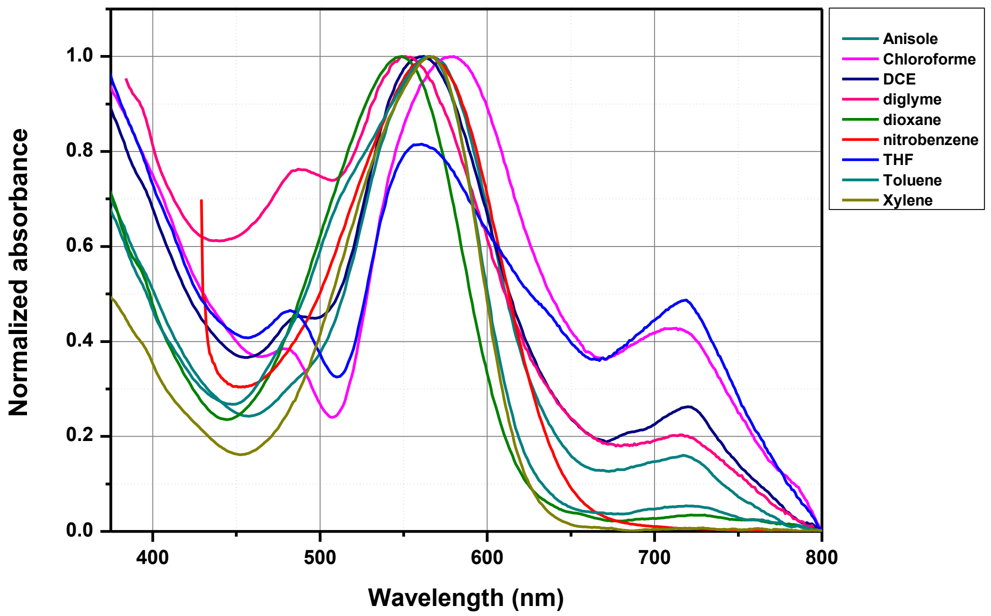

Figure S28. UV-visible absorption spectra of **Dye 8**.

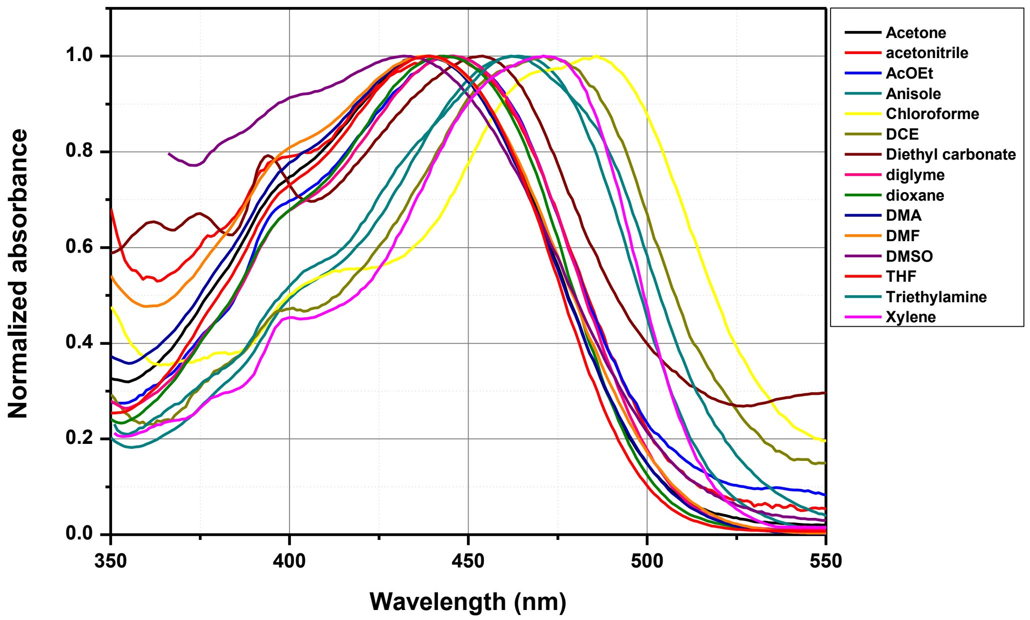

Figure S29. UV-visible absorption spectra of **Dye 9**.

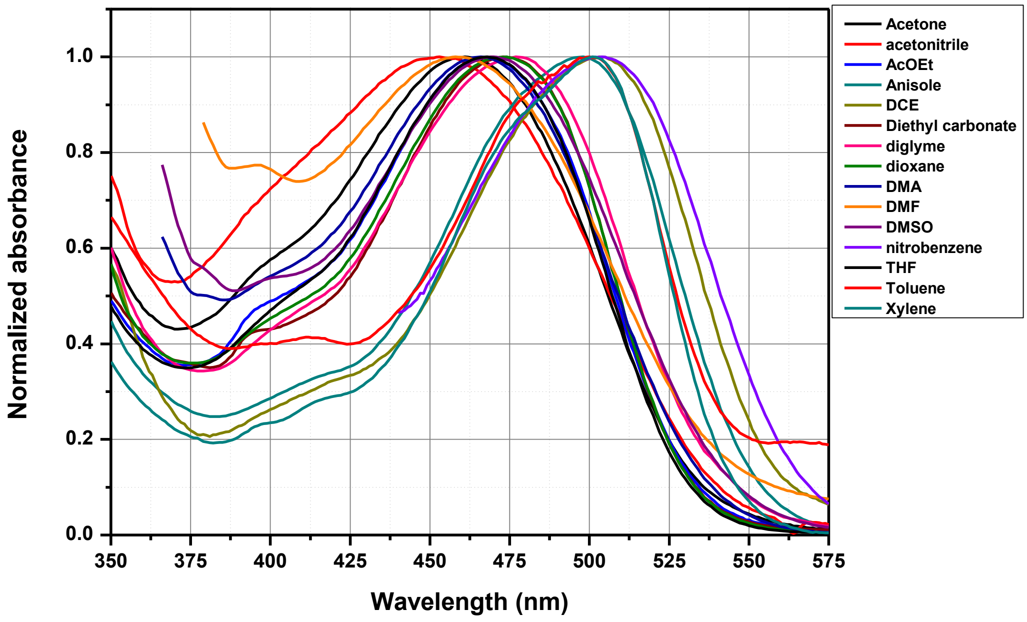

Figure S30. UV-visible absorption spectra of **Dye 10**.

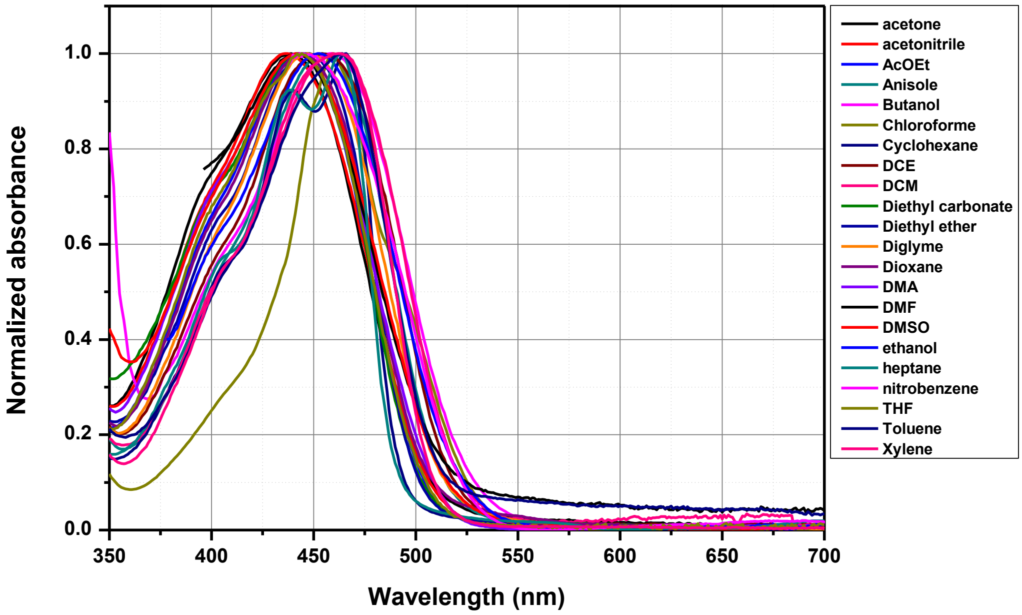

Figure S31. UV-visible absorption spectra of **Dye 11**.

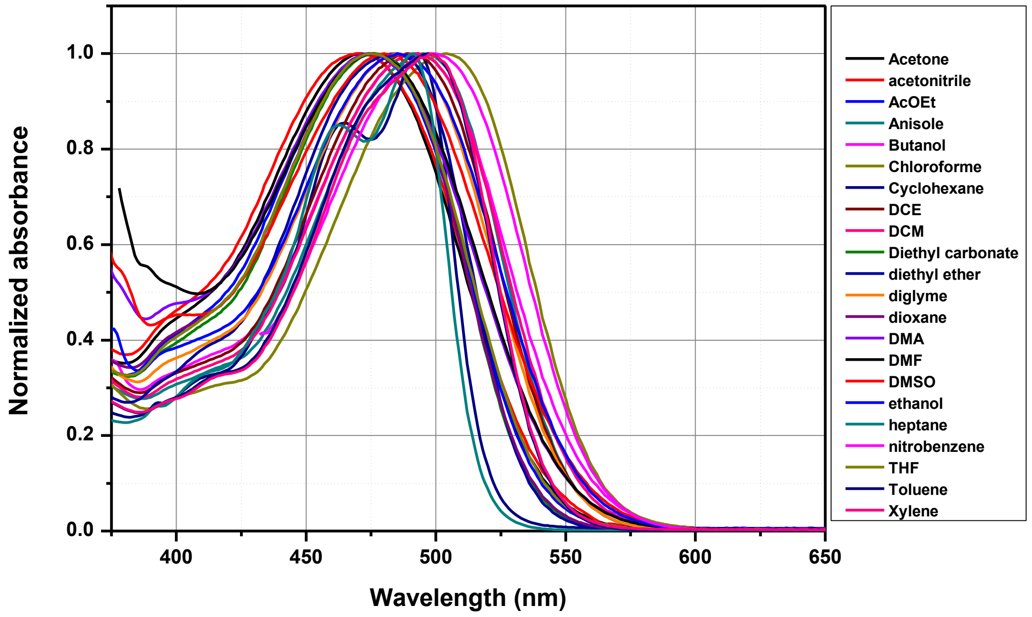

Figure S32. UV-visible absorption spectra of **Dye 12**.

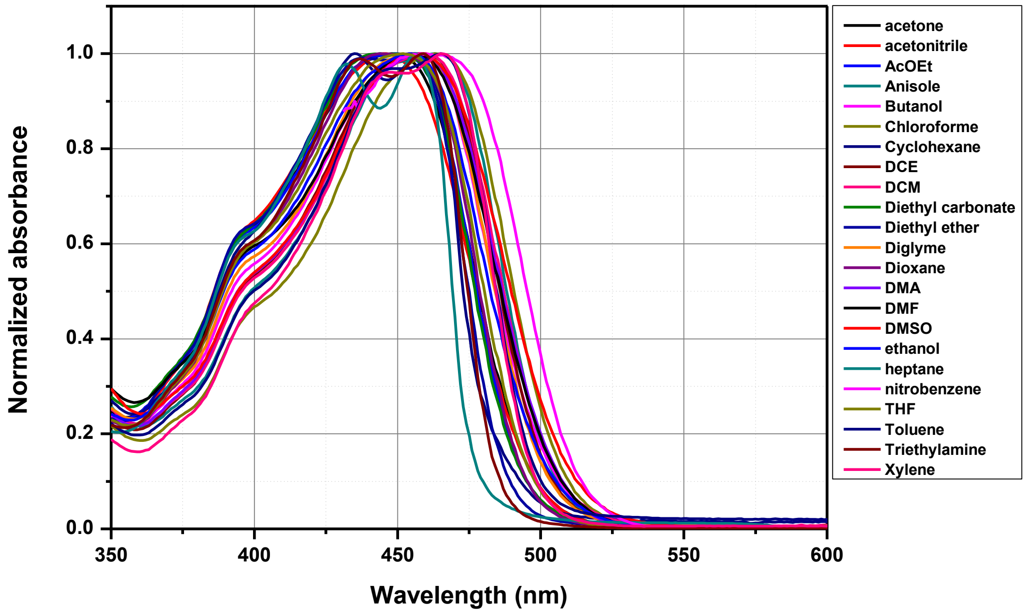

Figure S33. UV-visible absorption spectra of **Dye 13**.

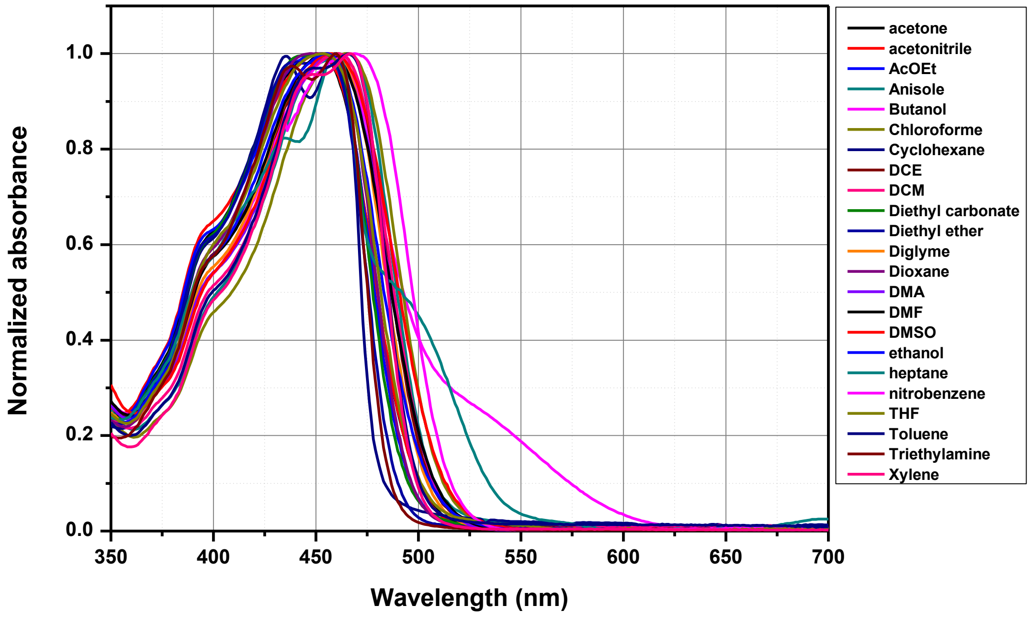

Figure S34. UV-visible absorption spectra of **Dye 14**.

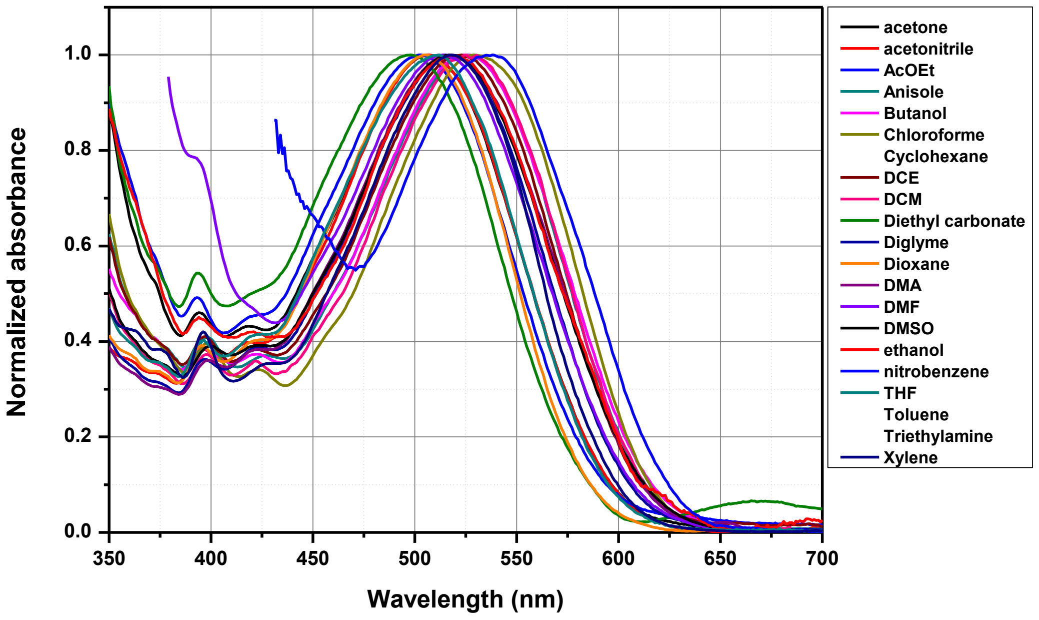

Figure S35. UV-visible absorption spectra of **Dye 15**.

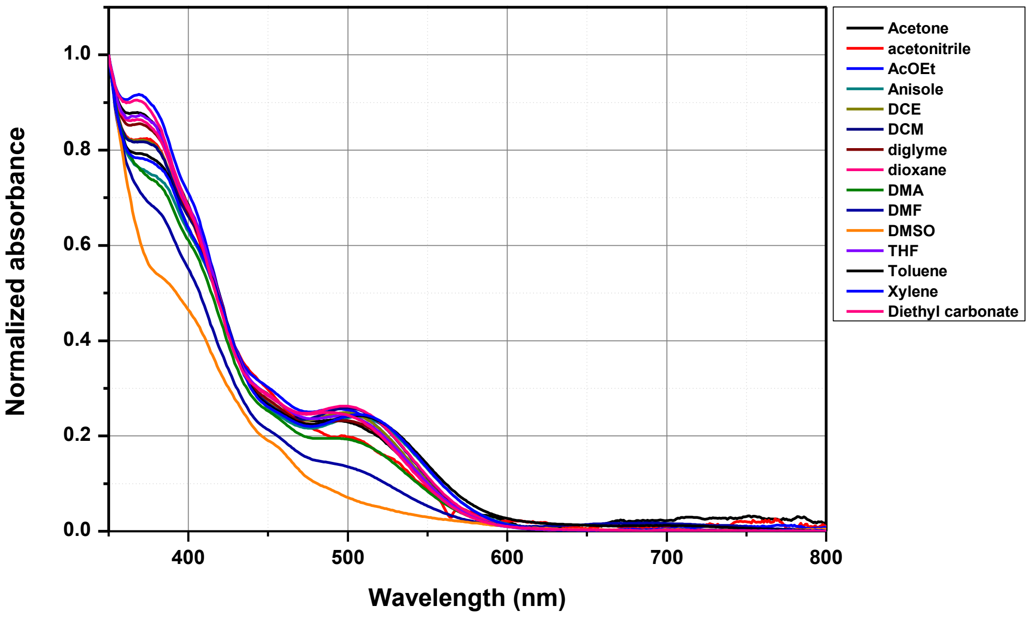

Position of the absorption maxima of PP1-PP15 in twenty-three solvents of different polarities vs. the Kamlet–Taft parameters π*

Figure S36. Dye 1

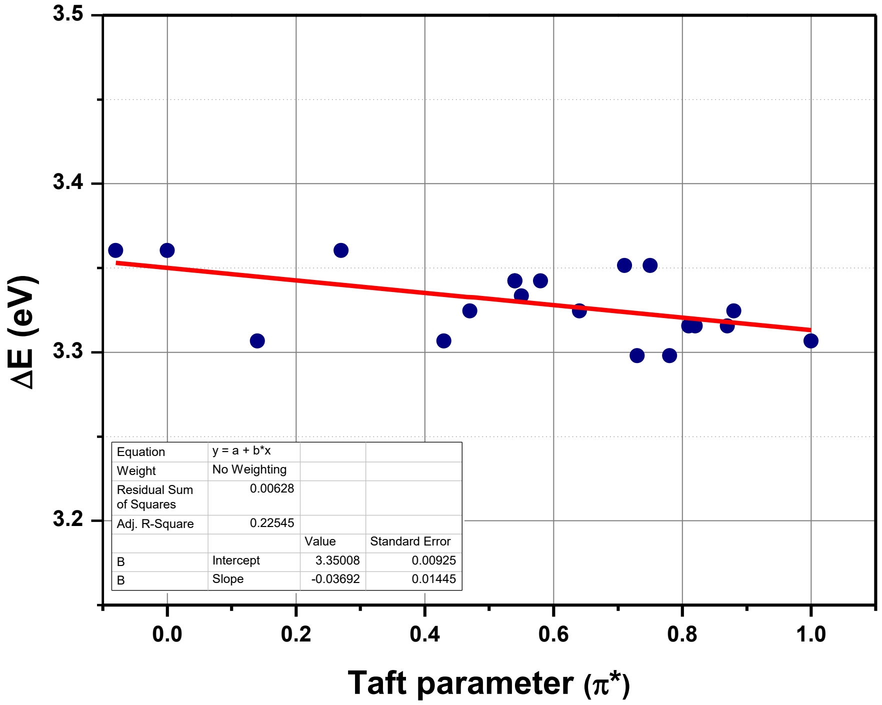

y = -0.03692 x + 3.35008 $R^2 = 0.22545$

Figure S37. Dye 2

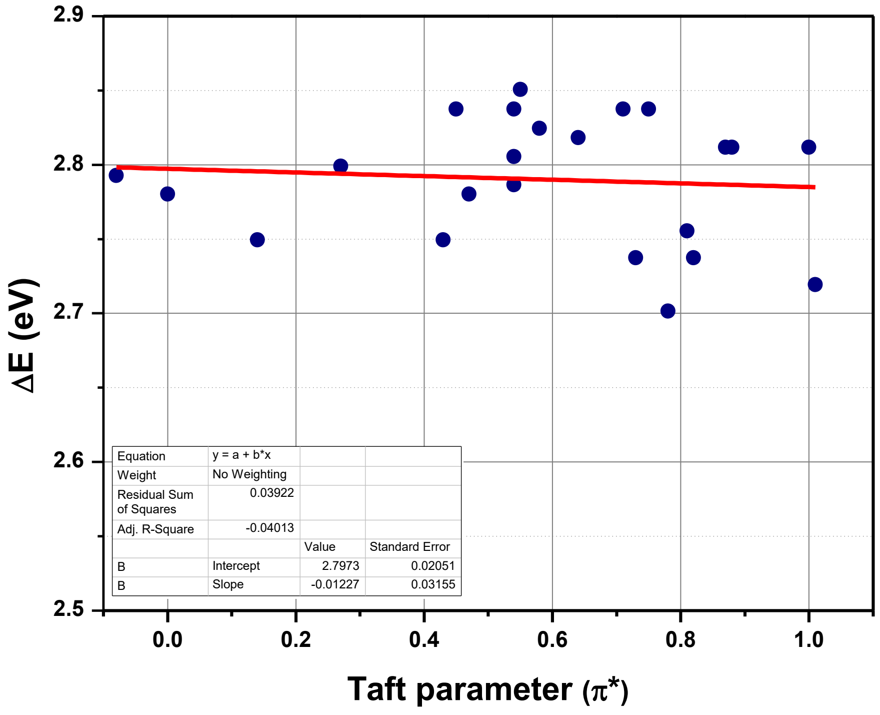

y = -0.01227 x + 2.7973 $R^2 = -0.040$

Figure S38. Dye 3

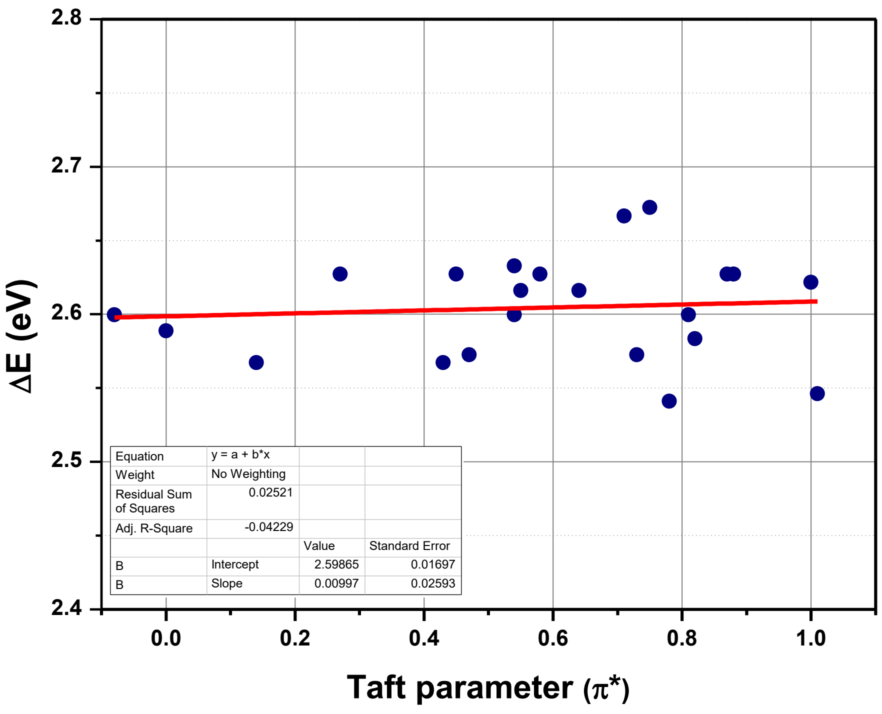

$y = 0.00997\ x + 2.59865$ $\quad\quad$ $R^2 = -0.042$

Figure S39. Dye 4

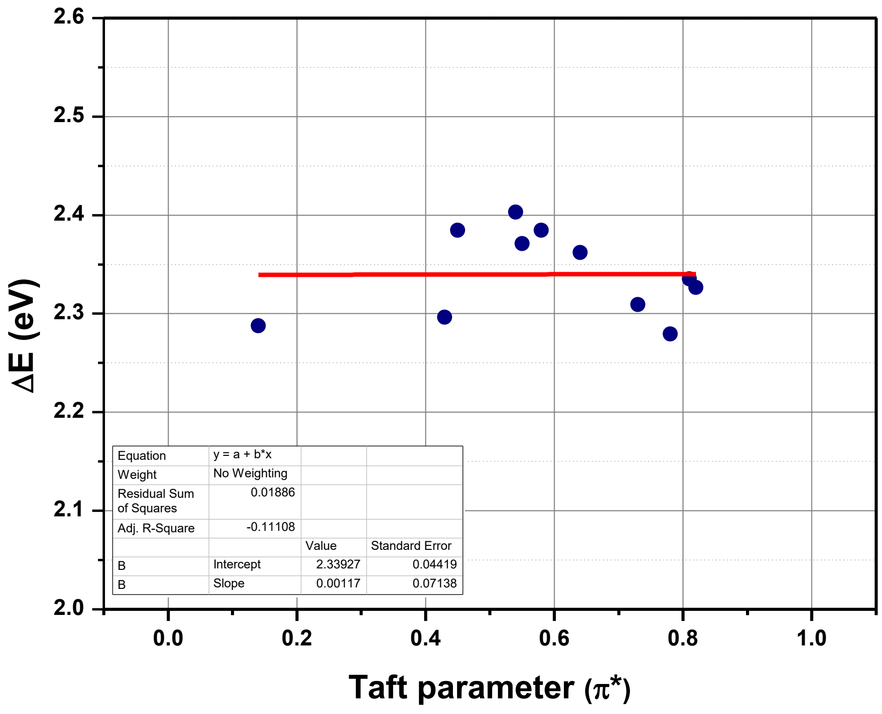

$y = 0.00117\ x + 2.33927$ $\quad\quad$ $R^2 = -0.111$

Figure S40. **Dye 5**

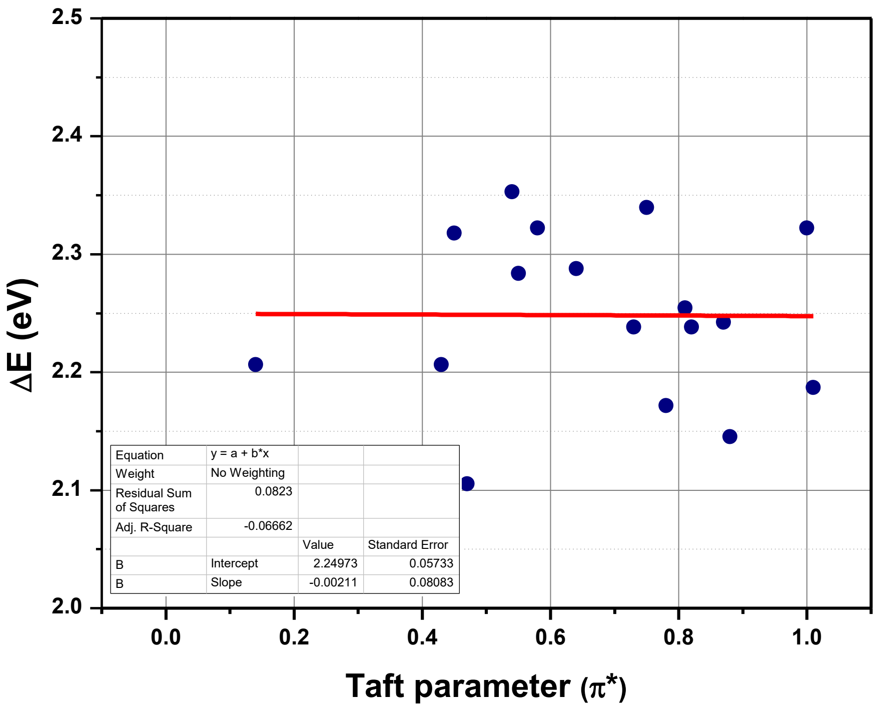

y = -0.00211 x + 2.24973 R^2 = -0.06662

Figure S41. **Dye 6**

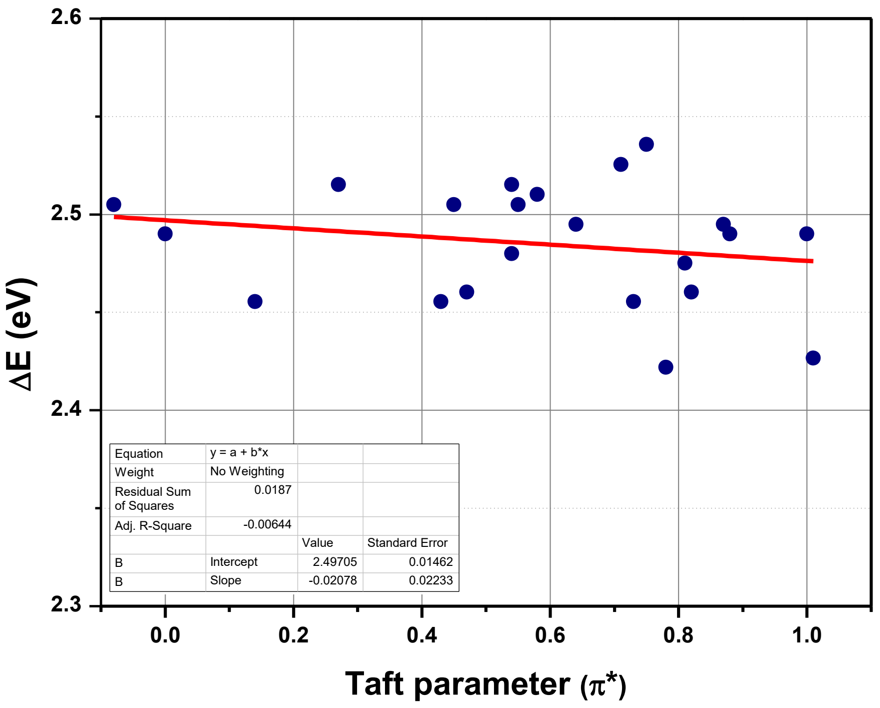

y = 0.02078 x + 2.49705 R^2 = -0.00644

Figure S42. **Dye 8**

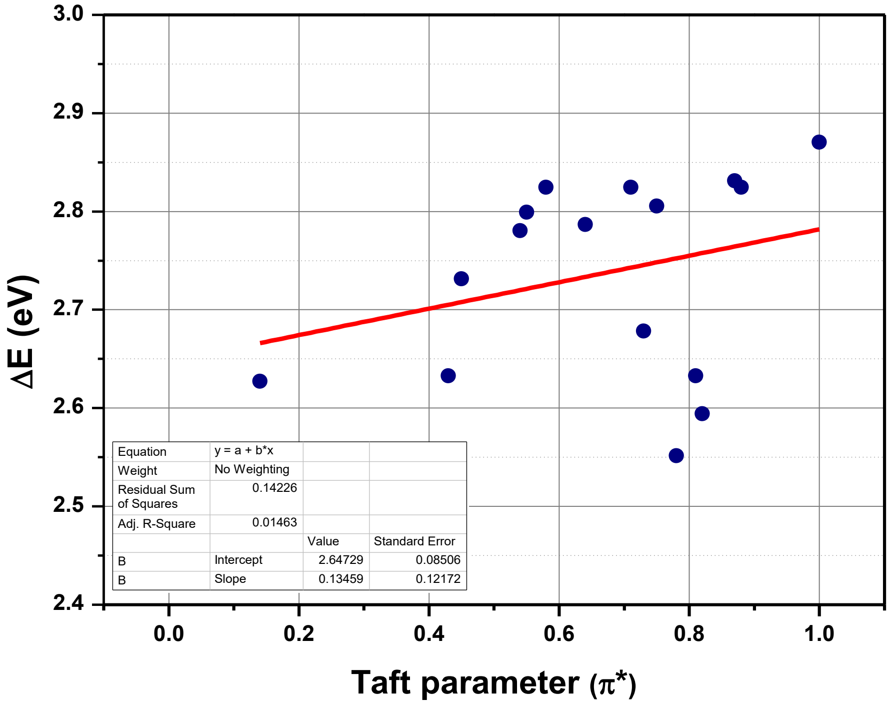

y = 0.13459 x + 2.64729 R^2 = 0.014

Figure S43. **Dye 9**

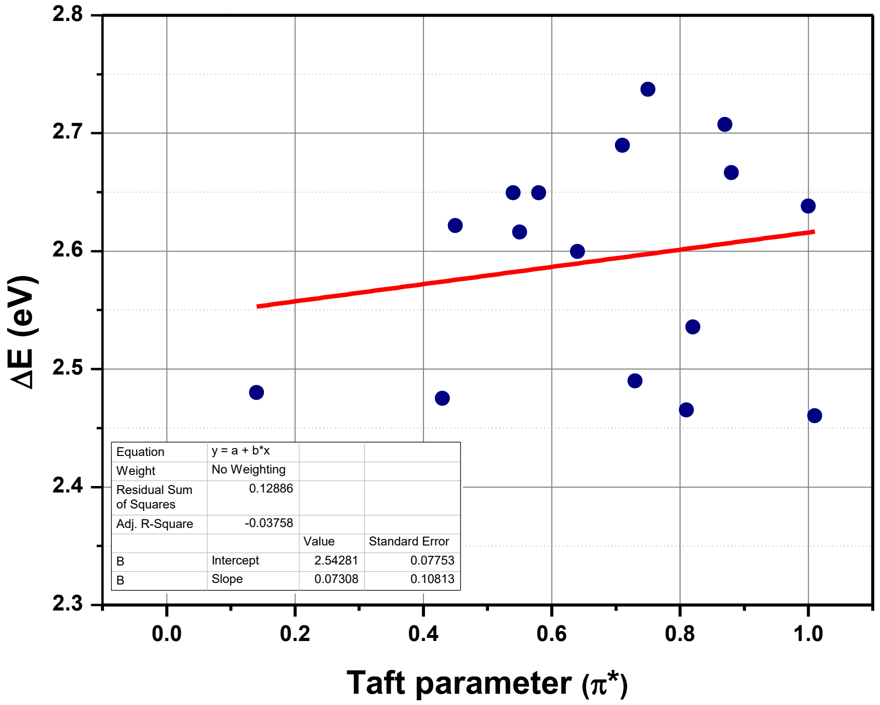

y = 0.0708 x + 2.54281 R^2 = -0.037

Figure S44. Dye 10

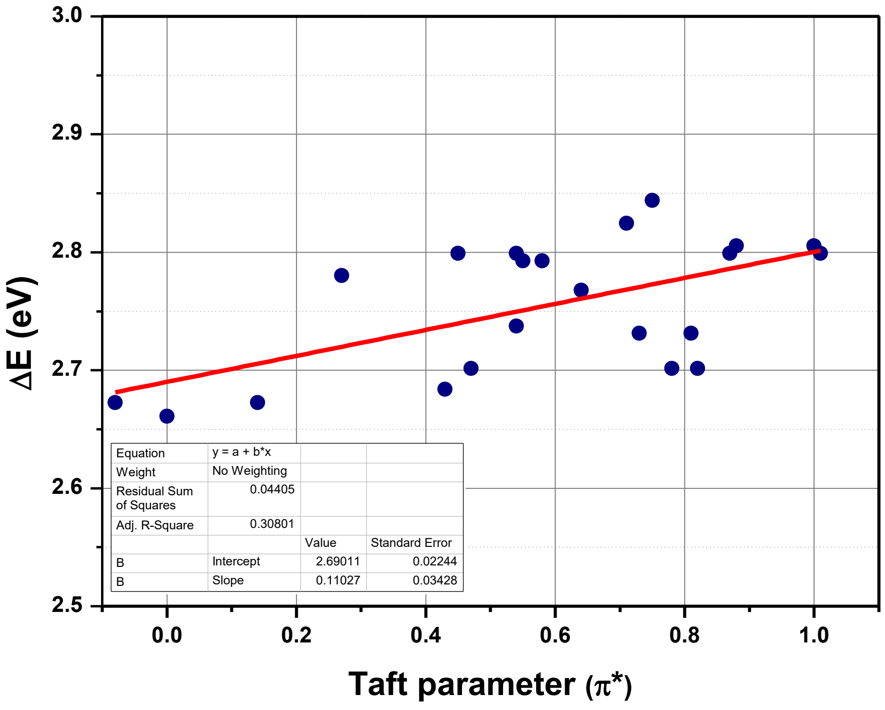

$y = 0.11027\ x + 2.69011$ $R^2 = 0.308$

Figure S45. Dye 11

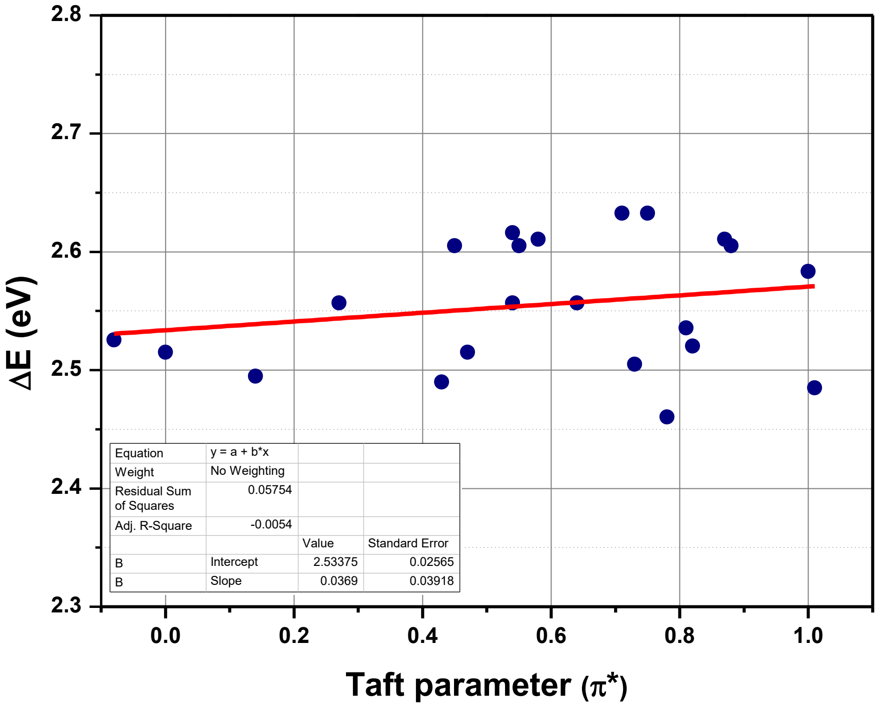

$y = 0.0369\ x + 2.53375$ $R^2 = -0.0054$

Figure S46. Dye 12

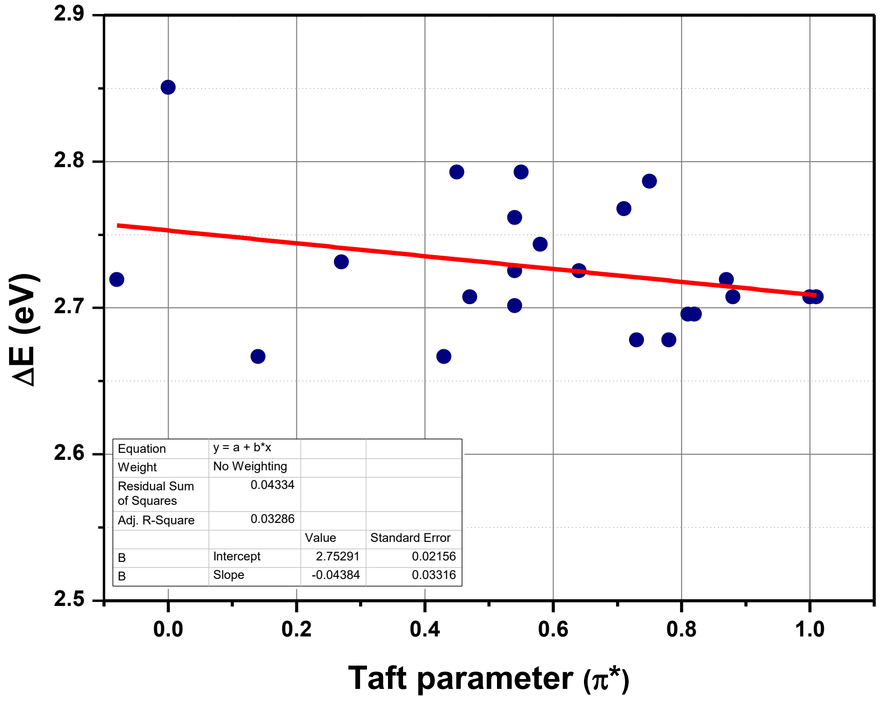

y = 0.04384 x + 2.75291 R^2 = 0.03286

Figure S47. Dye 13

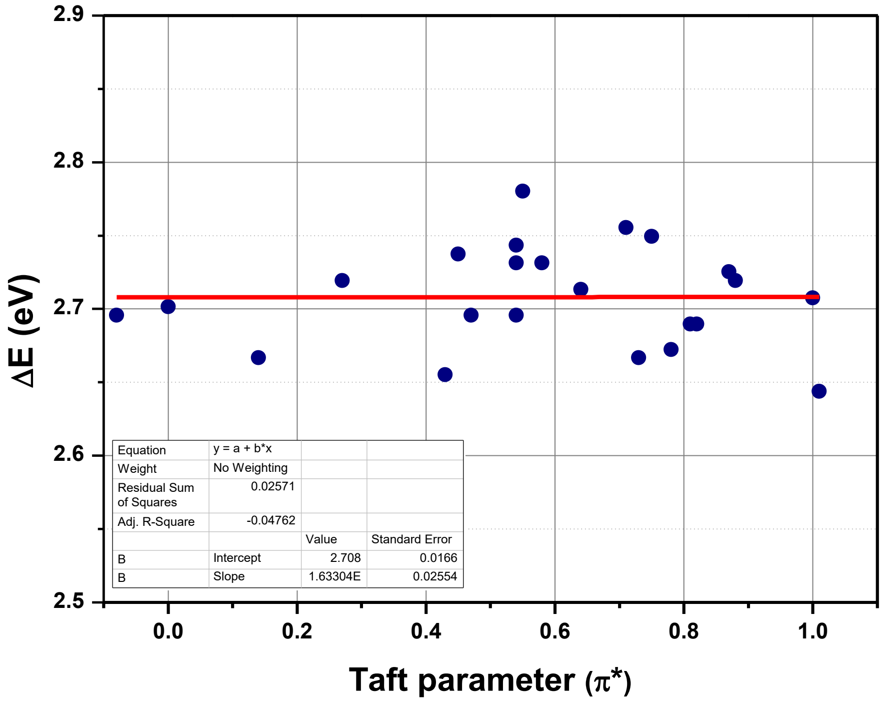

y = 0.1633 x + 2.708 R^2 = -0.0476

Figure S48. Dye 14

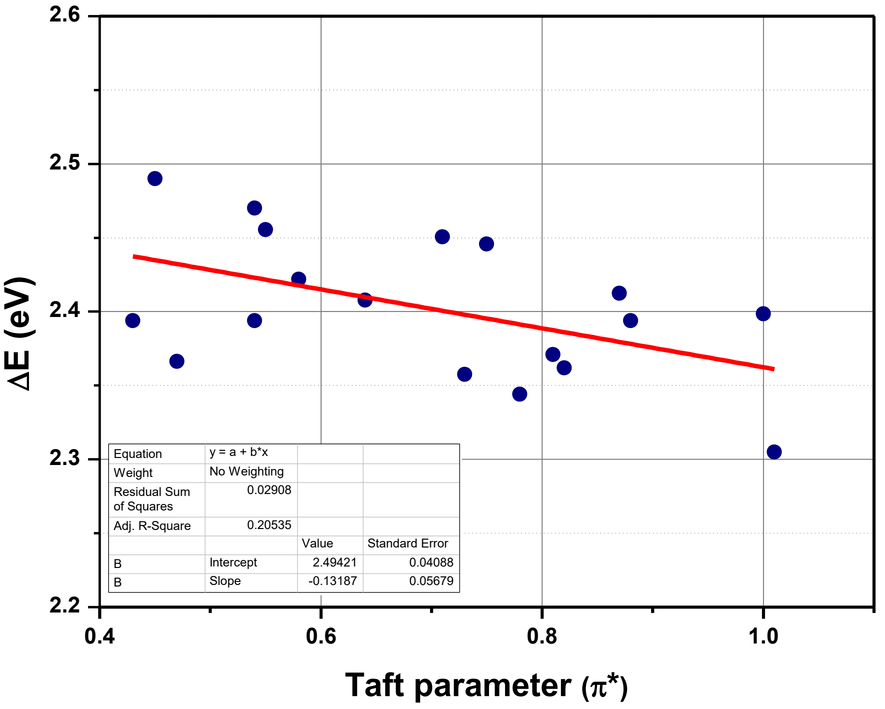

y = -0.13187 x + 2.49421 R^2 = 0.205

Figure S49. Dye 15

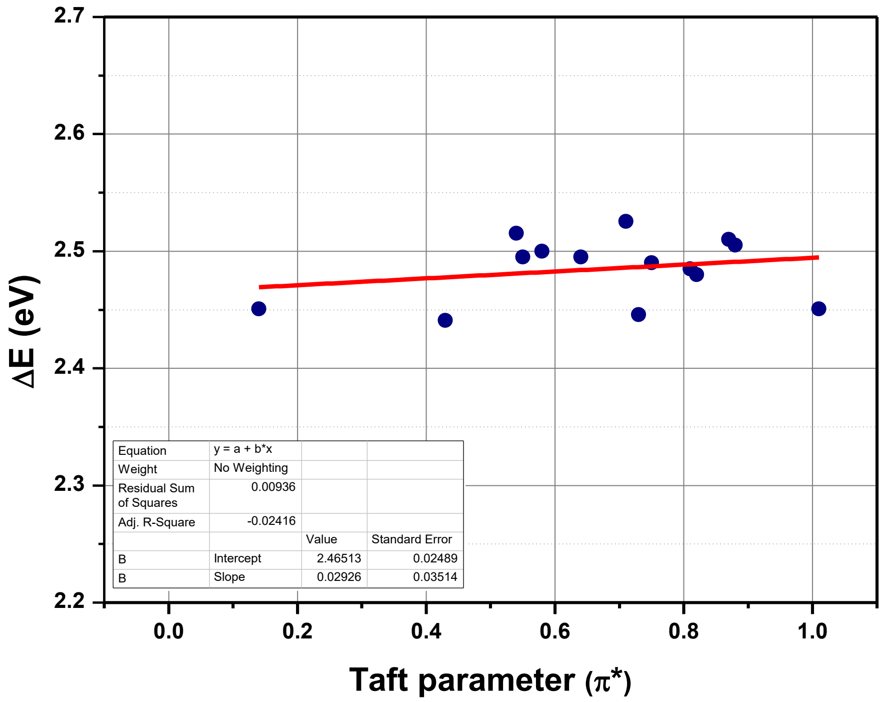

y = -0.02926 x + 2.46513 R^2 = -0.0242

Position of the absorption maxima of PP1-PP15 in twenty-three solvents of different polarities vs. the Catalan solvent polarity/polarizability (SPP) scale and the Catalan solvent dipolarity (SdP) scale

Figure S50. Dye 1

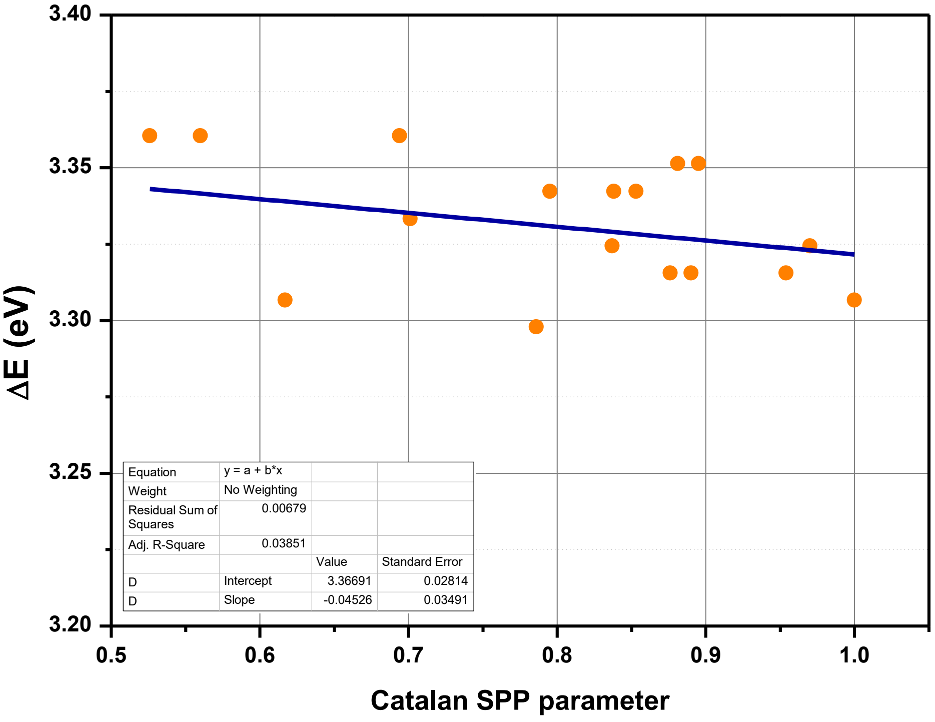

y = -0.04526 x + 2.49421 R^2 = 0.205

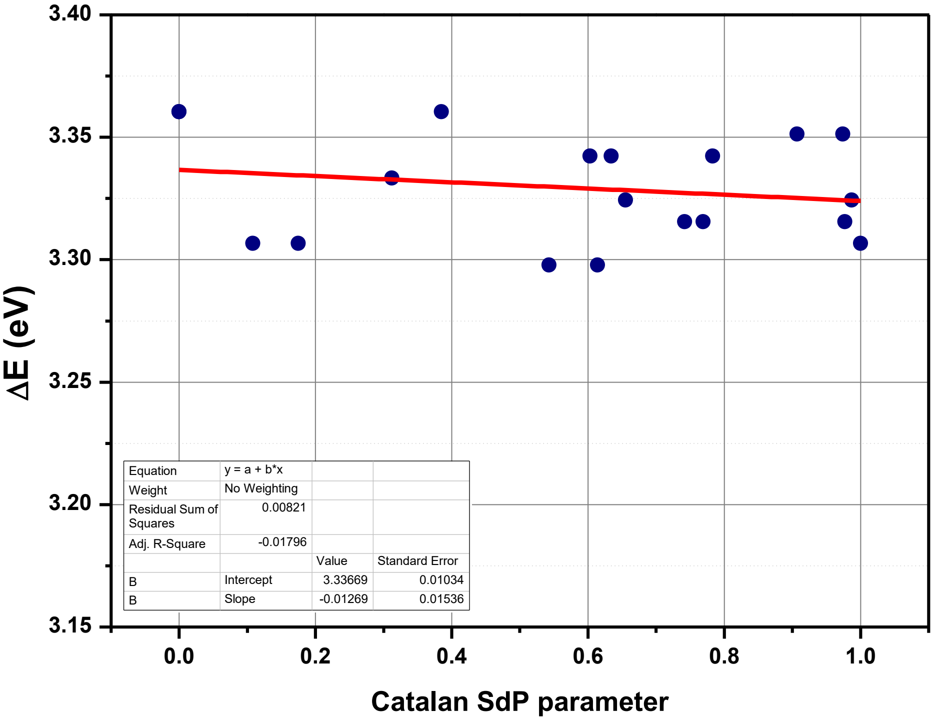

y = -0.01269 x + 3.33669 R^2 = -0.018

Figure S51. Dye2

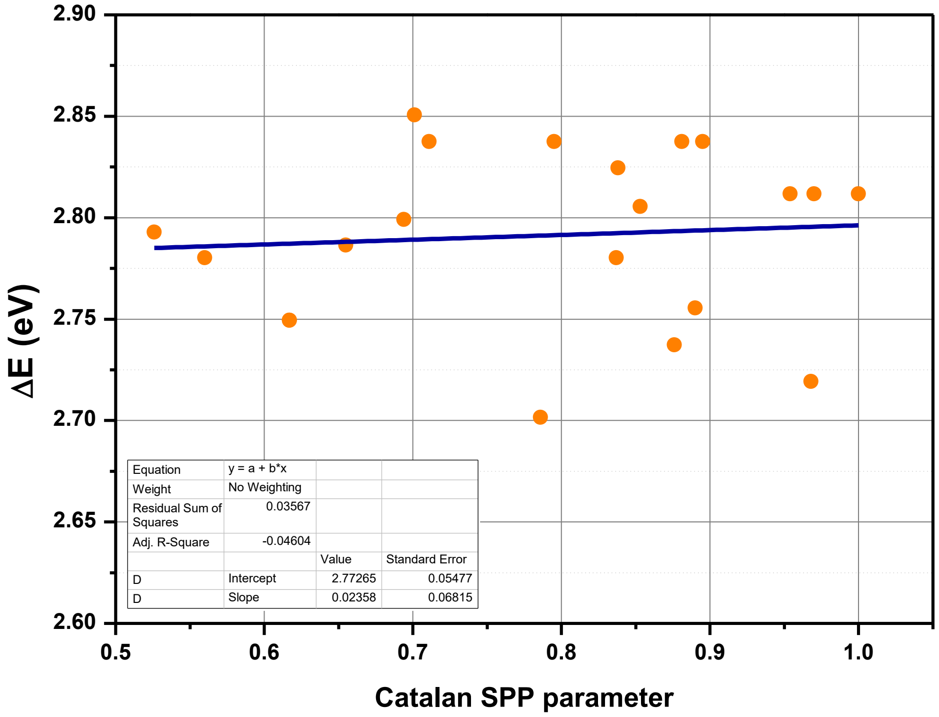

y = 0.02358 x + 2.77265 R^2 = -0.046

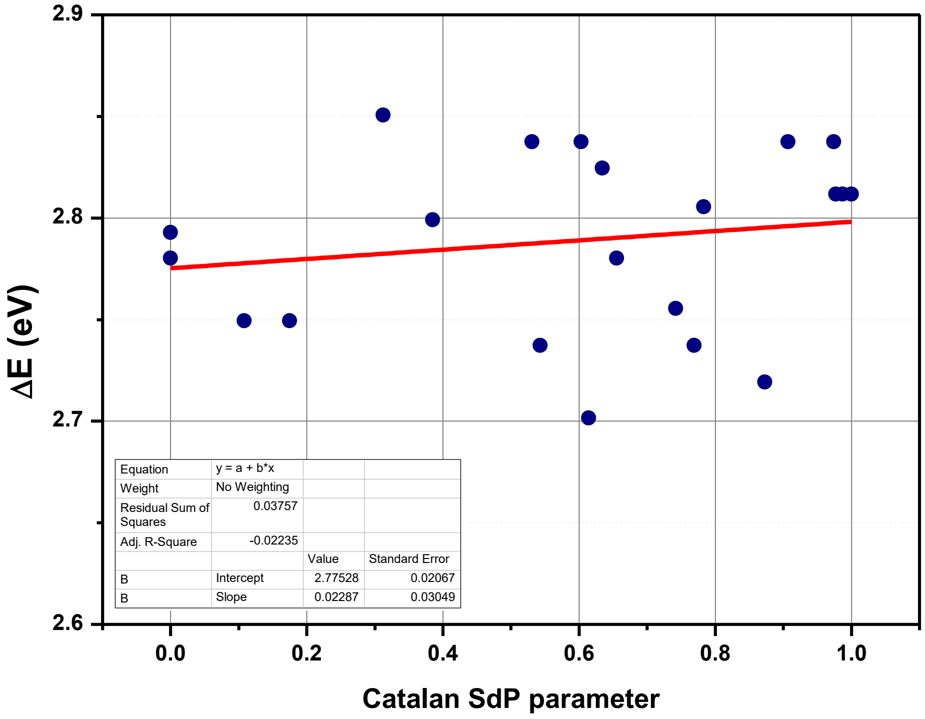

y = 0.02287 x + 2.77528 R^2 = -0.022

Figure S52. Dye 3

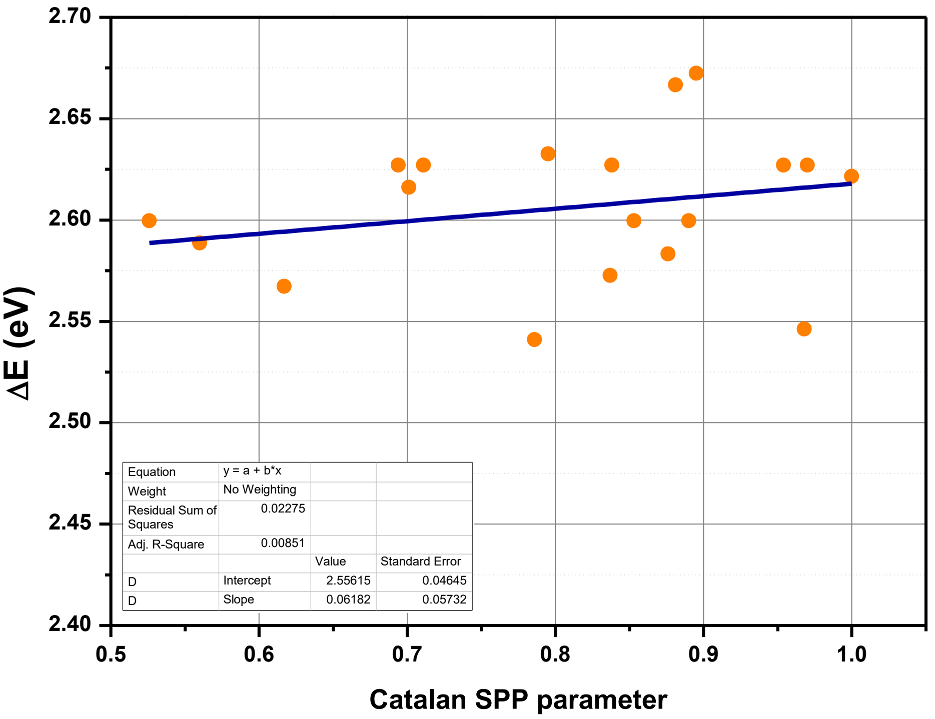

y = 0.06182 x + 2.55615 R^2 = -0.00851

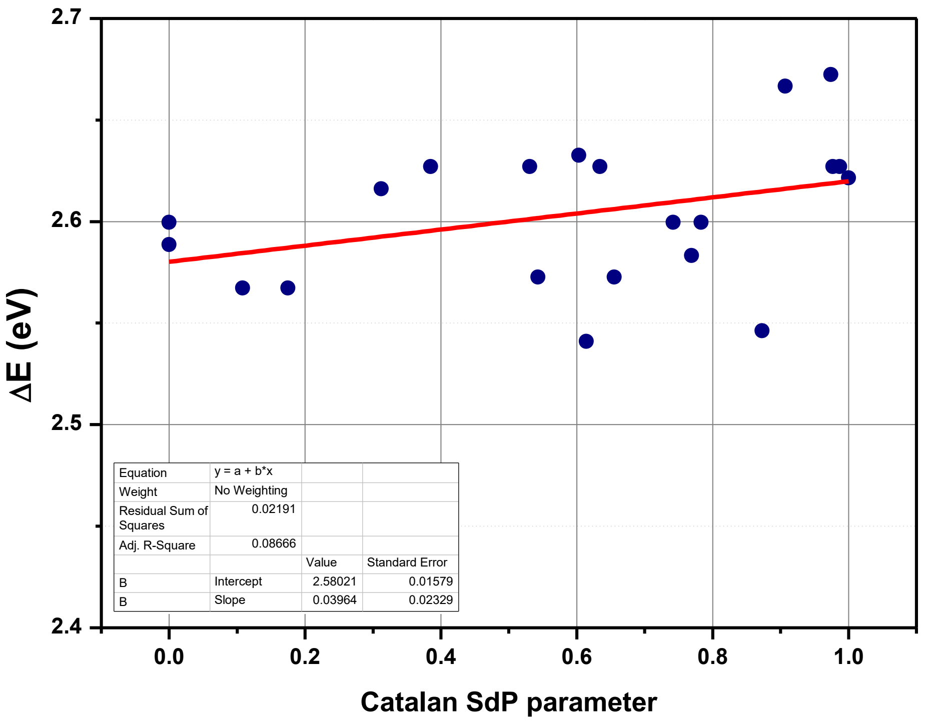

y = 0.03964 x + 2.58021 R^2 = 0.087

Figure S53. Dye 5

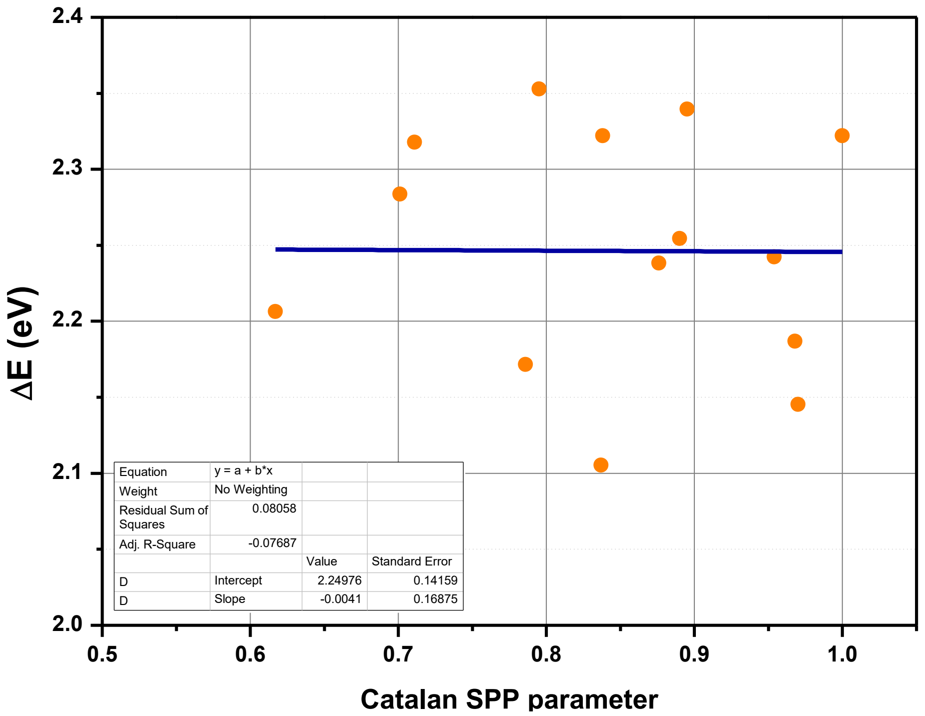

$y = -0.0041\ x + 2.24976$ $R^2 = -0.077$

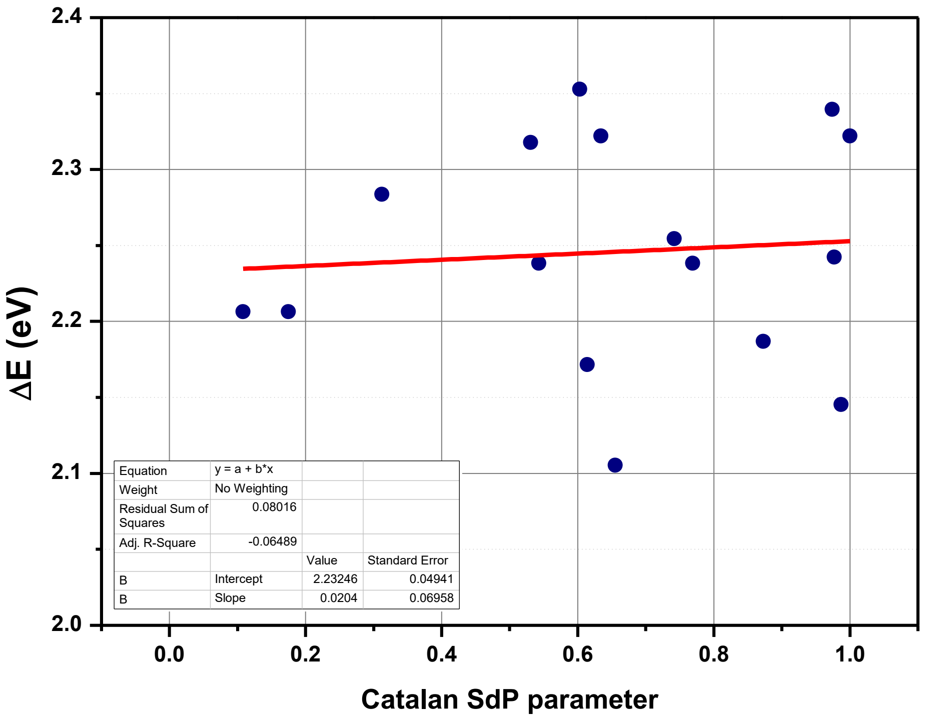

$y = 0.0204\ x + 2.23246$ $R^2 = -0.065$

Figure S54. **Dye 6**

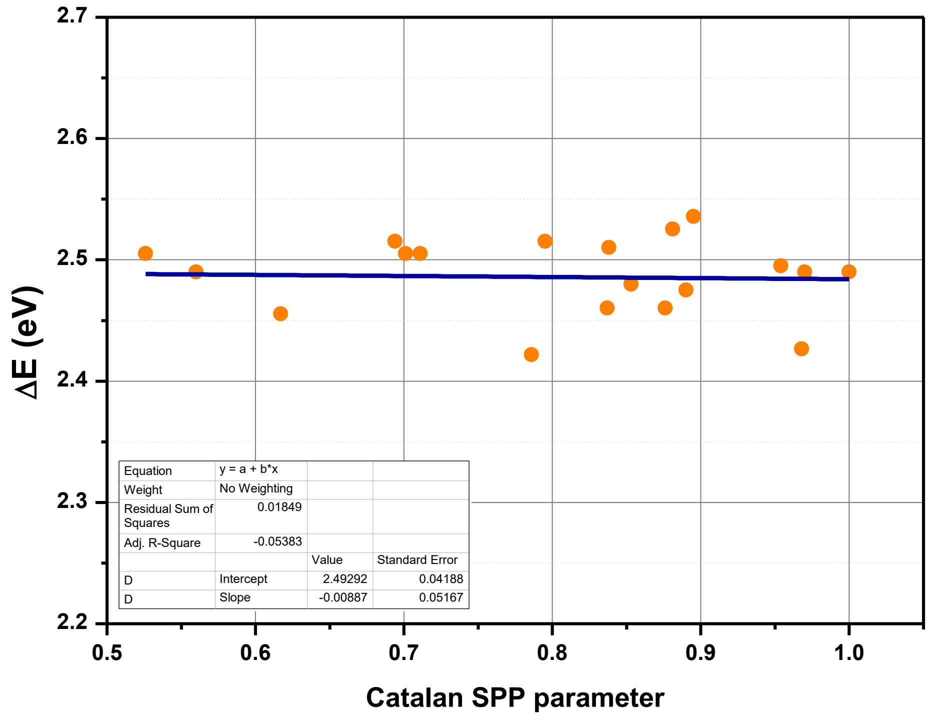

y = -0.0887 x + 2.49292 R^2 = -0.054

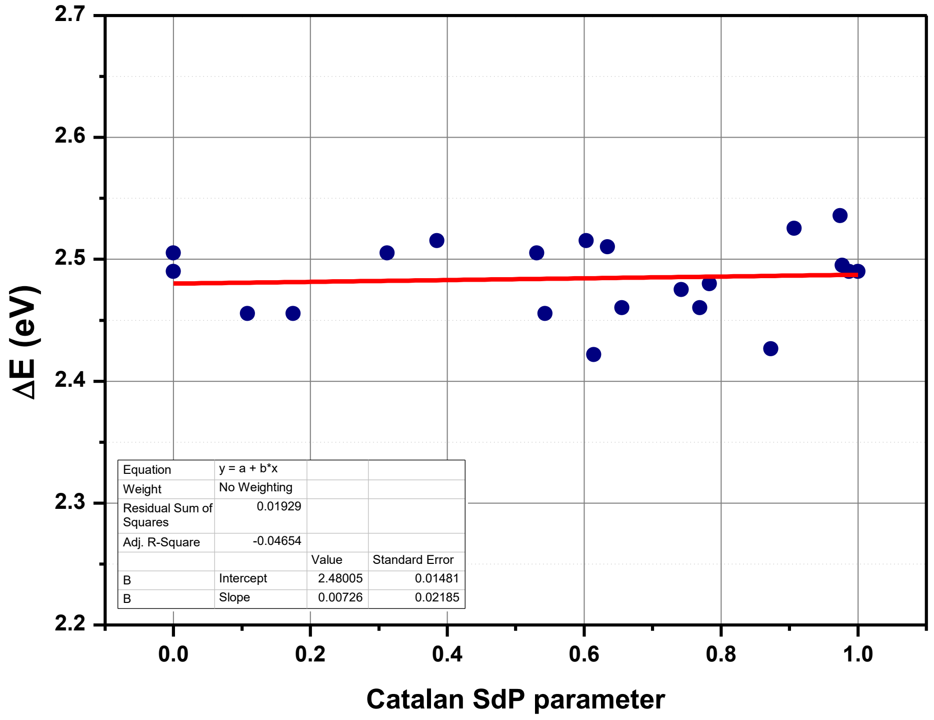

y = 0.00726 x + 2.48005 R^2 = -0.046

Figure S55. Dye 8

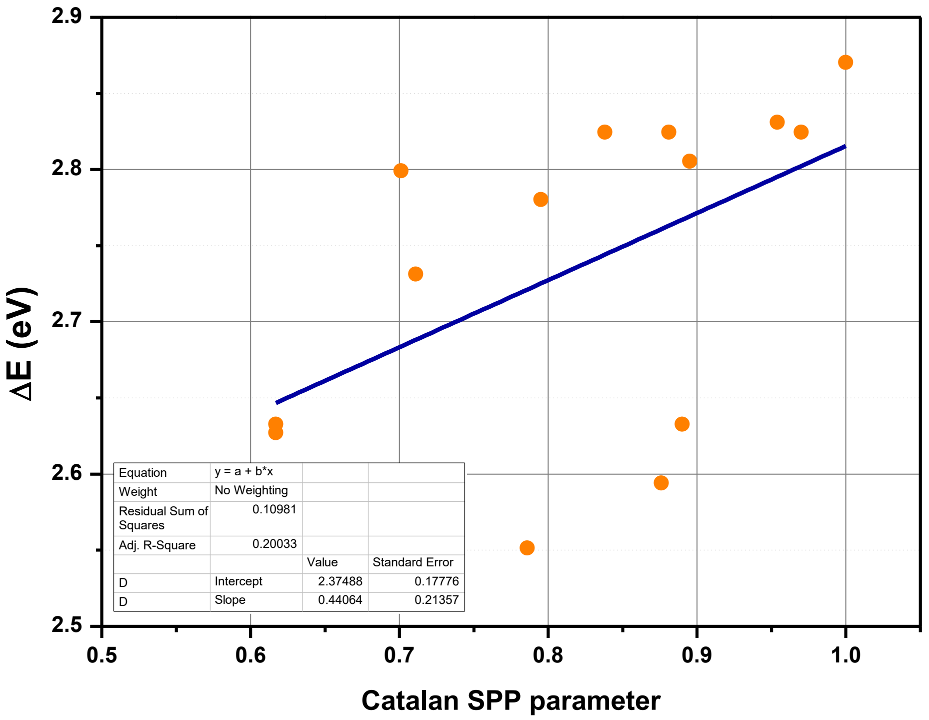

y = 0.44064 x + 2.37488 R^2 = 0.2003

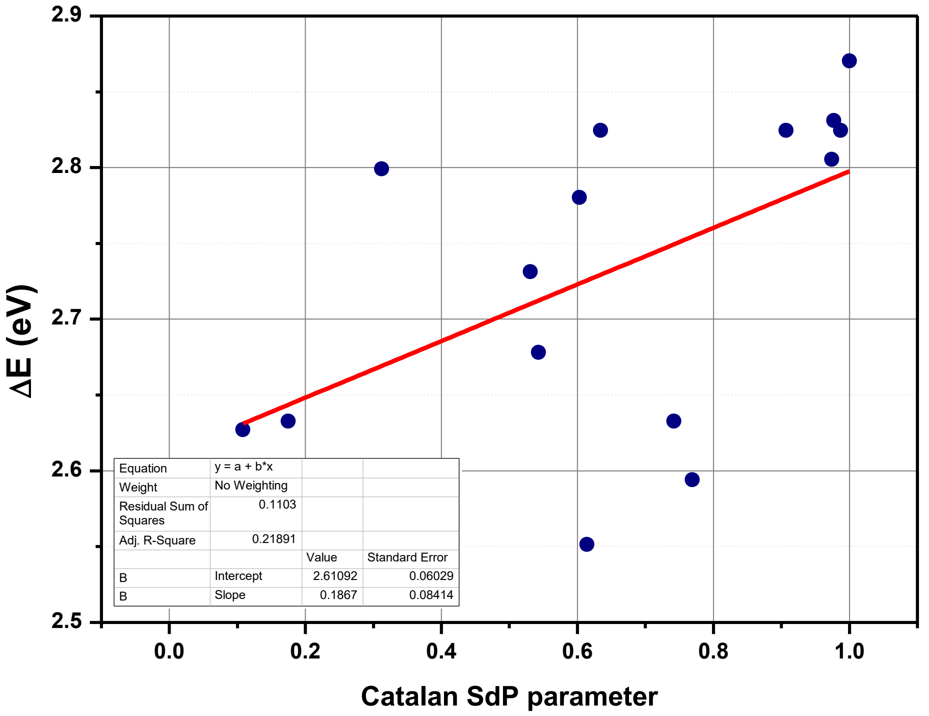

y = 0.1867 x + 2.61092 R^2 = 0.2189

Figure S56. Dye 9

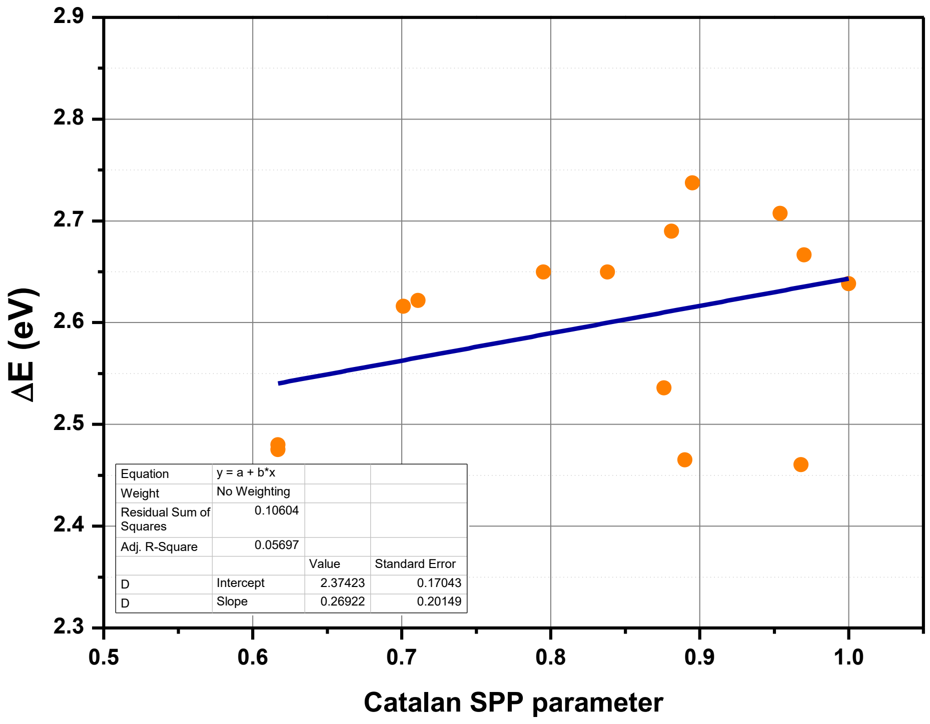

y = 0.26922 x + 2.37423 R^2 = 0.0570

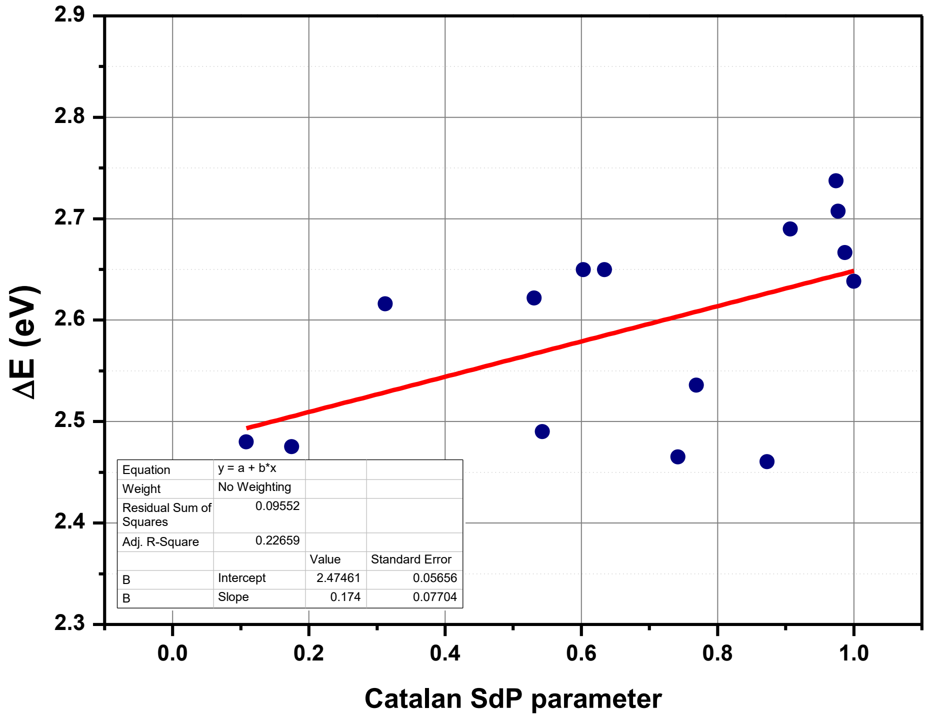

y = 0.174 x + 2.4746 R^2 = 0.2266

Figure S57. Dye 10

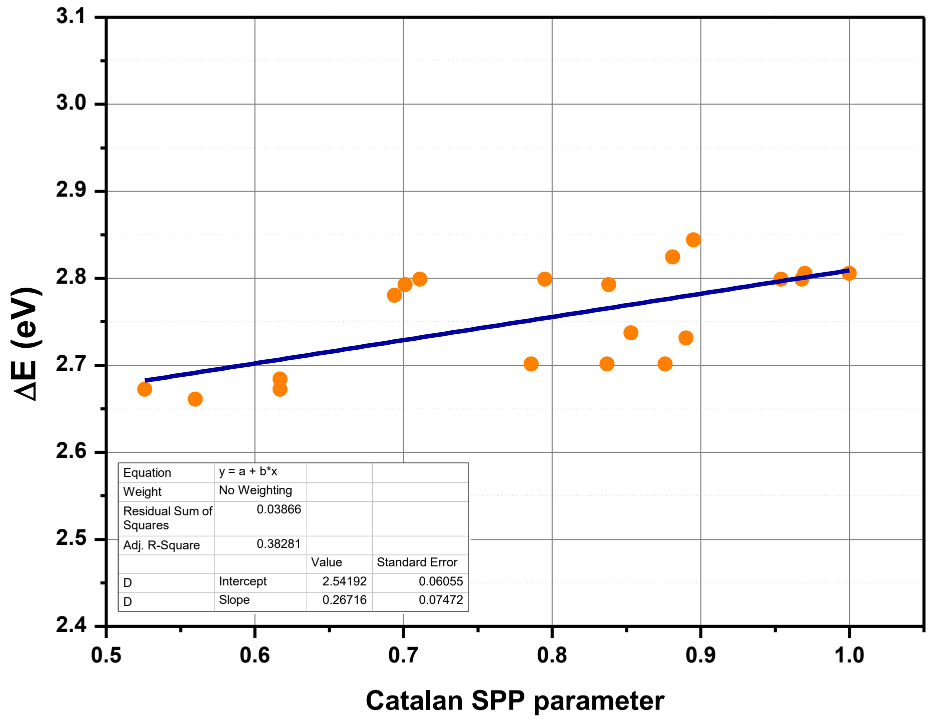

y = 0.26716 x + 2.5419 R^2 = 0.3828

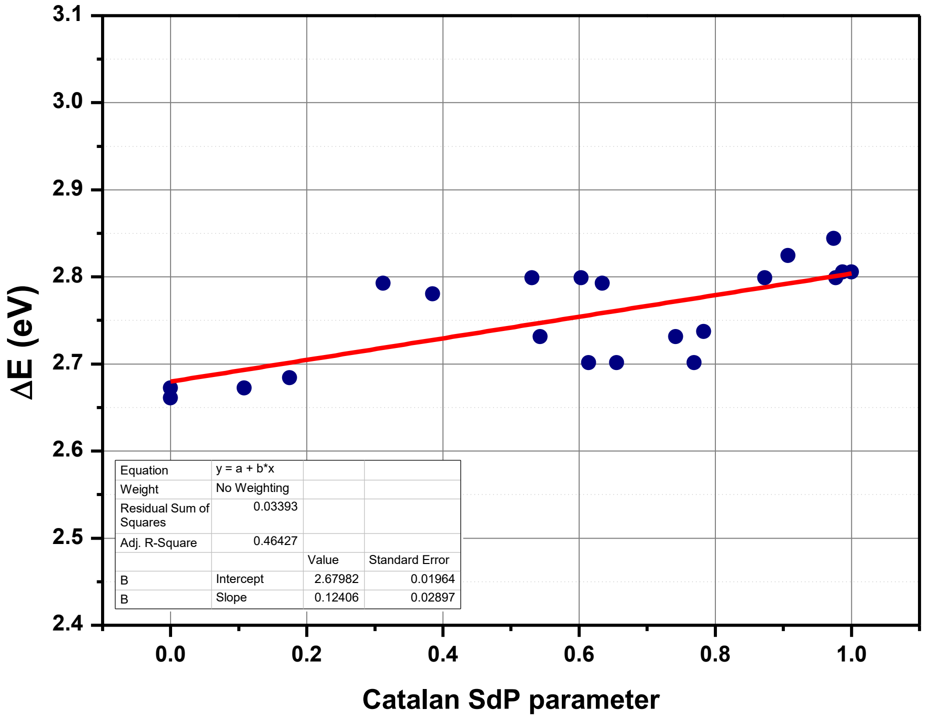

y = 0.1241 x + 2.6798 R^2 = 0.4643

Figure S58. Dye 11

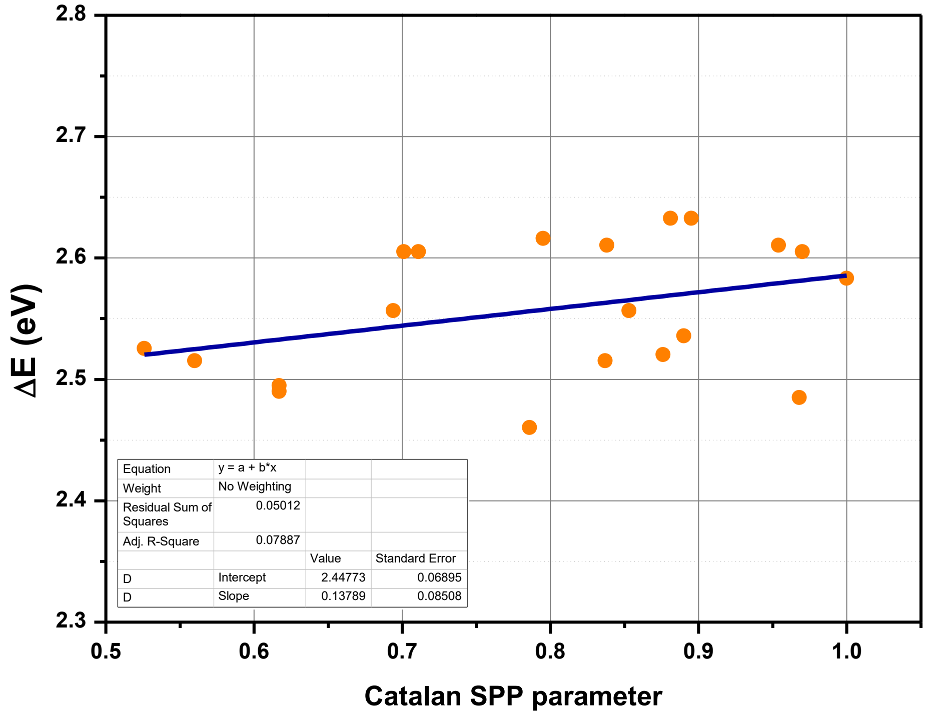

$y = 0.1379\ x + 2.4477$ $R^2 = 0.0789$

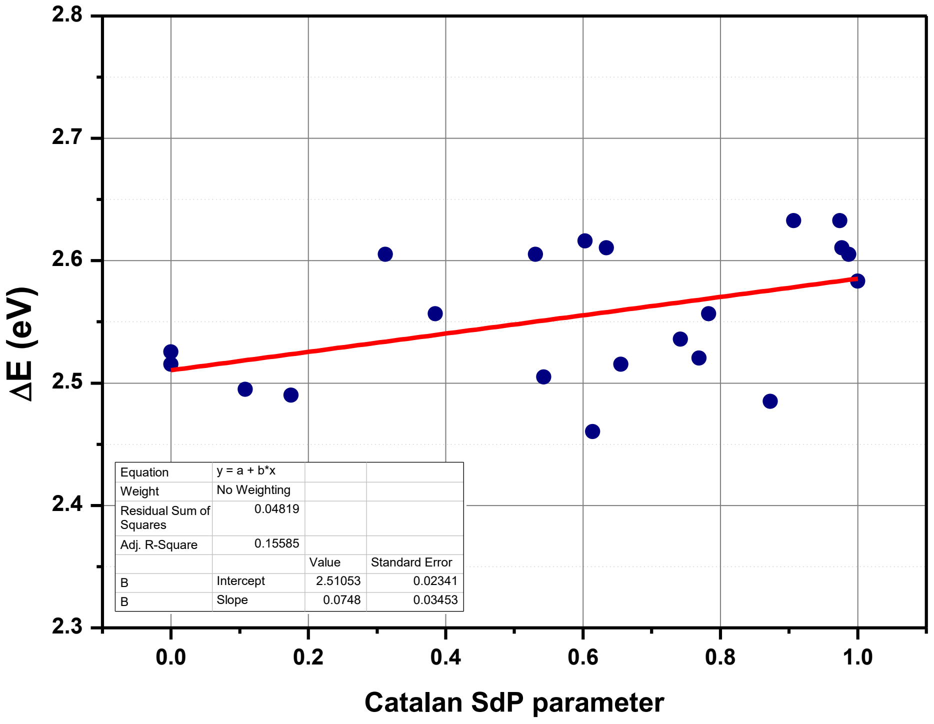

$y = 0.0748\ x + 2.5105$ $R^2 = 0.1558$

Figure S59. Dye 12

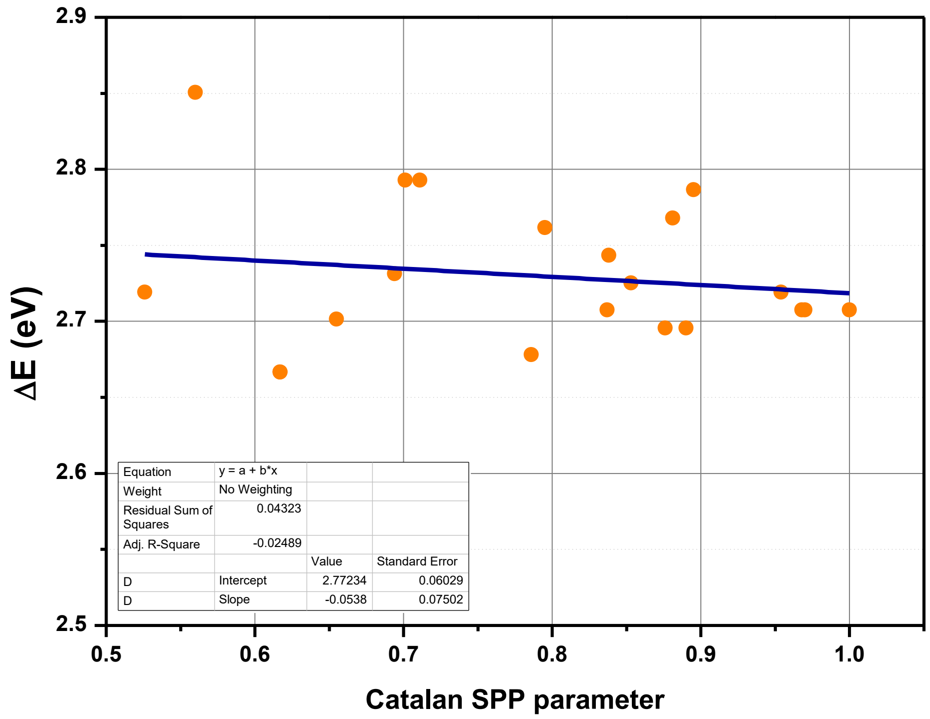

y = -0.0538 x + 2.7723 R^2 = -0.0249

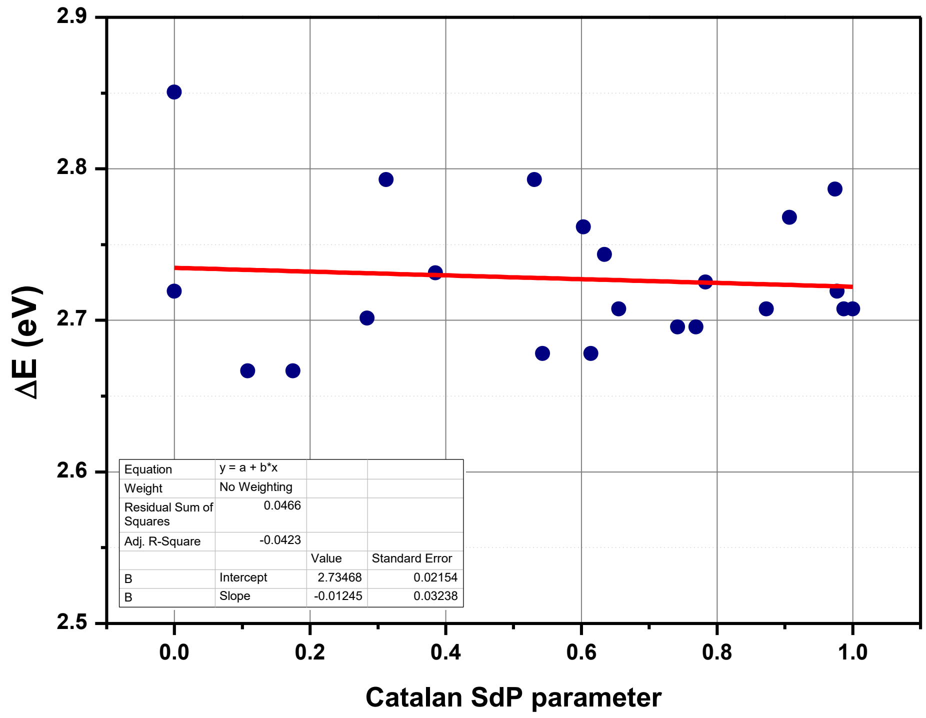

y = -0.01245 x + 2.7347 R^2 = -0.0423

Figure S60. Dye 13

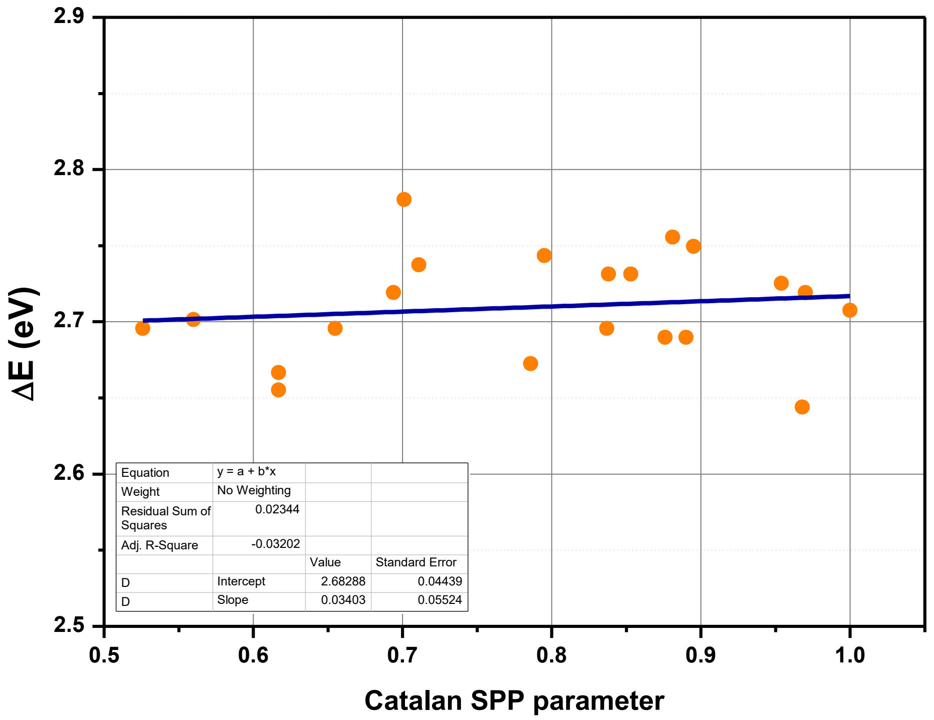

$y = 0.03403\ x + 2.6829$ $R^2 = -0.032$

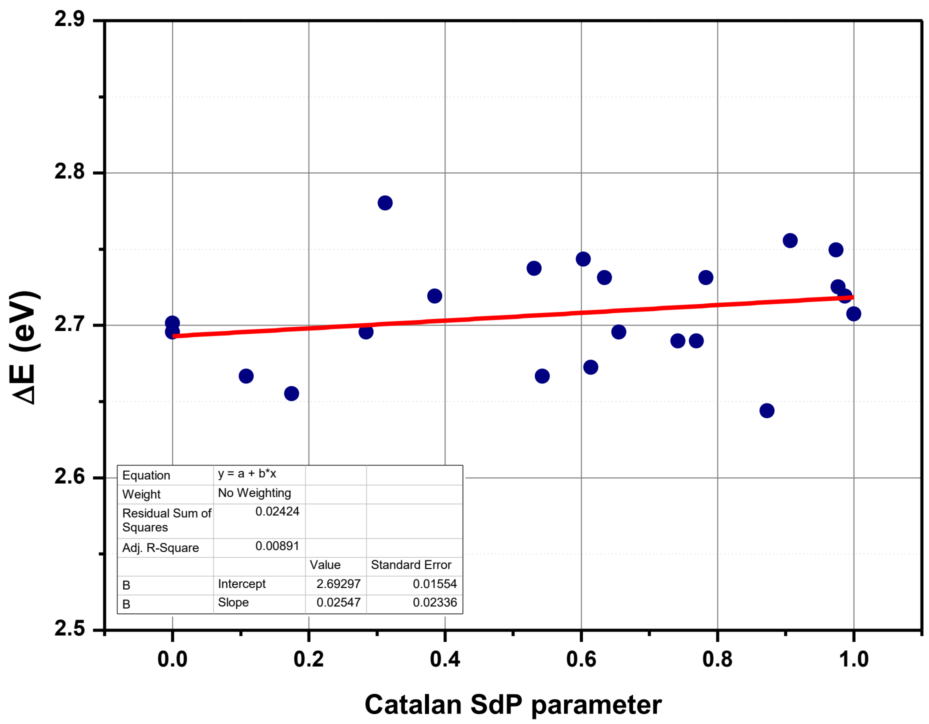

$y = 0.02547\ x + 2.69297$ $R^2 = 0.0089$

Figure S61. Dye 14

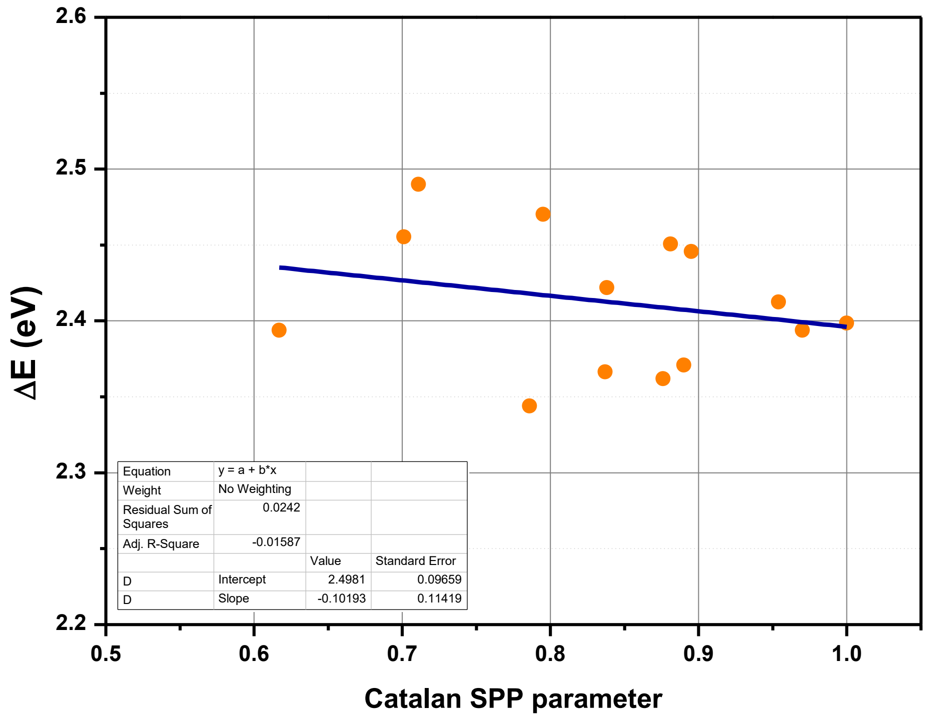

y = -0.10193 x + 2.4981 R^2 = -0.016

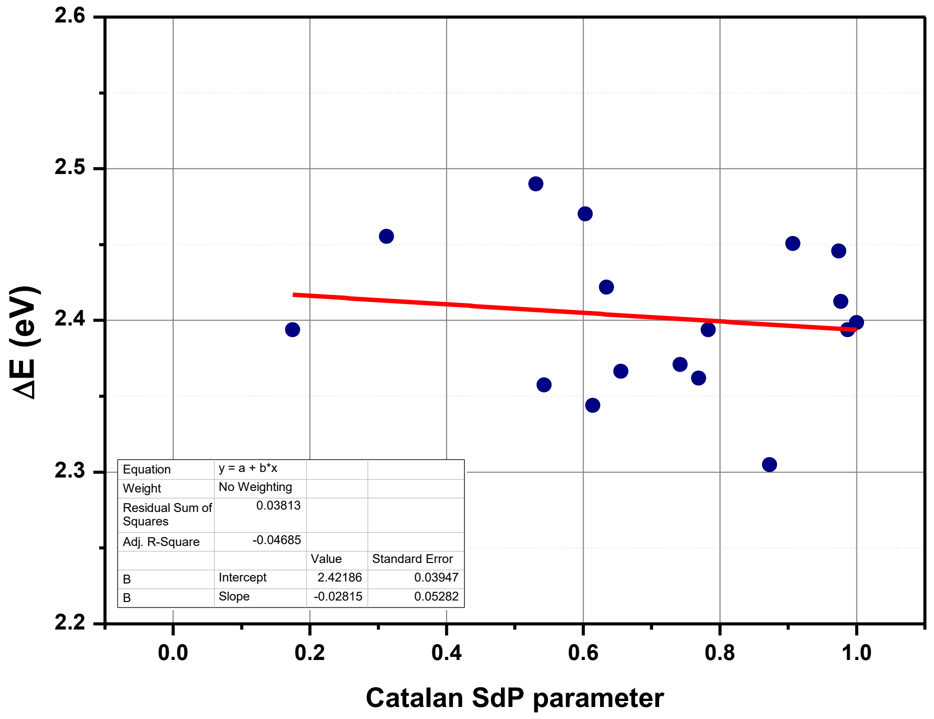

y = -0.02815 x + 2.4219 R^2 = -0.047

Figure S62. Dye 15

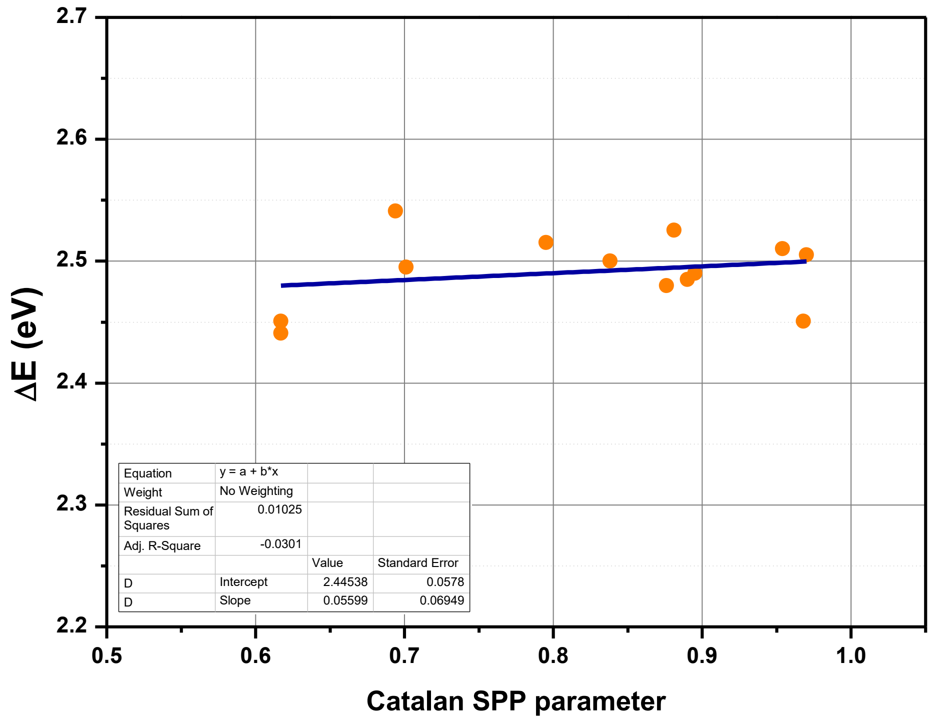

$y = 0.05599\ x + 2.44538 \qquad R^2 = -0.0301$

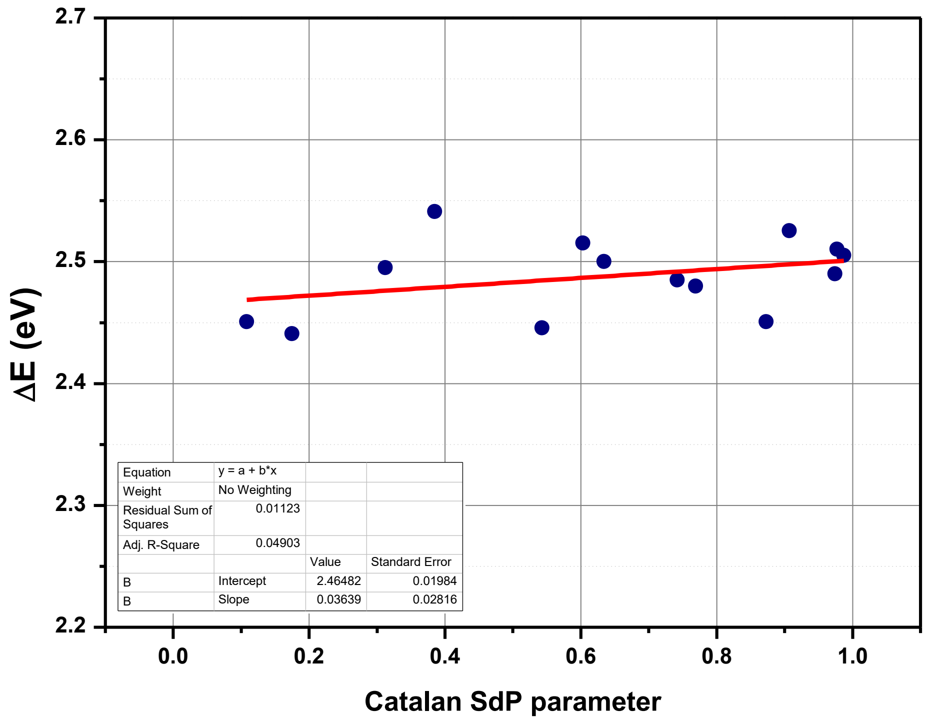

$y = 0.03639\ x + 2.46482 \qquad R^2 = 0.04903$

Table S1. Summary of the optical properties of **Dye 1- Dye 8** in twenty-three solvents, values of the Kamlet and Taft parameters π* and values of the Catalan solvent polarizability (SP), solvent dipolarity (SdP) and solvent polarity/polarizability (SPP) parameters

compounds	π*[1]	SP	SdP	SPP	Dye1	Dye 2	Dye 3	Dye 4	Dye 5	Dye 6	Dye 7	Dye 8
acetone	0.71	0.651	0.907	0.881	370	437	465	-	-	491	-	439
acetonitrile	0.75	0.645	0.974	0.895	370	437	464	-	530	489	-	442
AcOEt	0.54	0.656	0.603	0.795	371	437	471	516	527	493	-	446
anisole	0.73	0.82	0.543	0.854	376	453	482	537	554	505	565	463
butanol	0.47	0.674	0.655	0.837	373	446	482	-	589	504	-	-
chloroform	0.78	0.783	0.614	0.786	376	459	488	544	571	512	580	486
cyclohexane	0.00	0.683	0	0.557	369	446	479	-	-	498	-	-
1,2-dichloroethane	0.81	0.771	0.742	0.890	374	450	477	531	550	501	562	471
dichloromethane	0.82	0.761	0.769	0.876	374	453	480	533	554	504	-	478
diethyl carbonate	0.45	0.653	0.531	0.711	-	437	472	520	535	495	-	454
diethyl ether	0.27	0.617	0.385	0.694	369	443	472	-	-	493	-	-
diglyme	0.64	-	-	0.777	373	440	474	525	542	497	551	445
1,4-dioxane	0.55	0.737	0.312	0.701	372	435	474	523	543	495	549	443
dimethylacetamide	0.88	0.763	0.987	0.970	373	441	472	-	578	498	-	439
DMF	0.87	0.759	0.977	0.954	374	441	472	-	553	497	-	438
DMSO	1.00	0.83	1	1.000	375	441	473	-	534	498	-	432
ethanol	0.54	0.633	0.783	0.853	371	442	477	-	-	500	-	-
heptane	-0.08	0.635	0	0.526	369	444	477	-	-	495	-	-
nitrobenzene	1.01	0.891	0.873	0.968	-	456	487	-	567	511	565	-
THF	0.58	0.714	0.634	0.838	371	439	472	520	534	494	-	439
toluene	0.54	0.66	0.108	0.617	375	451	483	542	562	505	566	472
triethylamine	0.14	0.782	0.284	0.655	370	445	-	-	-	-	-	462
p-xylene	0.43	0.778	0.175	0.617	375	451	483	540	562	505	565	471

[1] Kamlet and Taft parameters [2] Position of the ICT bands are given in nm.

Table S2. Summary of the optical properties of **Dye 9- Dye 15** in twenty-three solvents, values of the Kamlet and Taft parameters *π** and values of the Catalan solvent polarizability (SP), solvent dipolarity (SdP) and solvent polarity/polarizability (SPP) parameters

compounds	π*[1]	SP	SdP	SPP	Dye 9	Dye 10	Dye 11	Dye 12	Dye 13	Dye 14	Dye 15
acetone	0.71	0.651	0.907	0.881	461	439	471	448	450	506	491
acetonitrile	0.75	0.645	0.974	0.895	453	436	471	445	451	507	498
AcOEt	0.54	0.656	0.603	0.795	468	443	474	449	452	502	493
anisole	0.73	0.82	0.543	0.854	498	454	495	463	465	526	507
butanol	0.47	0.674	0.655	0.837	-	459	493	458	460	524	-
chloroform	0.78	0.783	0.614	0.786	-	459	504	463	464	529	-
cyclohexane	0.00	0.683	0	0.557	-	466	493	435	459	-	-
1,2-dichloroethane	0.81	0.771	0.742	0.890	503	454	489	460	461	523	499
dichloromethane	0.82	0.761	0.769	0.876	489	459	492	460	461	525	500
diethyl carbonate	0.45	0.653	0.531	0.711	473	443	476	444	453	498	
diethyl ether	0.27	0.617	0.385	0.694	-	446	485	454	456	-	488
diglyme	0.64			0.777	477	448	485	455	457	515	497
1,4-dioxane	0.55	0.737	0.312	0.701	474	444	476	444	446	505	497
dimethylacetamide	0.88	0.763	0.987	0.970	465	442	476	458	456	518	495
dimethylformamide	0.87	0.759	0.977	0.954	458	443	475	456	455	514	494
DMSO	1.00	0.83	1	1.000	470	442	480	458	458	517	-
ethanol	0.54	0.633	0.783	0.853	-	453	485	455	454	518	-
heptane	-0.08	0.635	0	0.526	-	464	491	456	460	-	-
nitrobenzene	1.01	0.891	0.873	0.968	504	443	499	458	469	538	506
THF	0.58	0.714	0.634	0.838	468	444	475	452	454	512	496
toluene	0.54	0.66	0.108	0.617	500	464	497	465	465	-	506
triethylamine	0.14	0.782	0.284	0.655	513	497	-	459	460	-	-
p-xylene	0.43	0.778	0.175	0.617	501	462	498	465	467	518	508

[1] Kamlet and Taft parameters [2] Position of the ICT bands are given in nm.

Results of the linear correlation analyses

The position of the UV/Vis absorption maxima with regard to the dipolarity/polarizability π^* can be interpreted using a simplified version of the Kamlet–Taft equation :

$$\nu_{max}\ (cm^{-1}) = \nu_{max,0}\ (cm^{-1}) + s\pi^*$$

Table S3. Solvent-independent correlation coefficient *s* of the Kamlet-Taft parameters π^*

Compounds	$\nu_{max,0}$	*s*	R^2
**Dye 1**	3.35008	-0.03692	0.22545
**Dye 2**	2.7973	-0.01227	-0.0400
**Dye 3**	2.59865	0.00997	-0.0420
**Dye 4**	2.33927	0.00117	-0.1110
**Dye 5**	2.24973	-0.00211	-0.0667
**Dye 6**	2.49705	0.02078	-0.0064
**Dye 8**	2.64729	0.13459	0.014
**Dye 9**	2.54281	0.0708	-0.037
**Dye 10**	2.69011	0.11027	0.308
**Dye 11**	2.53375	0.0369	-0.0054
**Dye 12**	2.75291	0.04384	0.03286
**Dye 13**	2.708	0.1633	-0.0476
**Dye 14**	2.49421	-0.13187	0.205
**Dye 15**	2.46513	-0.02926	-0.0242

The position of the UV/Vis absorption maxima with regard to the dipolarity/polarizability π^* can also be interpreted using a Catalan parameters, namely, the solvent dipolarity (SdP) and the solvent polarity/polarizability (SPP) using the following equations :

$$\nu_{max}\,(cm^{-1}) = \nu_{max,01}\,(cm^{-1}) + a \times SdP$$
$$\nu_{max}\,(cm^{-1}) = \nu_{max,02}\,(cm^{-1}) + b \times SPP$$

Table S4. Solvent-independent correlation coefficients a and b of the Catalan parameters SdP and SPP.

Compounds	$\nu_{max,01}$	*a*	R^2	$\nu_{max,02}$	*b*	R^2
**Dye 1**	3.33669	-0.01269	-0.018	2.49421	-0.04526	0.205
**Dye 2**	2.77528	0.02287	-0.022	2.77265	0.02358	-0.046
**Dye 4**	2.58021	0.03964	0.087	2.55615	0.06182	-0.00851
**Dye 5**	2.23246	0.0204	-0.065	2.24976	-0.0041	-0.077
**Dye 6**	2.48005	0.00726	-0.046	2.49292	-0.0887	-0.054
**Dye 8**	2.61092	0.1867	0.2189	2.37488	0.44064	0.2003
**Dye 9**	2.4746	0.174	0.2266	2.37423	0.26922	0.0570
**Dye 10**	2.6798	0.1241	0.4643	2.5419	0.26716	0.3828
**Dye 11**	2.5105	0.0748	0.1558	2.4477	0.1379	0.0789
**Dye 12**	2.7347	-0.01245	-0.0423	2.7723	-0.0538	-0.0249
**Dye 13**	2.69297	0.02547	0.0089	2.6829	0.03403	-0.032
**Dye 14**	2.4219	-0.02815	-0.047	2.4981	-0.10193	-0.016
**Dye 15**	2.46482	0.03639	0.0490	2.44538	0.05599	-0.0301

Optimized geometries and HOMO LUMO electronic distribution of all compounds

Figure S63. Dye 1

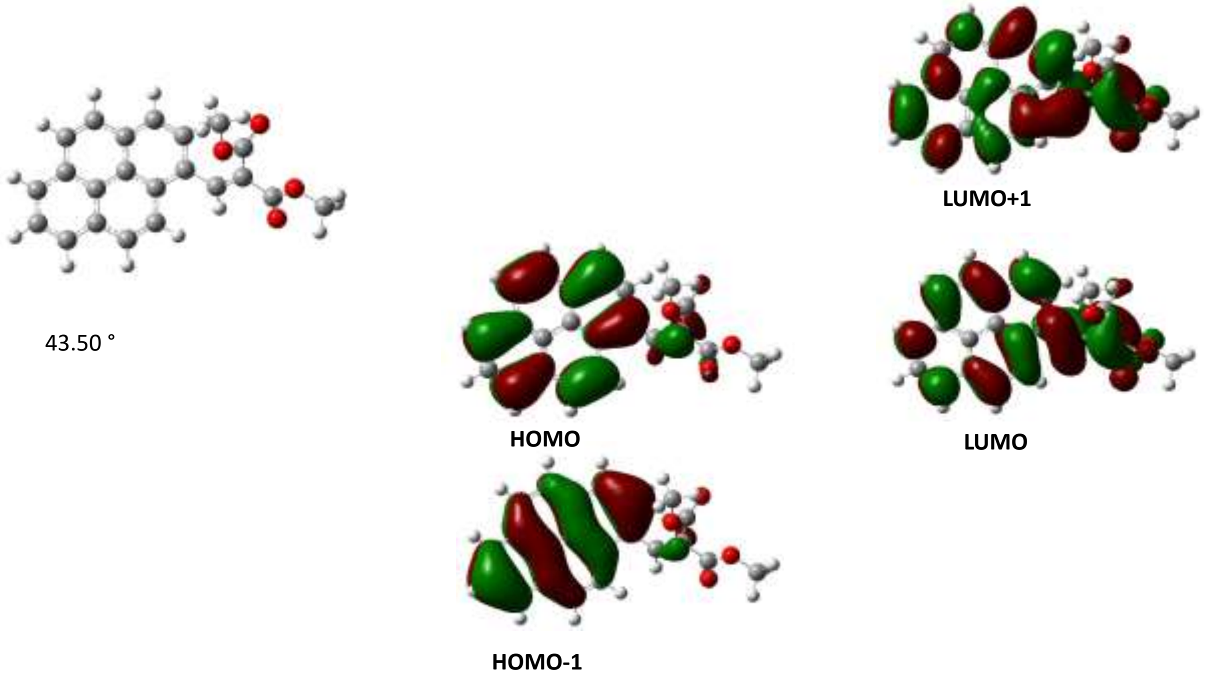

Figure S64. Dye 2

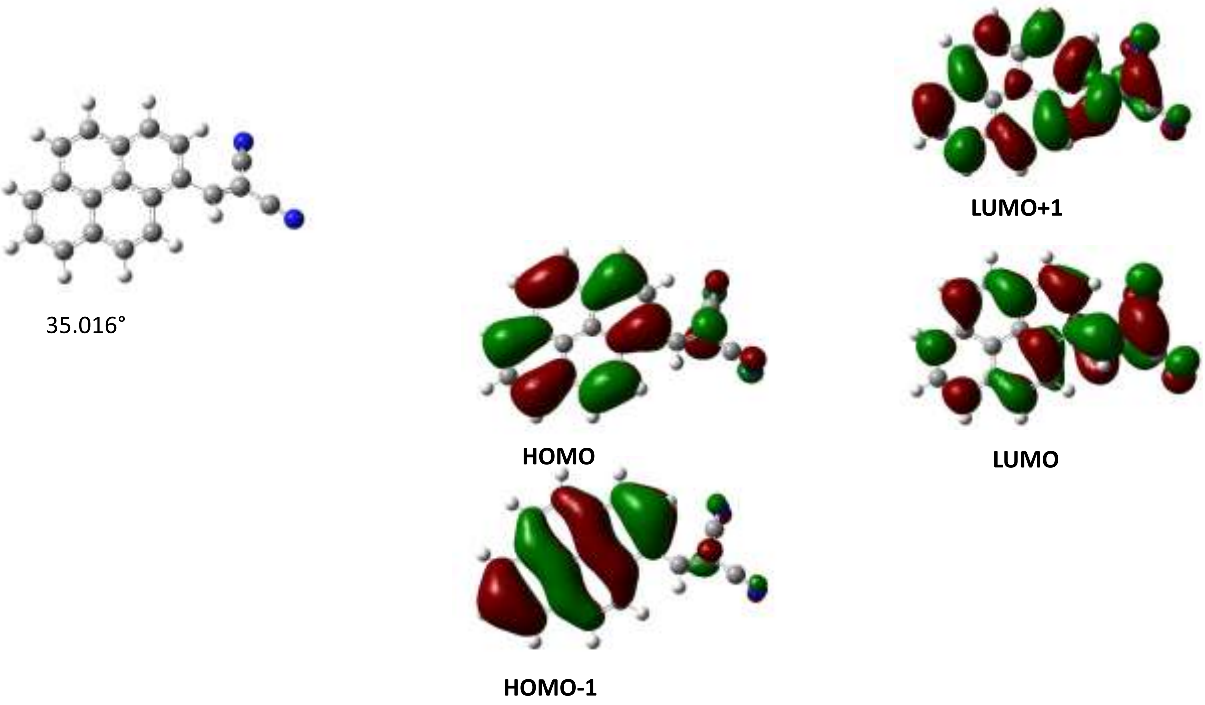

Figure S65. Dye 3

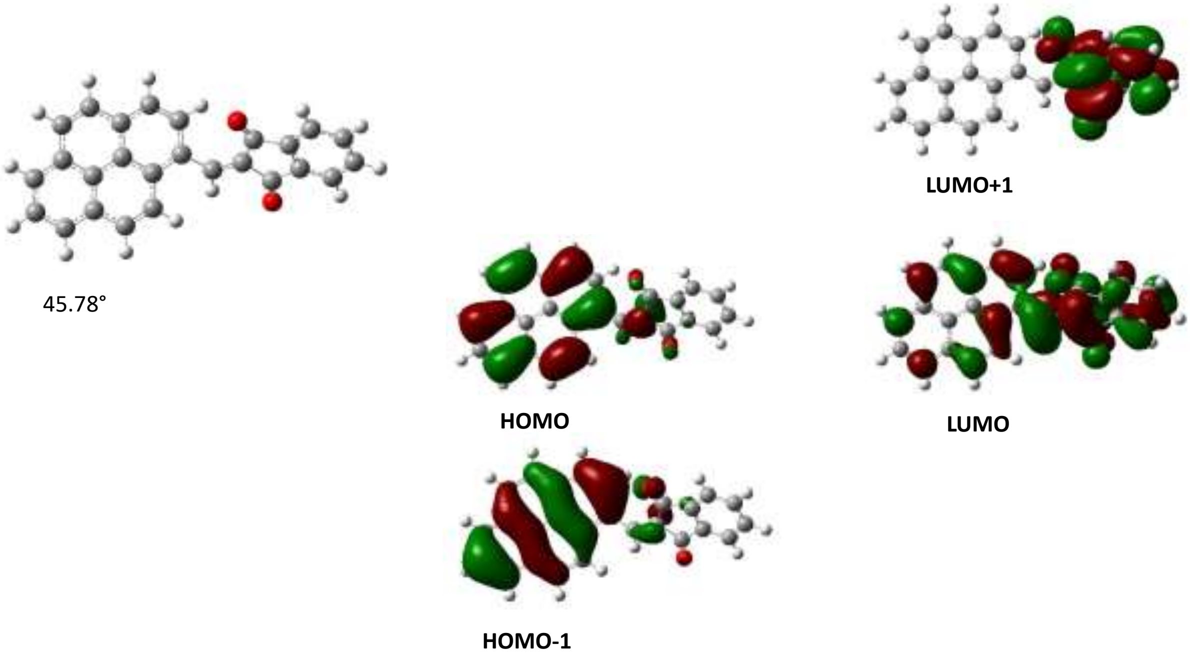

Figure S66. Dye 4

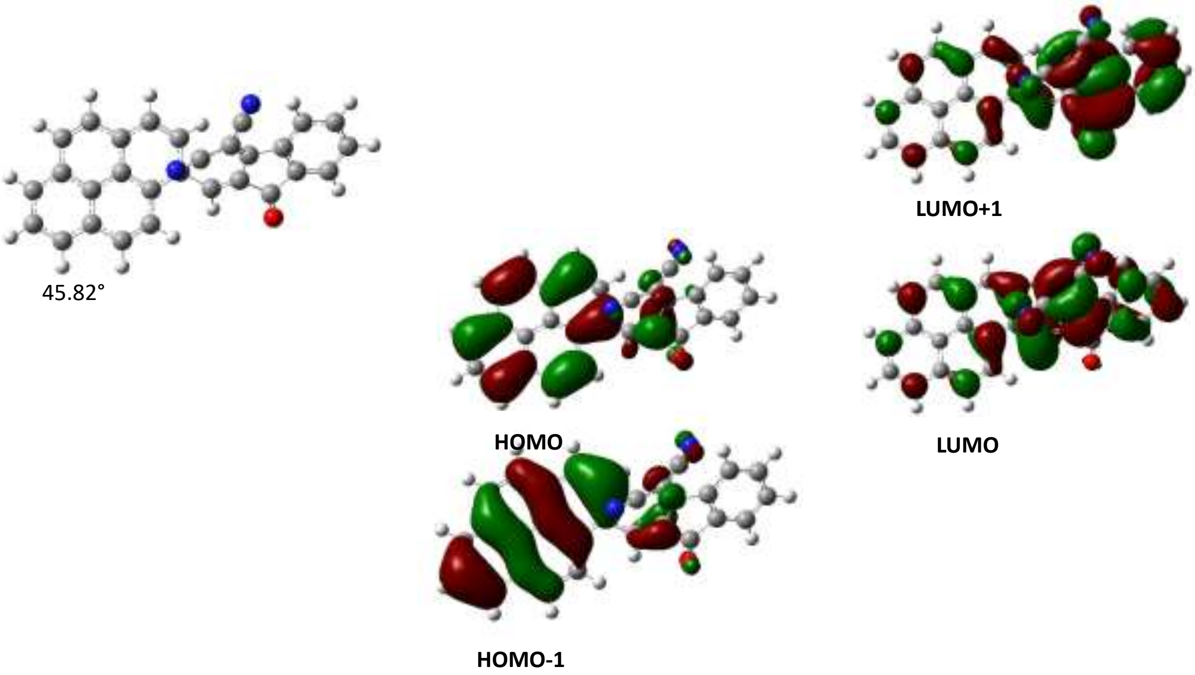

Figure S67. **Dye 5**

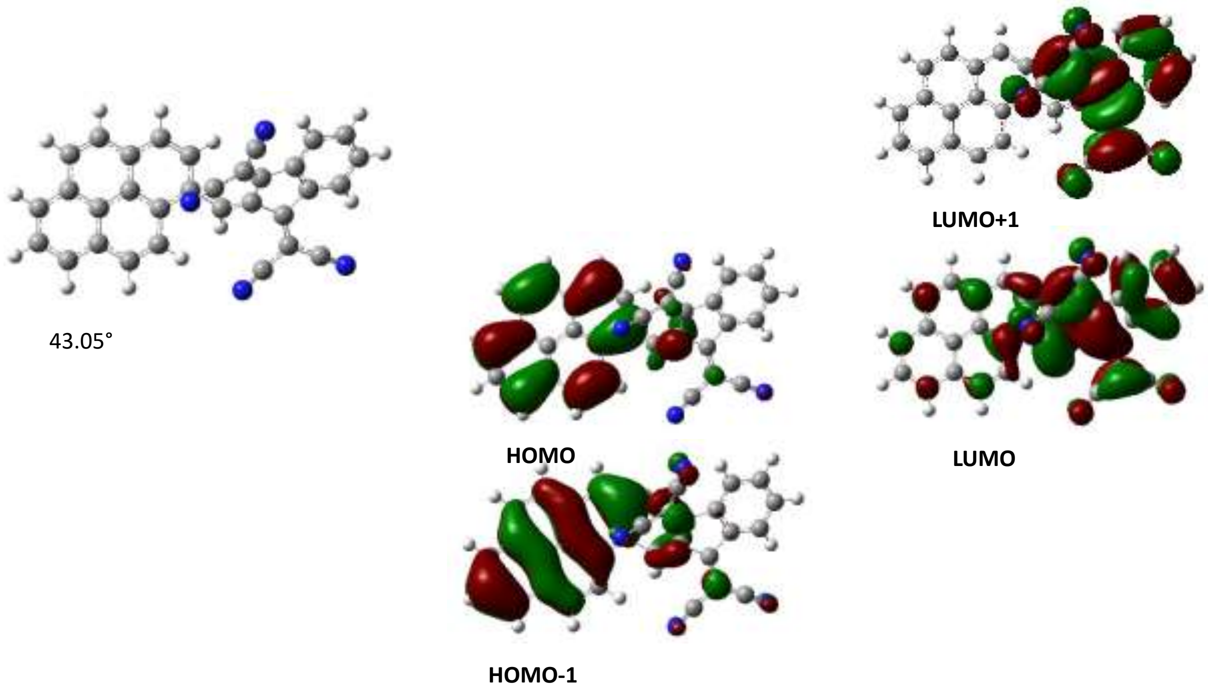

Figure S68. **Dye 6**

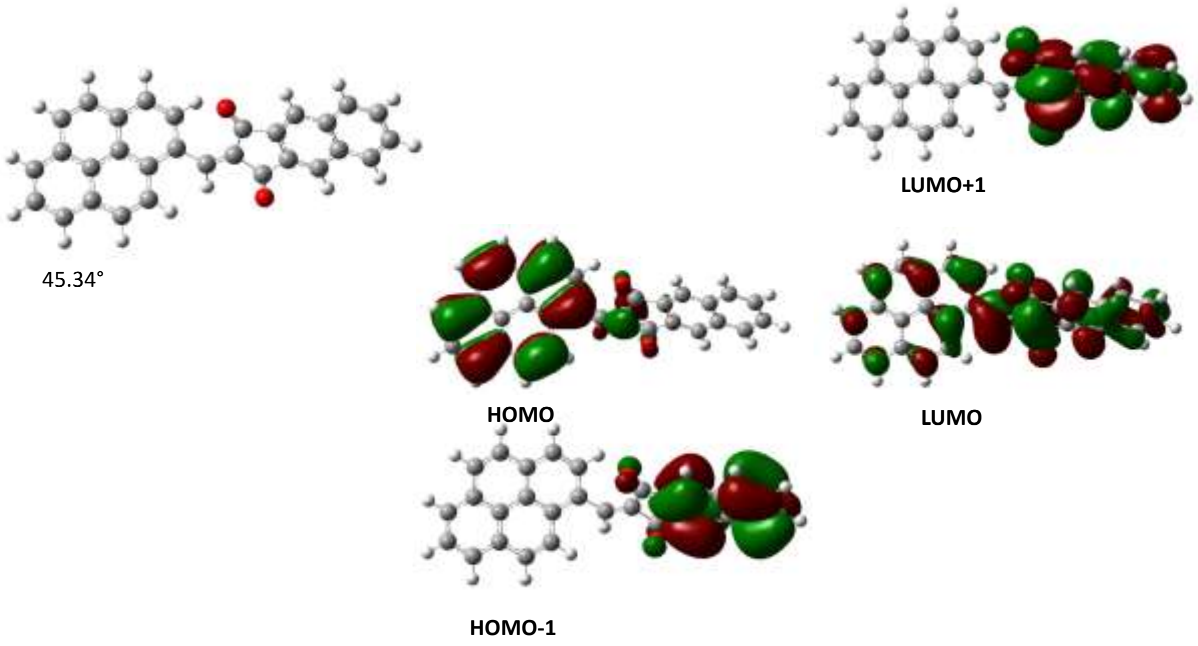

Figure S69. Dye 7

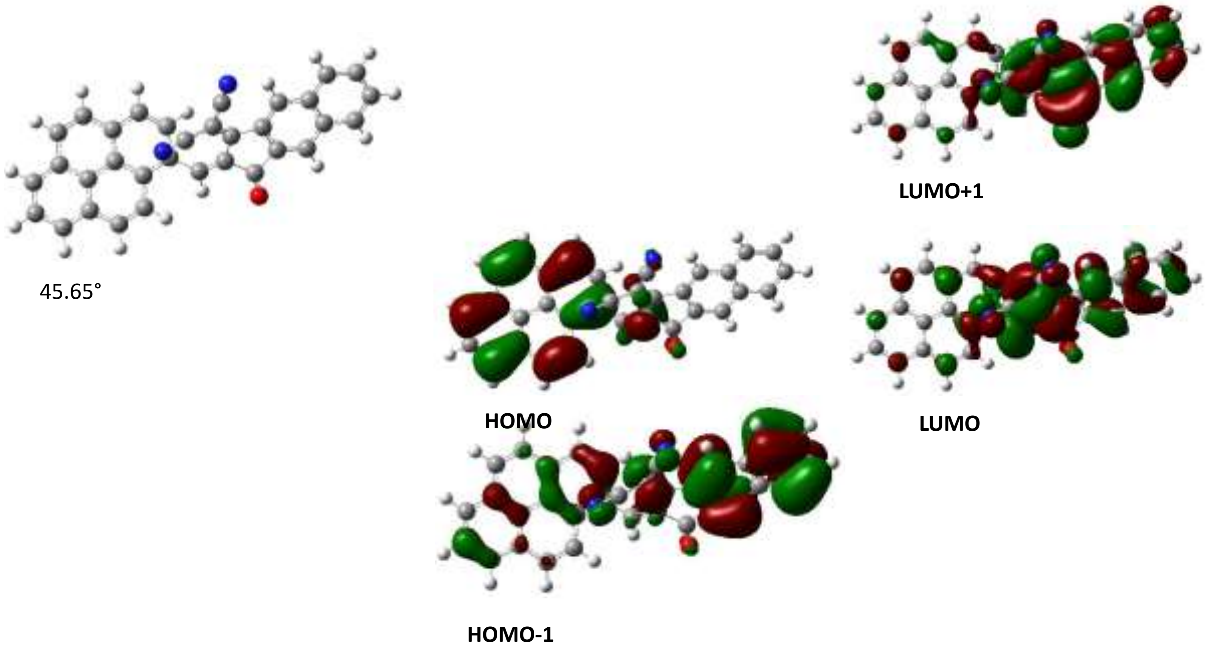

Figure S70. Dye 8

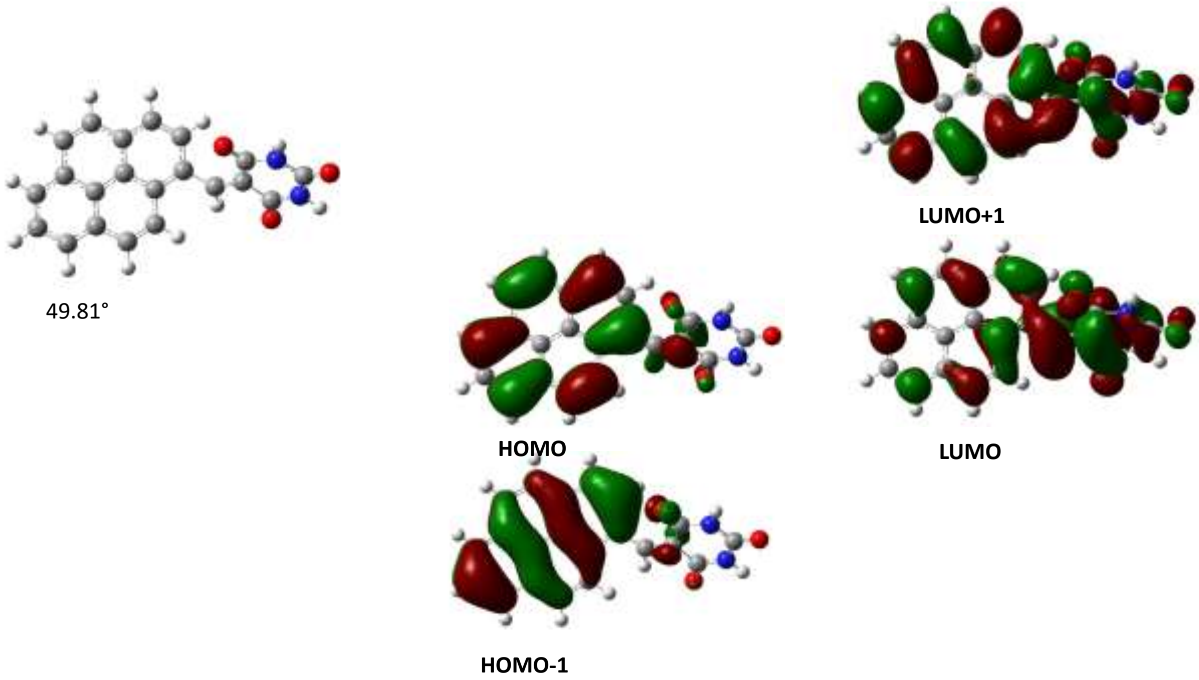

Figure S71. Dye 9

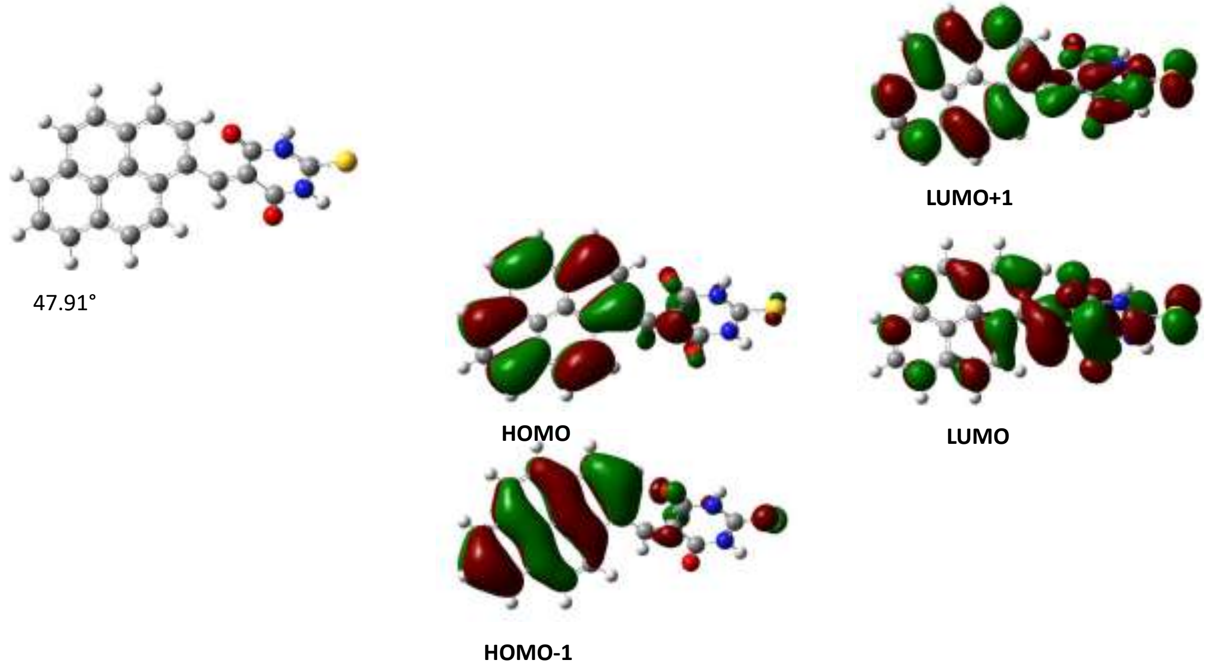

Figure S72. Dye 10

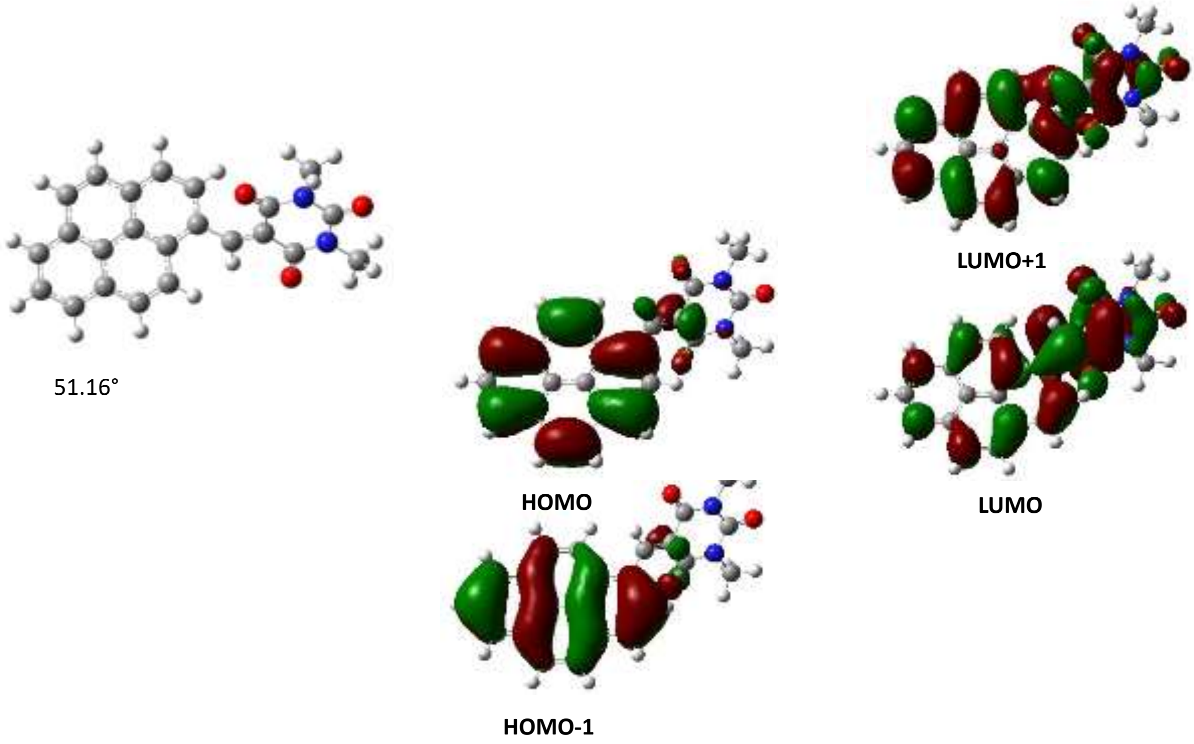

Figure S73. Dye 11

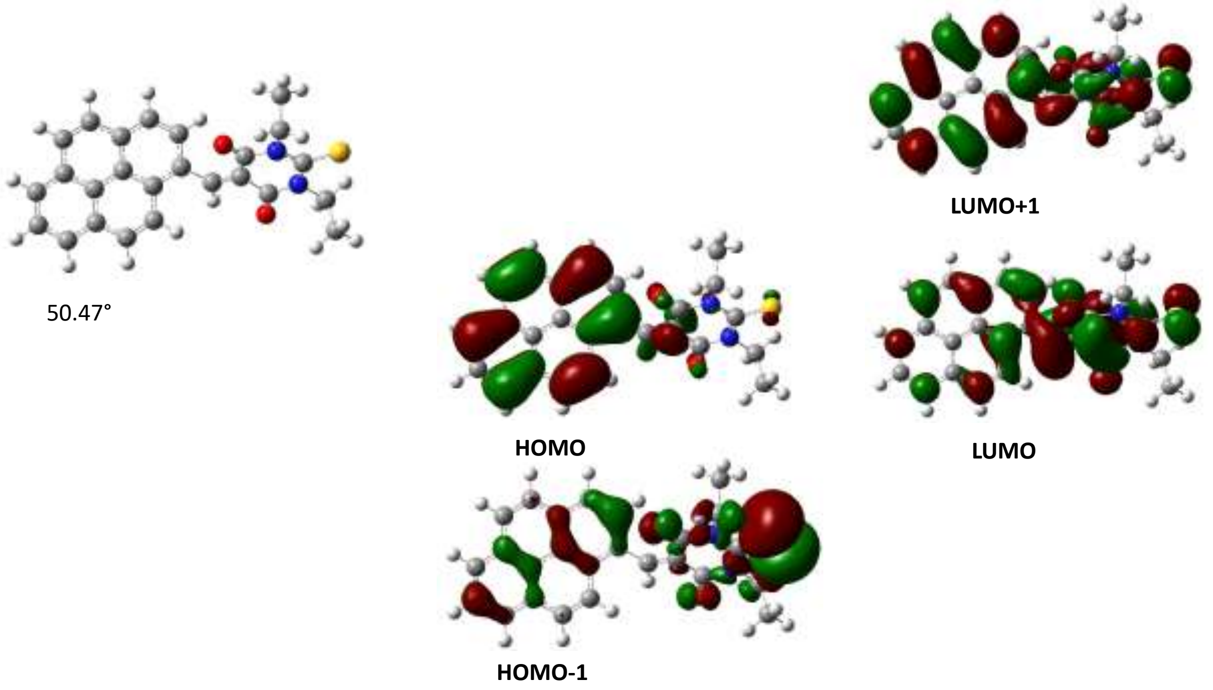

Figure S74. Dye 12

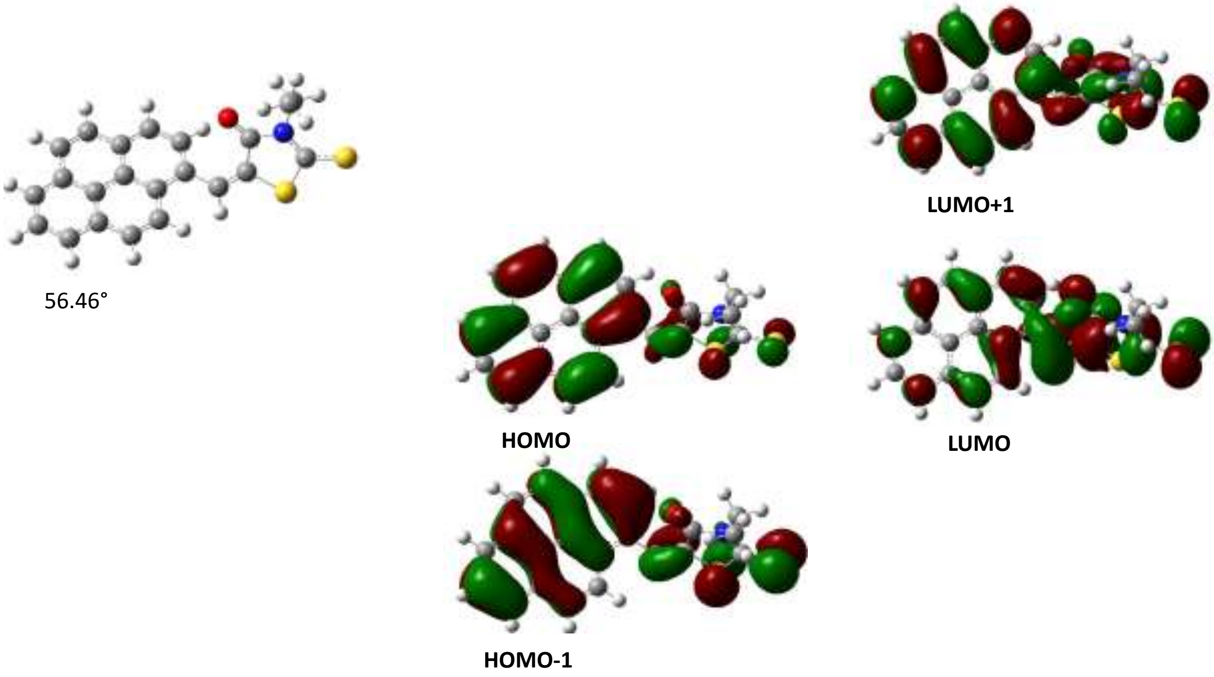

Figure S75. **Dye 13**

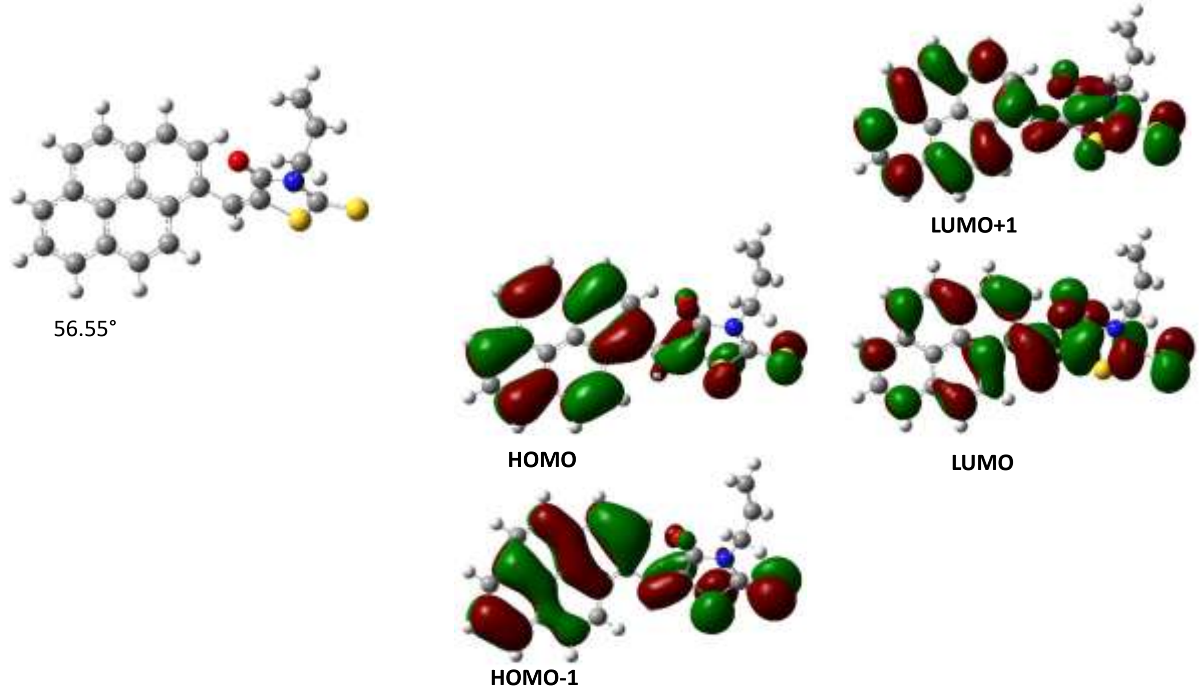

Figure S76. **Dye 14**

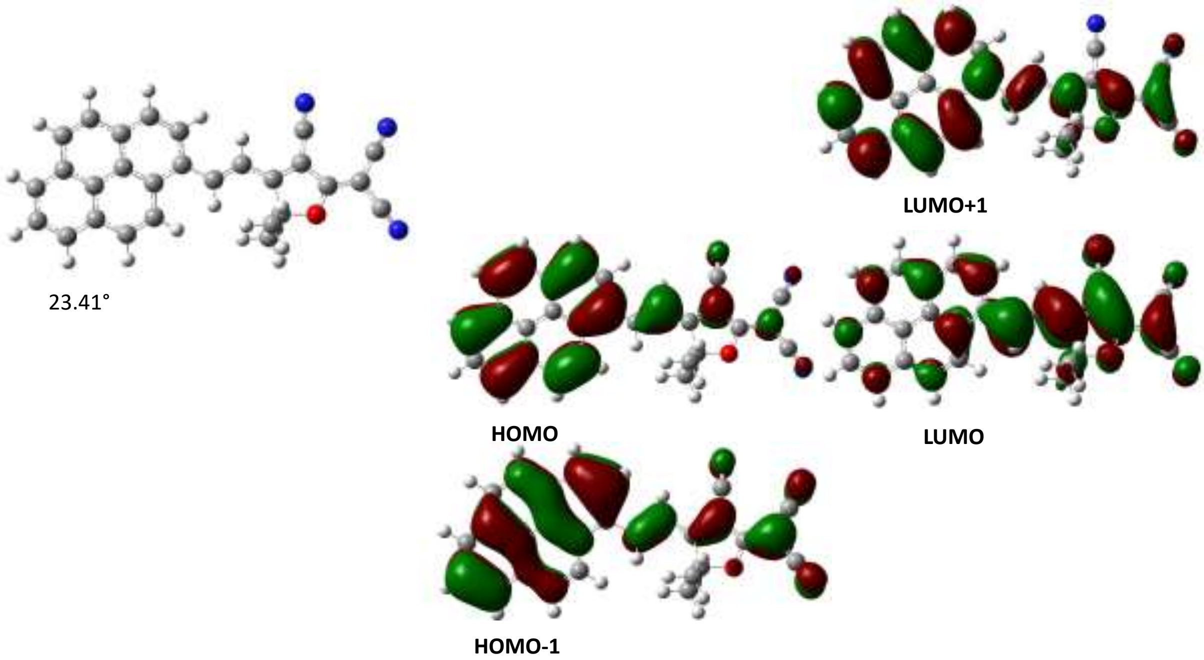

Figure S77. Dye 15

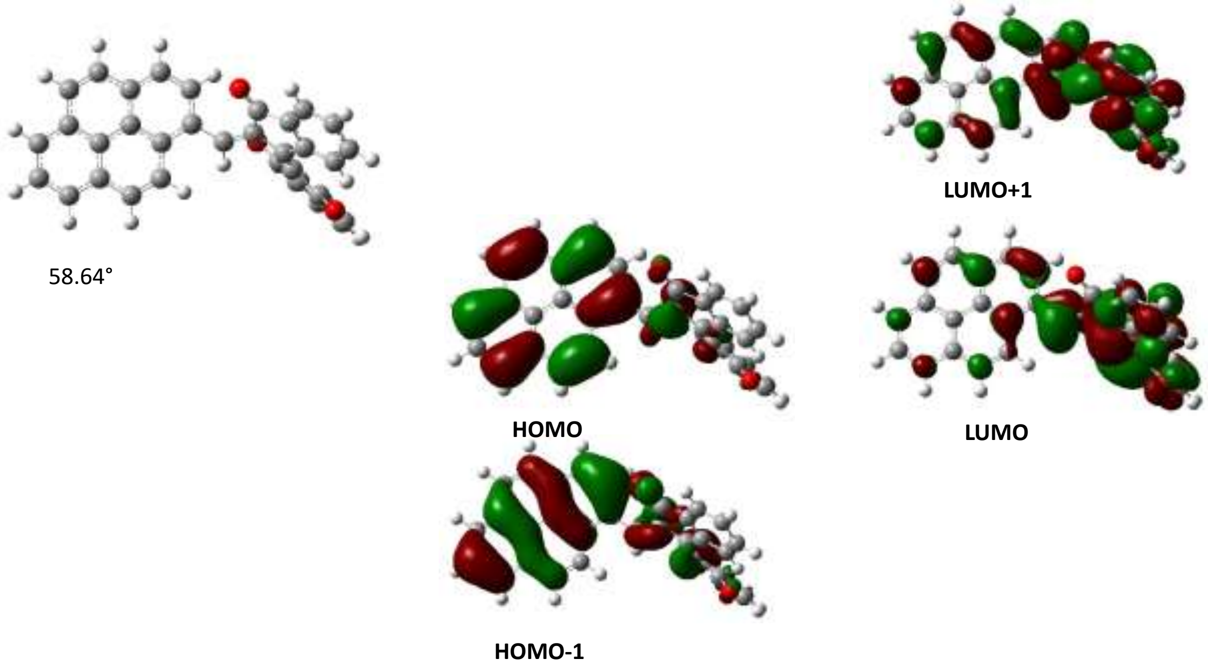

Table S5. Energy levels of the main orbitals for dyes Dye 1-Dye 15

	HOMO -1	HOMO	LUMO	LUMO +1
**Dye 1**	-8.658	-7.563	-1.069	0.128
**Dye 2**	-8.805	-7.712	-1.705	-0.106
**Dye 3**	-8.614	-7.605	-1.445	-0.657
**Dye 4**	-8.656	-7.623	-1.784	-1.119
**Dye 5**	-8.654	-7.647	-1.829	-1.773
**Dye 6**	-8.489	-7.582	-1.823	-0.594
**Dye 7**	-8.532	-7.619	-1.905	-1.050
**Dye 8**	-8.683	-7.650	-1.670	-0.142
**Dye 9**	-8.711	-7.676	-1.885	-0.339
**Dye 10**	-8.609	-7.616	-1.318	-0.114
**Dye 11**	-8.519	-7.626	-1.783	-0.298
**Dye 12**	-8.505	-7.480	-1.460	-0.295
**Dye 13**	-8.519	-6.197	-1.260	-0.288
**Dye 14**	-8.619	-7.519	-2.089	-0.408
**Dye 15**	-8.548	-7.572	-1.759	-1.070

Table S6. Main transitions observed for dyes Dye 1-Dye 15

Dye 1

No.	Wavelength nm)	Osc. Strength	Major contribs	
1	352.859359115	0.8672	HOMO->LUMO	94%
2	317.558059094	0.015	H-1->LUMO	47%
3	275.098611046	0.2322	H-1->LUMO	11%
4	260.492883881	0.2565	H-1->LUMO	18%
5	256.600424298	0.0966	H-2->LUMO	51%
6	251.448432328	0.0696	H-2->LUMO	26%
7	240.70861422	0.0056	H-5->LUMO	18%
8	230.415345039	0.0251	H-3->LUMO	47%
9	226.919347363	0.9841	H-1->L+1	59%
10	220.844290291	0.2207	H-6->LUMO	10%

Dye 2

No.	Wavelength nm)	Osc. Strength	Major contribs	
1	399.279250973	0.9035	HOMO->LUMO	94%
2	330.589251846	0.0584	H-1->LUMO	69%
3	285.099781577	0.2251	HOMO->L+1	74%
4	274.775482054	0.0531	H-2->LUMO	70%
5	266.374890992	0.1056	H-2->LUMO	11%
6	253.38577387	0.0658	H-3->LUMO	17%
7	240.2515076	0.1055	H-3->LUMO	59%
8	233.897134418	0.4683	H-1->L+1	69%
9	222.780789916	0.9317	H-4->LUMO	18%
10	219.115285261	0.0096	H-4->LUMO	19%

Dye 3

No.	Wavelength nm)	Osc. Strength	Major contribs	
1	405.442096181	1.0228	HOMO->LUMO	87%
2	358.916723634	0.0331	H-3->LUMO	15%
3	335.491376264	0.006	H-7->LUMO	14%
4	327.359647812	0.0595	H-1->LUMO	46%
5	293.648318441	0.0653	HOMO->L+1	38%
6	290.164040844	0.1083	HOMO->L+1	21%
7	274.447036064	0.1274	H-3->LUMO	19%
8	266.598273367	0.0224	H-6->LUMO	14%
9	265.41689254	0.0486	H-4->LUMO	15%
10	256.542020344	0.0577	HOMO->L+3	13%

Dye 4

No.	Wavelength nm)	Osc. Strength	Major contribs
1	437.658205416	0.6231	HOMO->LUMO 83%
2	338.227877383	0.0642	H-1->LUMO (59%), HOMO->L+3 (12%)
3	333.595740764	0.1891	HOMO->L+1 (58%)
4	326.721284421	0.0347	H-6->L+1 (49%)
5	311.494593403	0.4582	H-2->LUMO (64%)
6	291.617727473	0.0765	H-6->LUMO (17%), HOMO->L+2 (38%)

7	289.865553064	0.2994	H-3->LUMO (12%), H-2->L (12%), HOMO->L+2 (21%)
8	285.001478087	0.018	H-6->LUMO (15%), H-5->L (26%), H-2->L+1 (20%)
9	284.877057608 (14%)	0.0264	H-6->L (22%), H-5->L (11%), H-4->L (11%), H->L+3
10	268.259537436	0.0335	H-3->L (40%), H-3->L+1 (11%), H-3->L+2 (12%)

Dye 5

No.	Wavelength nm)	Osc. Strength	Major contribs	Minor Contribs
1	439.115257702	0.6112	HOMO->LUMO	84%
2	373.064310683	0.1464	HOMO->L+1	77%
3	340.074038653	0.0526	H-1->LUMO	65%
4	324.396109399	0.6142	H-2->L+1	65%
5	314.38545785	0.2221	H-2->LUMO	65%
6	305.11675406	0.2218	H-4->L+1	11%
7	295.665076101	0.0392	H-5->L+1	11%
8	288.107526635	0.1383	H-4->L+1	36%
9	280.070010645	0.1695	H-1->L+1	14%
10	272.283283216	0.0717	H-4->LUMO	69%

Dye 6

No.	Wavelength nm)	Osc. Strength	Major contribs	
1	418.243803172	1.1726	HOMO->LUMO	84%
2	370.378470537	0.0364	H-4->LUMO	58%
3	336.995985464	0.0173	H-7->LUMO	17%
4	327.351004653	0.0822	H-7->LUMO	14%
5	325.571642803	0.0179	H-1->LUMO	79%
6	306.883970724	0.0265	H-3->LUMO	23%
7	293.544032512	0.177	HOMO->L+2	54%
8	278.873103336	0.1554	H-3->L+1	11%
9	277.624203435	0.1417	H-5->LUMO	13%
10	266.988658022	0.0452	H-5->LUMO	32%

Dye 7

No.	Wavelength nm)	Osc. Strength	Major contribs	
1	450.017033909	0.7104	HOMO->LUMO	84%
2	357.777436983	0.2288	H-1->LUMO	83%
3	339.738568017	0.0291	H-2->LUMO	62%
4	333.28188224	0.018	H-6->LUMO	18%
5	326.669634326	0.2569	H-3->LUMO	13%
6	319.119203676	0.2504	H-3->LUMO	26%
7	302.385720239	0.2817	H-4->LUMO	10%
8	293.627455328	0.4408	H-3->LUMO	12%
9	289.845223986	0.2945	HOMO->L+2	23%
10	282.147766453	0.1214	H-6->LUMO	20%

Dye 8

No.	Wavelength nm)	Osc. Strength	Major contribs	
1	408.730114763	0.8157	HOMO->LUMO	91%

2	335.400619521	0.0413	H-1->LUMO	70%
3	299.449794735	0.0756	H-4->LUMO	36%
4	288.617237796	0.1115	H-4->LUMO	27%
5	275.593920628	0.0843	H-2->LUMO	31%
6	266.707236458	0.0573	H-2->LUMO	39%
7	256.733259504	0.0514	HOMO->L+2	11%
8	251.775227464	0.1434	H-8->LUMO	38%
9	241.807140095	0.1439	H-3->LUMO	55%
10	236.909452769	0.3655	H-1->L+1	62%

Dye 9

No.	Wavelength nm)	Osc. Strength	Major contribs	
1	397.984762341	0.8086	HOMO->LUMO	90%
2	329.789049108	0.0271	H-1->LUMO	64%
3	300.560454322	0.0896	H-4->LUMO	36%
4	290.069000801	0.1111	H-4->LUMO	26%
5	273.502587603	0.1019	H-2->LUMO	17%
6	264.003988272	0.0361	H-2->LUMO	44%
7	257.709817111	0.2031	H-8->LUMO	22%
8	256.144519073	0.047	H-2->LUMO	11%
9	249.319698792	0.0416	H-8->LUMO	21%
10	240.256163186	0.1505	H-3->LUMO	35%

Dye 10

No.	Wavelength nm)	Osc. Strength	Major contribs	
1	422.995438614	0.9267	HOMO->LUMO	88%
2	382.56099544	0.0108	H-1->LUMO	57%
3	337.748761917	0.0418	H-2->LUMO	59%
4	302.858451835	0.0644	H-7->LUMO	21%
5	291.528587581	0.0977	H-7->LUMO	31%
6	286.609013182	0.531	H-3->LUMO 69%	
7	279.067689323	0.0573	H-4->LUMO 20%	
8	270.495228668	0.0272	H-6->LUMO 58%	
9	267.334065747	0.0384	H-4->LUMO 40%	
10	263.431834723	0.1389	H-9->LUMO 35%	

Dye 11

No.	Wavelength nm)	Osc. Strength	Major contribs	
1	422.995438614	0.9267	HOMO->LUMO	88%
2	382.56099544	0.0108	H-1->LUMO	57%
3	337.748761917	0.0418	H-2->LUMO	59%
4	302.858451835	0.0644	H-7->LUMO	21%
5	291.528587581	0.0977	H-7->LUMO	31%
6	286.609013182	0.531	H-3->LUMO	(69%
7	279.067689323	0.0573	H-4->LUMO	20%
8	270.495228668	0.0272	H-6->LUMO	58%
9	267.334065747	0.0384	H-4->LUMO	40%
10	263.431834723	0.1389	H-9->LUMO	35%

Dye 12

No.	Wavelength nm)	Osc. Strength	Major contribs	
1	392.777650042	1.0089	HOMO->LUMO	82%
2	369.14339778	0.0213	H-3->LUMO	39%
3	324.787009515	0.0344	H-1->LUMO	52%
4	309.210646712	0.1023	H-2->LUMO	23%
5	290.225170909	0.2638	H-7->LUMO	15%
6	279.5837122	0.0007	H-7->LUMO	23%
7	264.285364424	0.1726	H-7->LUMO	12%
8	261.99009596	0.1807	H-5->LUMO	14%
9	261.487278313	0.2322	HOMO->L+2	22%
10	253.261552471	0.0134	H-4->LUMO	32%

Dye 13

No.	Wavelength (nm)	Osc. Strength	Major contribs
1	393.213640583	1.0117	HOMO->LUMO (82%)
2	368.594681488	0.0217	H-3->LUMO (40%), H-3->L+1 (16%), H-2->LUMO (17%)
3	324.940226995	0.0363	H-1->LUMO (53%), HOMO->L+2 (23%)
4	308.817856462	0.1082	H-2->LUMO (23%), HOMO->L+1 (41%)
5	290.469948956	0.2359	H-8->LUMO (12%), HOMO->L+1 (24%)
6	279.356930765	0.0077	H-8->LUMO (20%), HOMO->L+1 (15%)
7	265.553327362	0.1131	H-6->LUMO (14%), H-5->LUMO (26%)
8	261.779893189	0.0067	H-4->LUMO (16%), H-4->L+1 (12%), HOMO->L+4 (58%)
9	260.460050024	0.4683	H-5->LUMO (27%), HOMO->L+2 (25%)
10	253.344353199	0.0073	H-4->LUMO (30%), HOMO->L+4 (26%)

Dye 14

No.	Wavelength (nm)	Osc. Strength	Major contribs	
1	450.73687793	1.6471	HOMO->LUMO	88%
2	339.041792262	0.1143	H-1->LUMO	65%
3	313.43173904	0.1466	H-2->LUMO	51%
4	291.04953875	0.2683	H-2->LUMO	22%
5	278.472234603	0.0695	H-3->LUMO	32%
6	270.436227833	0.0594	H-3->LUMO	38%
7	262.161827358	0.0525	H-4->LUMO	33%
8	252.261883278	0.0317	H-4->LUMO	33%
9	239.587611378	0.2244	H-1->L+1	53%
10	233.452320722	0.3	H-2->L+1	12%

Dye 15

No.	Wavelength (nm)	Osc. Strength	Major contribs	
1	443.212243556	0.7973	H-2->LUMO	10%
2	385.750888312	0.2691	H-4->LUMO	10%
3	355.898019382	0.0026	H-8->L+1	13%
4	347.246024401	0.0211	H-4->L+2	11%

5	336.867797887	0.2331	H-1->LUMO	32%
6	330.413050347	0.3542	H-3->LUMO	21%
7	323.254316288	0.2028	H-2->LUMO	11%
8	299.457027298	0.0508	H-8->LUMO	18%
9	295.968568457	0.0404	H-2->L+2	32%
10	291.398404184	0.0202	H-6->LUMO	19%

TGA thermograms of the different dyes

Figure S78. Dye 1

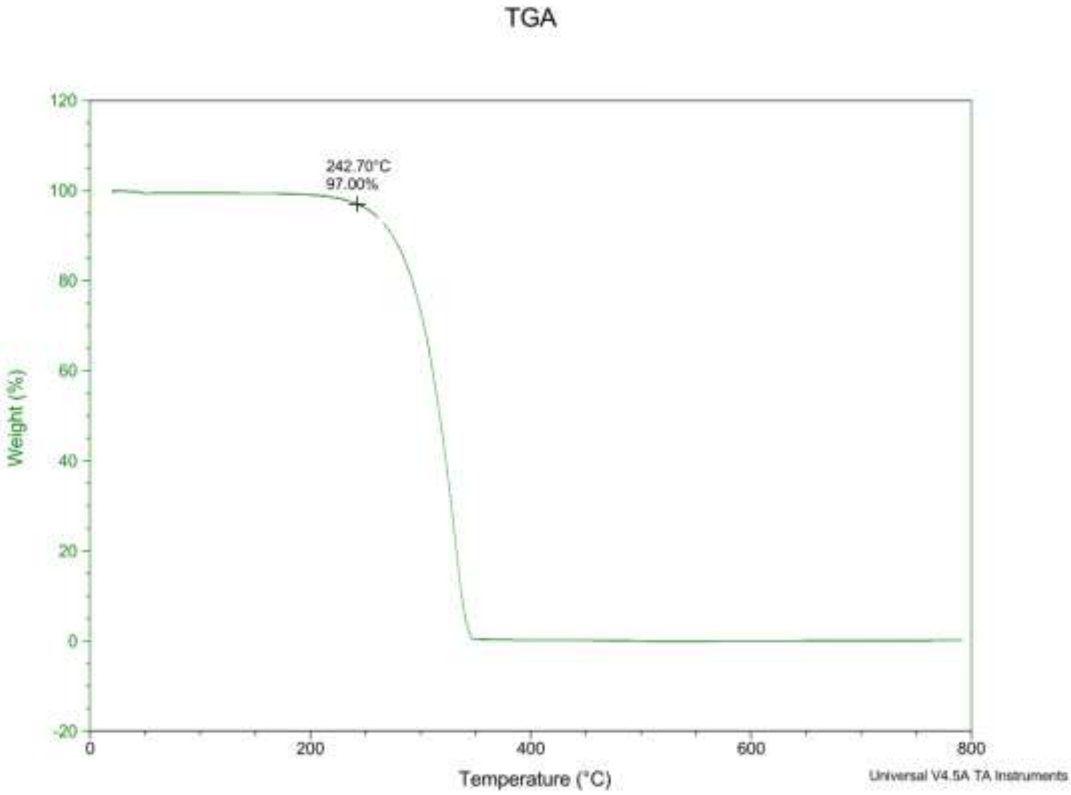

Figure S79. Dve 2

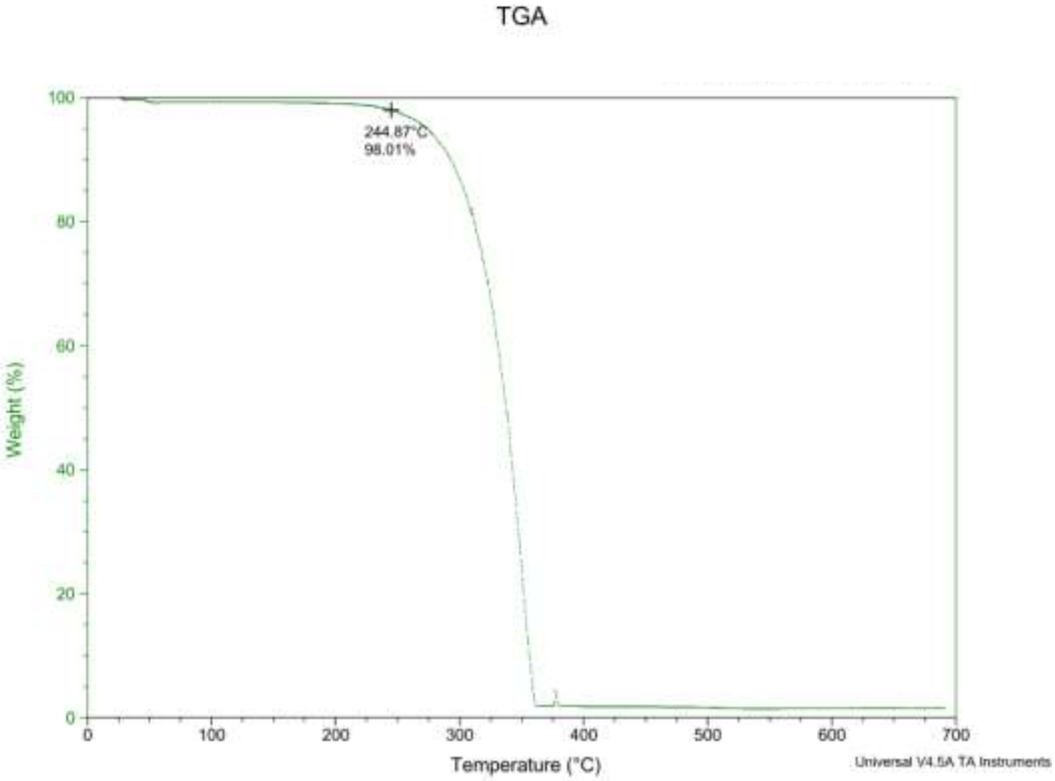

Figure S80. Dye 3

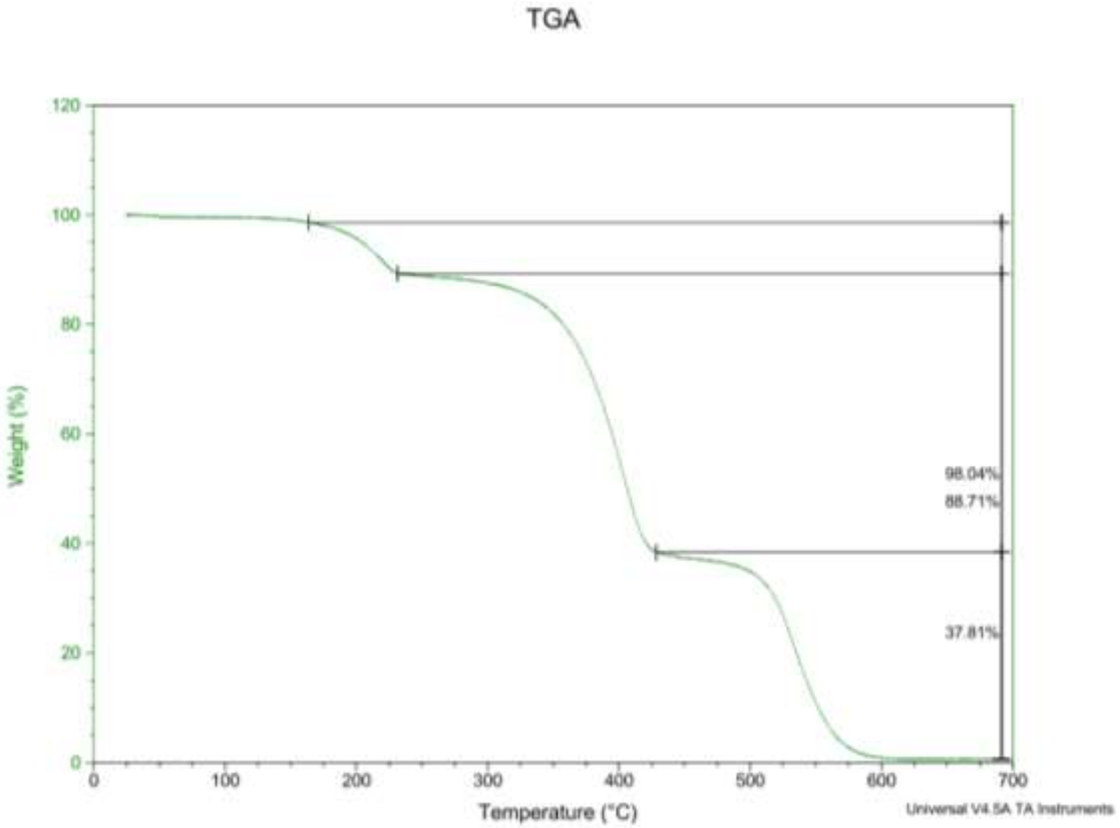

Figure S81. Dye 4

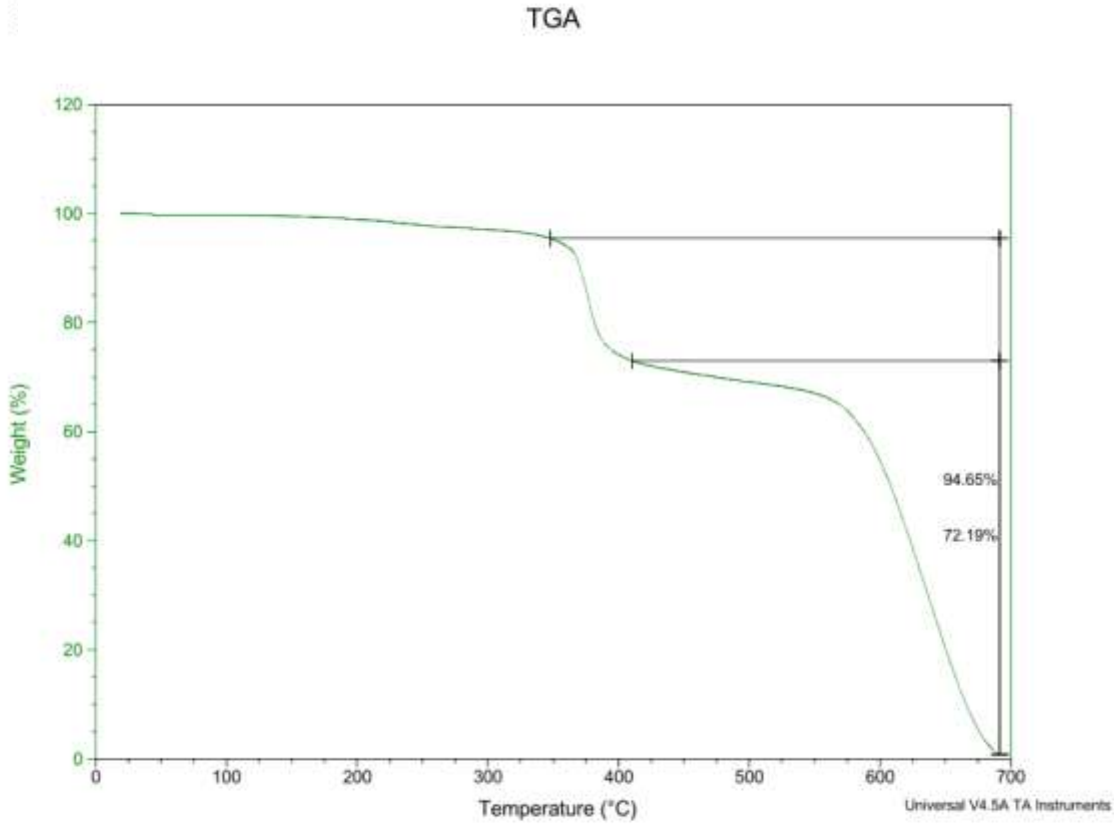

Figure S82. Dye 5

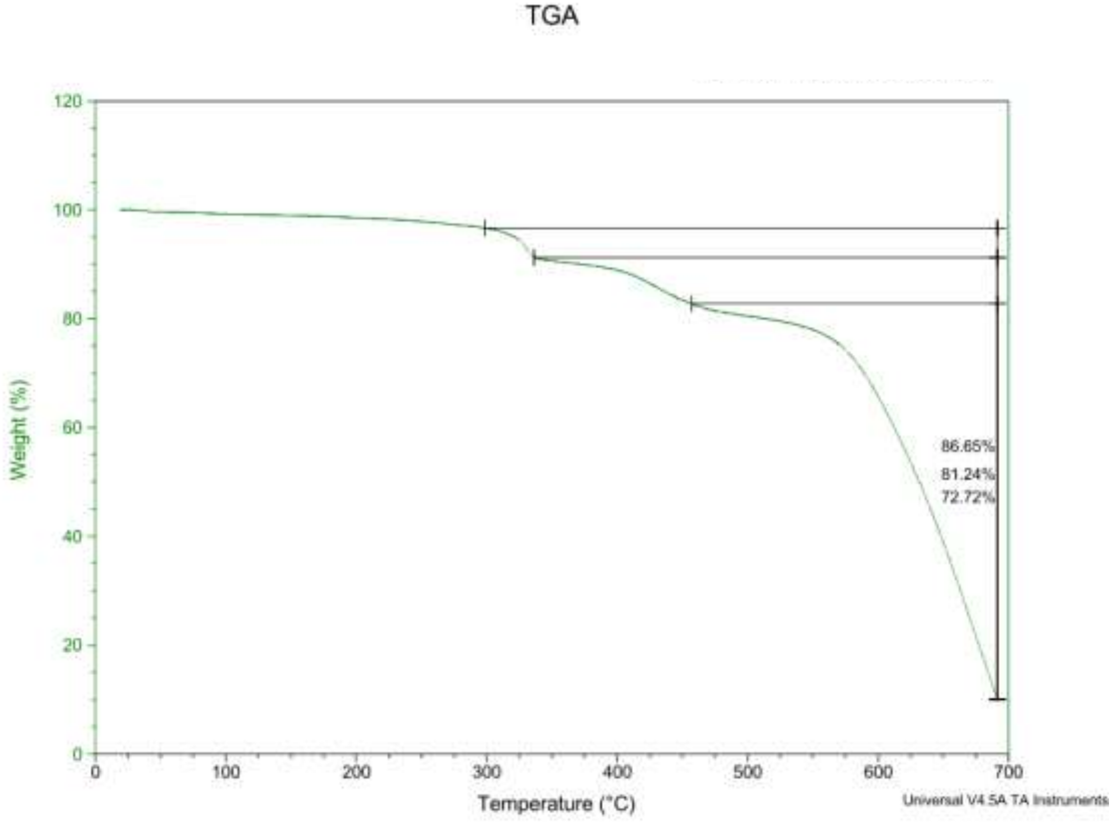

Figure S83. Dye 6

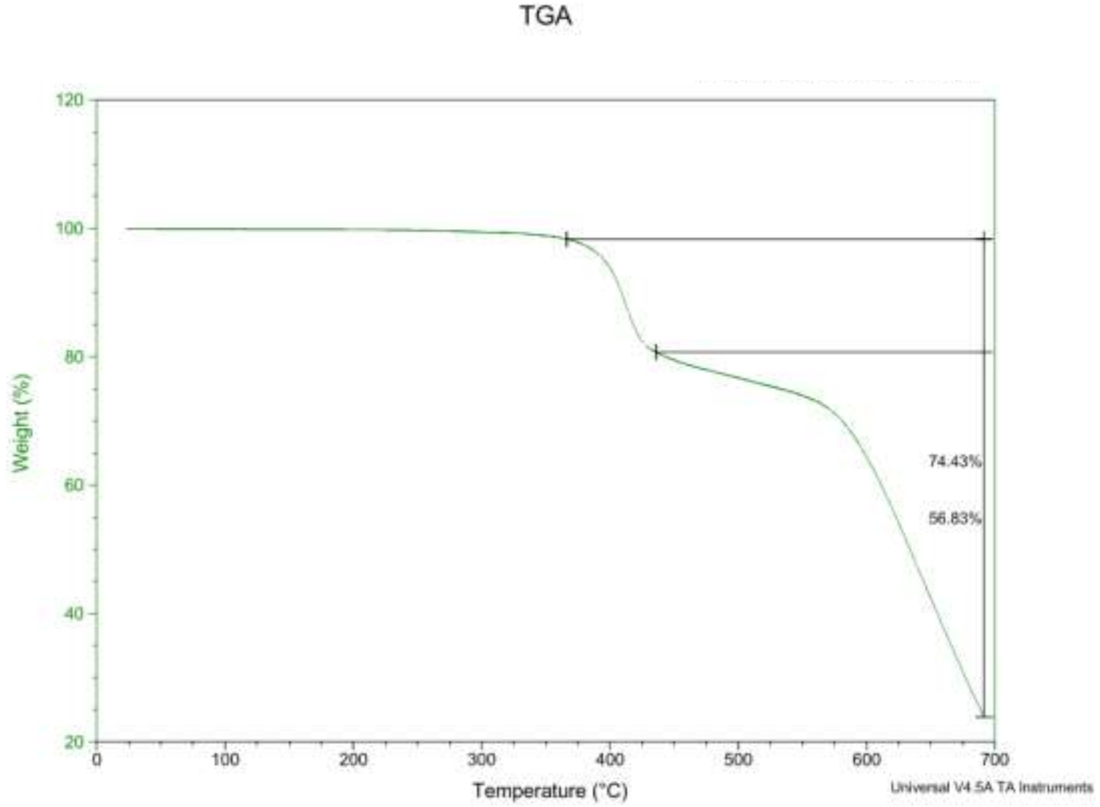

Figure S84. Dye 7

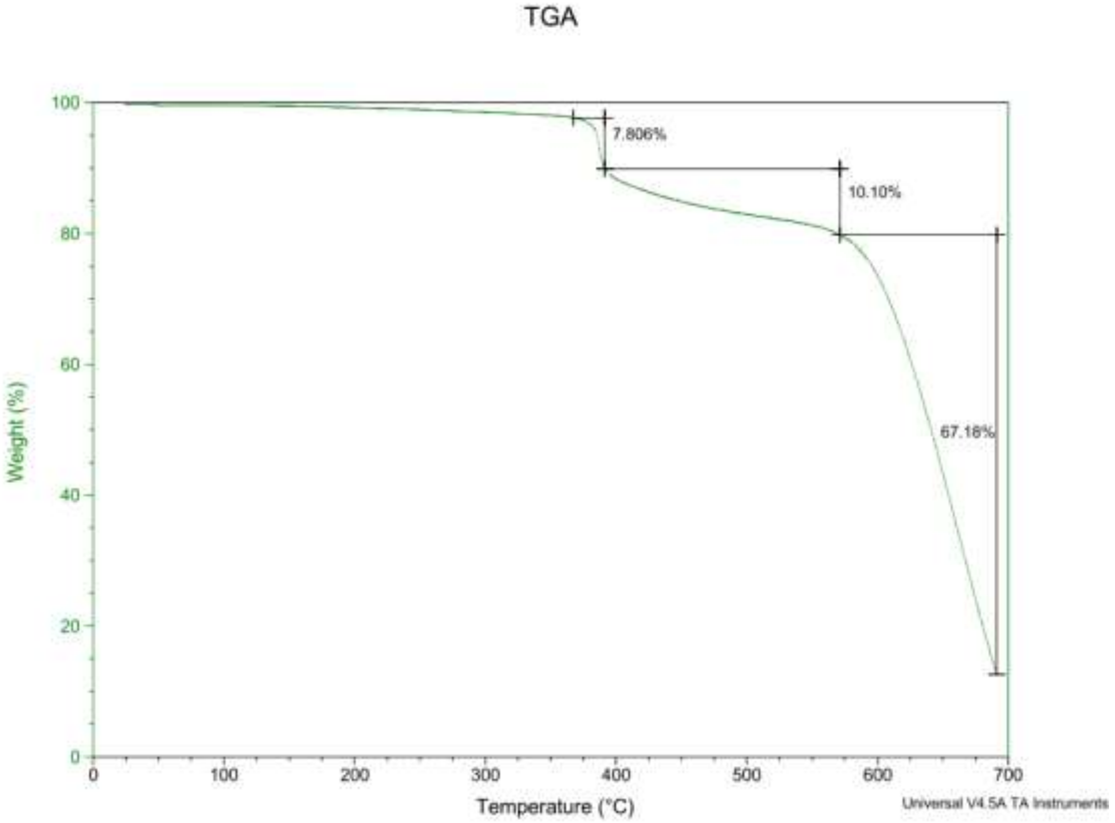

Figure S85. Dye 8

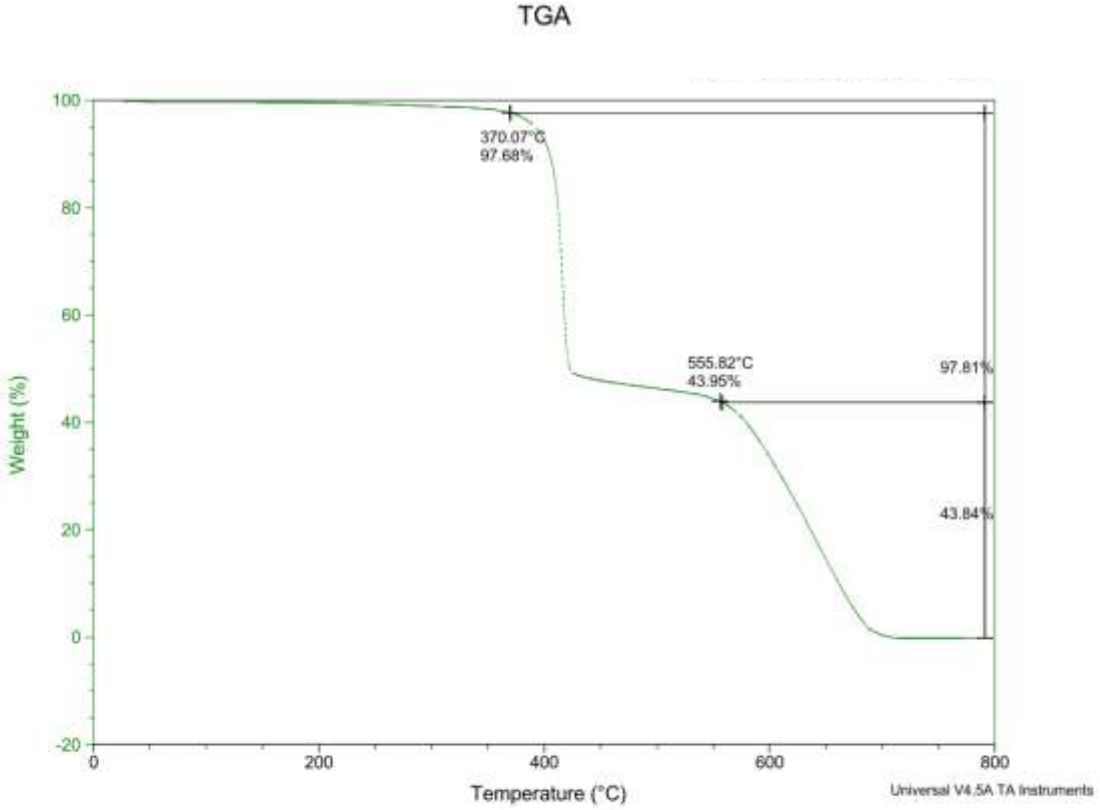

Figure S86. Dye 9

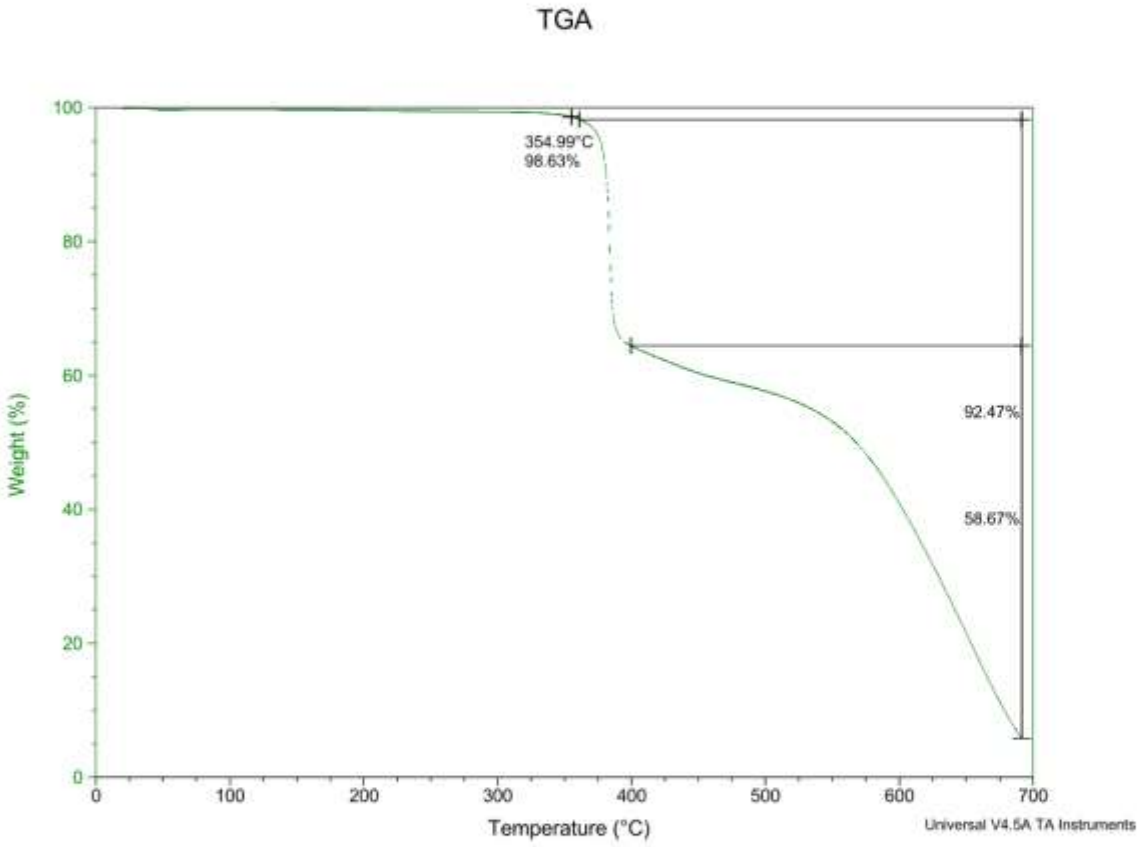

Figure S87. Dye 10

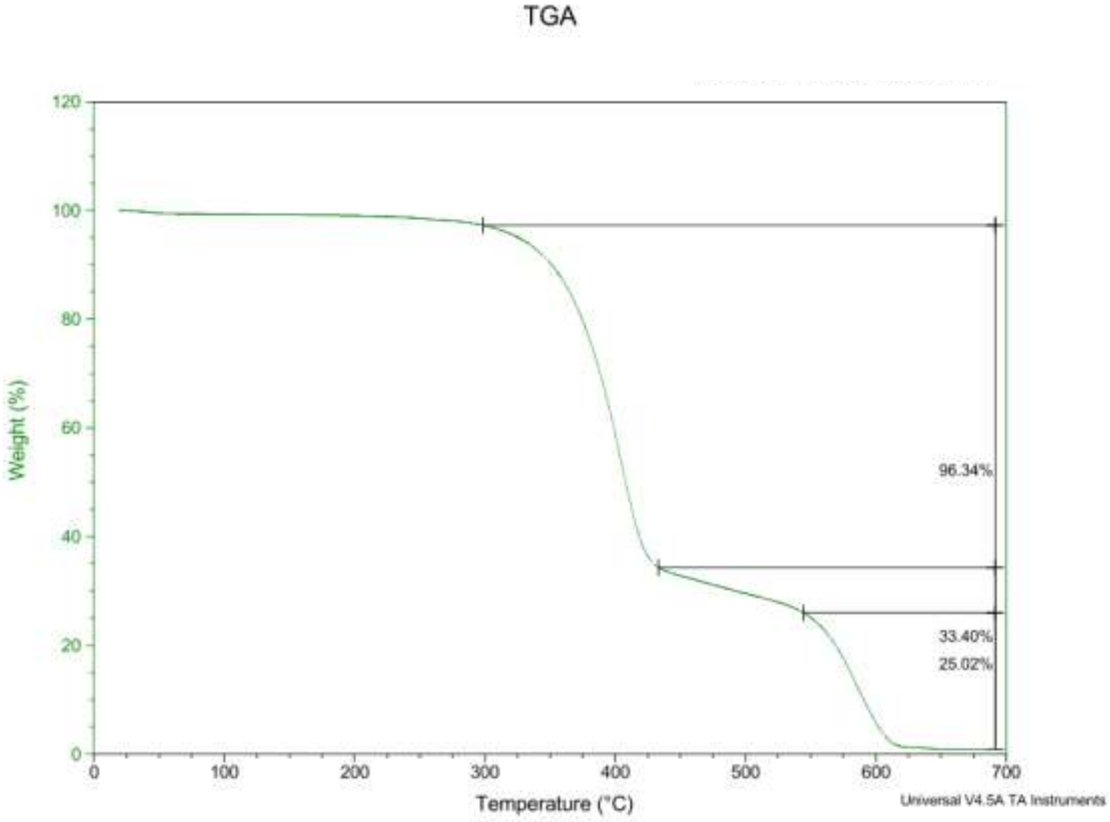

Figure S88. Dye 11

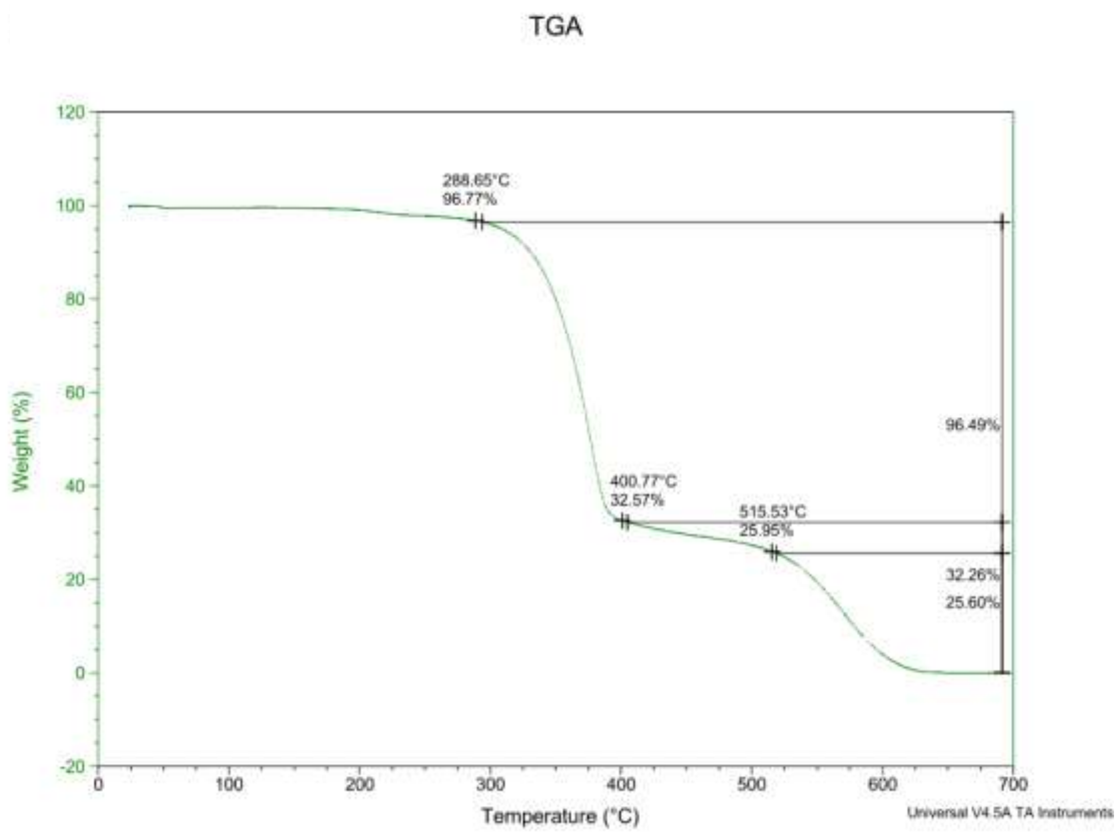

Figure S89. Dye 12

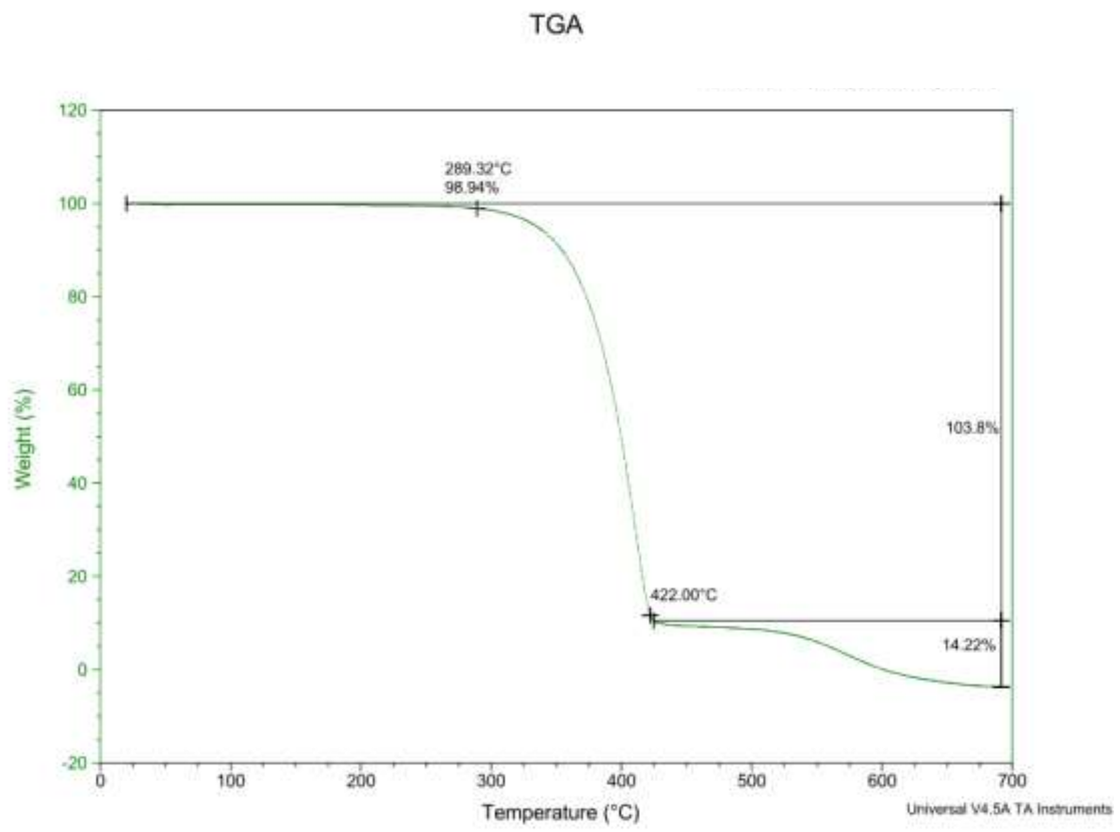

Figure S90. Dye 13

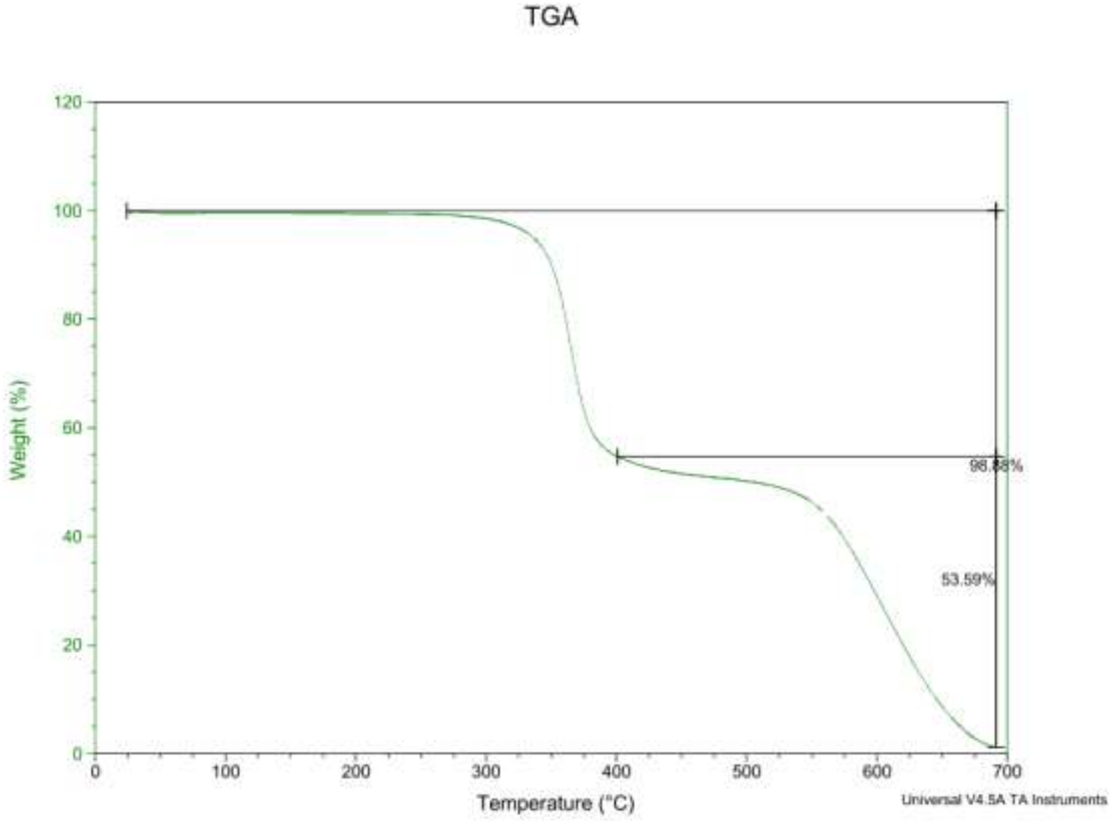

Figure S91. Dye 14

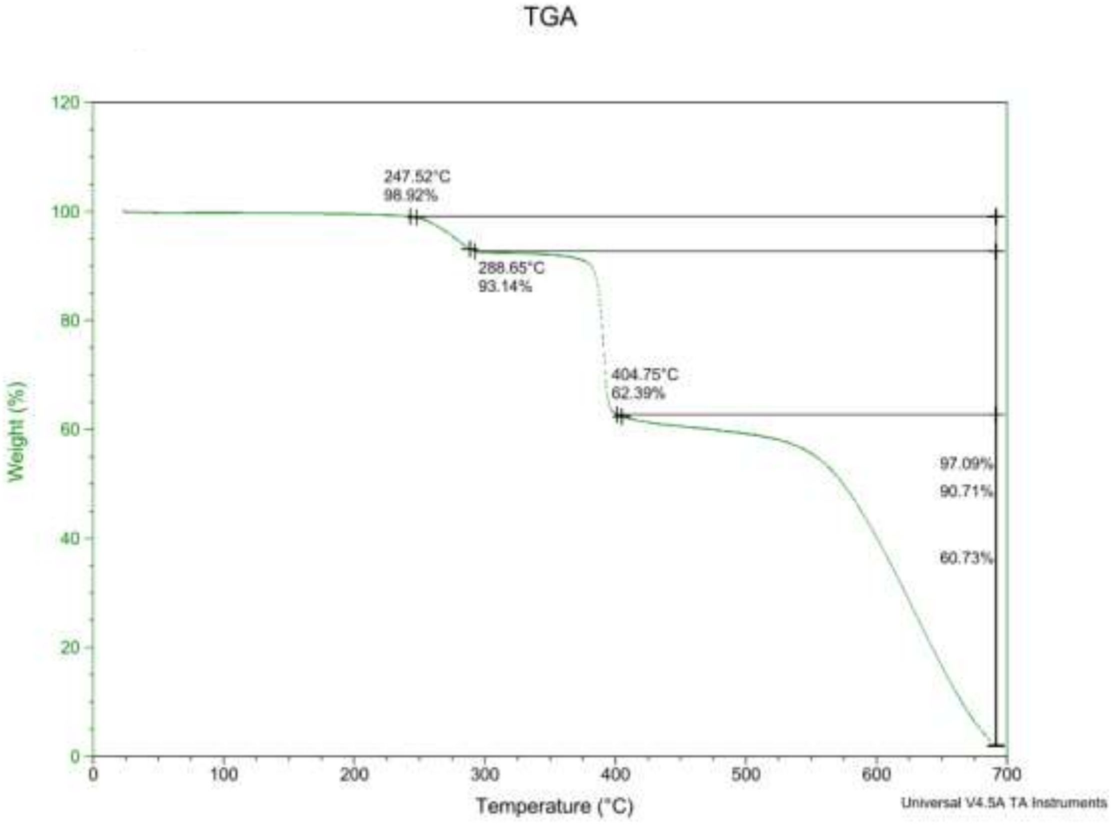

Figure S92. Dye 15

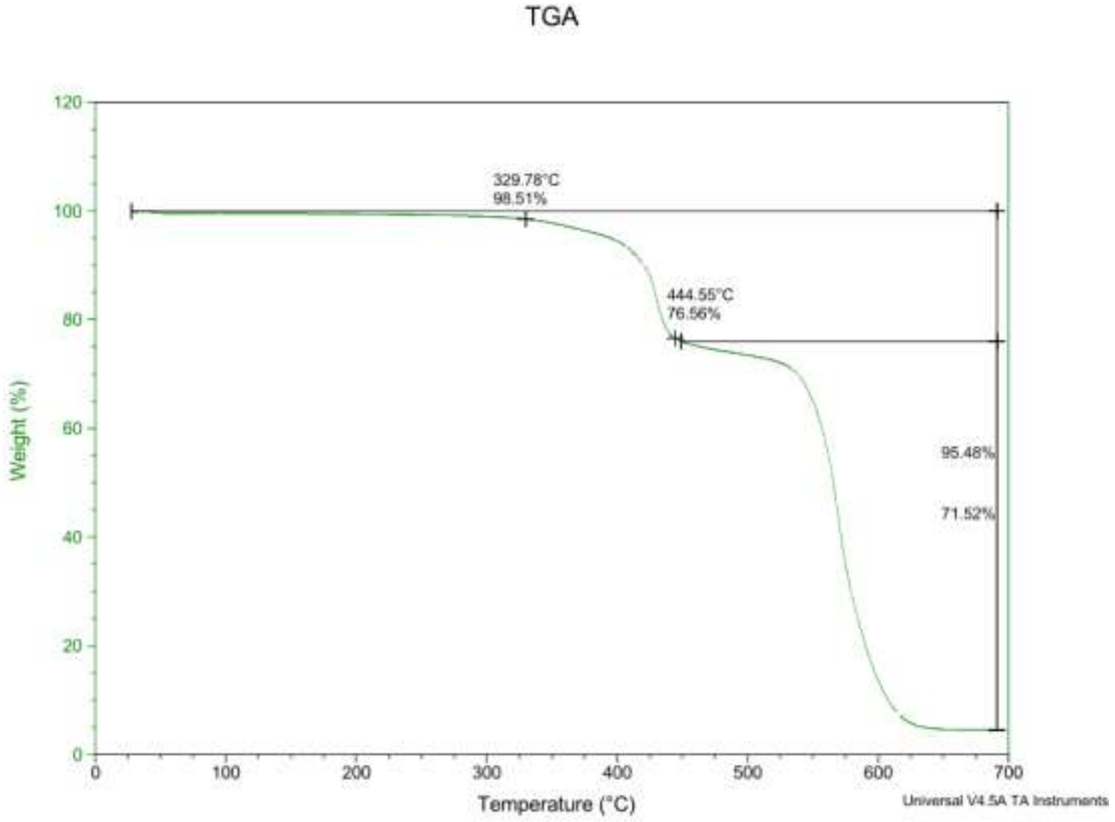

Supplement: Supplementary file 1 [file molecules-28-01489-s001.zip › molecules-2138671-supplementary.pdf]
